# Transcriptome-Based Construction of the Gibberellin Metabolism and Signaling Pathways in *Eucalyptus grandis* × *E. urophylla*, and Functional Characterization of *GA20ox* and *GA2ox* in Regulating Plant Development and Abiotic Stress Adaptations

**DOI:** 10.3390/ijms24087051

**Published:** 2023-04-11

**Authors:** Wenfei Wu, Linhui Zhu, Pan Wang, Yuwu Liao, Lanjuan Duan, Kai Lin, Xin Chen, Lijie Li, Jiajing Xu, Hao Hu, Zeng-Fu Xu, Jun Ni

**Affiliations:** 1 State Key Laboratory for Conservation and Utilization of Subtropical Agro-Bioresources, College of Forestry, Guangxi University, Nanning 530004, China; 2 Guangxi Key Laboratory of Forest Ecology and Conservation, College of Forestry, Guangxi University, Nanning 530004, China

**Keywords:** *Agrobacterium rhizogenes*, hairy root, root induction, salt stress, xylem development

## Abstract

Gibberellins (GAs) are the key regulators controlling plant growth, wood production and the stress responses in perennial woody plants. The role of GA in regulating the above-mentioned processes in *Eucalyptus* remain largely unclear. There is still a lack of systematic identification and functional characterization of GA-related genes in *Eucalyptus*. In this study, a total of 59,948 expressed genes were identified from the major vegetative tissues of the *E. grandis* × *E. urophylla* using transcriptome sequencing. Then, the key gene families in each step of GA biosynthesis, degradation and signaling were investigated and compared with those of *Arabidopsis*, rice, and *Populus*. The expression profile generated using Real-time quantitative PCR showed that most of these genes exhibited diverse expression patterns in different vegetative organs and in response to abiotic stresses. Furthermore, we selectively overexpressed *EguGA20ox1*, *EguGA20ox2* and *EguGA2ox1* in both *Arabidopsis* and *Eucalyptus* via *Agrobacterium tumefaciens* or *A. rhizogenes*-mediated transformation. Though both *Arabidopsis EguGA20ox1*- and *EguGA20ox2*-overexpressing (OE) lines exhibited better vegetative growth performance, they were more sensitive to abiotic stress, unlike *EguGA2ox1*-OE plants, which exhibited enhanced stress resistance. Moreover, overexpression of *EguGA20ox* in *Eucalyptus* roots caused significantly accelerated hairy root initiation and elongation and improved root xylem differentiation. Our study provided a comprehensive and systematic study of the genes of the GA metabolism and signaling and identified the role of *GA20ox* and *GA2ox* in regulating plant growth, stress tolerance, and xylem development in *Eucalyptus*; this could benefit molecular breeding for obtaining high-yield and stress-resistant *Eucalyptus* cultivars.

## 1. Introduction

*Eucalyptus* is one of the fastest growing tree species. It is widely planted in many tropical and subtropical regions for pulp and paper production. *Eucalyptus* hybrids (e.g., *E. grandis* × *E. urophylla*) combine fast growth with excellent wood quality and relatively high stress resistance; thus, they have been extensively used in commercial plantations, mostly in clonal forestry worldwide [1,2,3]. The excellent performance of *Eucalyptus* hybrids is the consequence of interactions between good genotype and environment [2]. Thus, ‘elite’ clones are the prerequisites for the *Eucalyptus* industry. Recent advancements in genomic and transcriptomic technologies have led to the sequencing of the *E. grandis* genome and the transcriptomes of several other *Eucalyptus* species [4,5,6,7,8,9]. Theoretically, the important economic characteristics of *Eucalyptus* could be “on-target” increased by using molecular breeding strategies. Nevertheless, genetic studies and molecular breeding in *Eucalyptus* are still lacking. The transcriptomic or genomic investigation of *E. grandis* × *E. urophylla* would help to clarify the formation of heterosis in the hybrid at the molecular level.

Gibberellins (GAs), a class of tetracyclic diterpenoid phytohormones, play an important role in many aspects of plant growth and development, including seed germination, wood formation, flowering, and stress adaptation [10,11,12]. In the past few decades, the metabolism and signaling of GAs have been well studied in many plant species, especially in *Arabidopsis* and rice. GA biosynthesis is a complicated pathway that is mainly catalyzed by seven key steps: ent-copalyl diphosphate synthase (CPS), ent-kaurene synthase (KS), ent-kaurene oxidase (KO), ent-kaurenoic acid oxidase (KAO), GA13-oxidase (GA13ox), GA20-oxidase (GA20ox), and GA3-oxidase (GA3ox) [10]. During this synthesis route, abundant GA intermediates (GA12, GA15, GA24, GA9, GA53, GA44, GA19, and GA20) are produced and then catalyzed into the bioactive forms (GA1, GA3, and GA4) at the final catalytic step by GA3ox. Although many gene families are involved in late GA synthesis, GA20ox seems to be the limiting step for GA synthesis. *GA20ox2* was previously regarded as the “green revolution” gene, the mutation of which caused a relative dwarf phenotype in rice, which led to yield improvement [13,14]. In hybrid aspen and tobacco, overexpression of *GA3ox* did not cause a significant increase in bioactive GAs [15,16]. In pea, overexpression of *GA3ox* influenced GA biosynthesis, but it was timing- or localization-dependent [17]. Previous studies also showed that overexpression of the early-step genes of GA biosynthesis, such as *CPS* and *KS*, did not affect the bioactive GAs but rather the GA intermediates [18,19]. Thus, it is apparent that *GA20ox*, but not *GA3ox* or other catalytic enzymes, are the rate-limiting enzymes in GA synthesis. Therefore, *GA20ox*s are the key targets for genetic engineering aimed at manipulating the important economic traits that are governed by GAs.

The deactivation of bioactive GAs is regulated by several distinct groups of enzymes, including GA2ox, CYP714D1, methyl transferase, and GA glucosyl transferase [10]. Among these, GA2ox-mediated catalysis of active GAs into nonactive forms has been the most universal and well-studied step in recent years [20,21]. GA2oxs belong to the 2OG-FE (II) oxygenase superfamily. Similar to the GA20ox and GA3ox families, GA2oxs are also encoded by a small group of genes, which can be divided into two major families, the members of which act primarily on C19-GA and C20-GA substrates [10,22]. To date, nine *GA2ox* genes have been identified in *Arabidopsis* [21], four in rice [23], eleven in *Populus* [24], five in tomato [25], seven in peach [26], and eight in *Liriodendrom chinense* [27]. In *L. chinense* and peach, different members of the *GA2ox* family exhibited diverse expression pattern in different plant tissues [26,27]. The expression of several *GA2ox*s, such as *LcGA2ox1*, *LcGA2ox4* and *LcGA2ox7*, was specially up-regulated by salt, osmotic, or chilling stresses [27]. Overexpression of *GA2ox* in tobacco reduced the bioactive GA content and thus induced the dwarfism phenotype [26]. As *GA2ox*s have essential functions in counterbalancing GA concentration, the flexible regulation of GA levels, achieved by the temporal and spatial expression of multiple *GA2ox* family members, is very important for maintaining regular plant growth and development and in responses to environmental clues [21].

To date, abscisic acid (ABA), jasmonic acid (JA), salicylic acid (SA), strigolactone (SL), and melatonin are the most well-studied phytohormones in the regulation of abiotic and biotic stress [28,29,30,31,32,33]. Although a few studies have reported that the biosynthesis and metabolism of GA are differentially expressed in response to biotic and abiotic stresses [34,35,36,37], the molecular mechanisms remain elusive. In *Populus*, several *GA2ox*s are specifically highly expressed in the roots, and overexpression of *GA2ox* modulates biomass accumulation and lateral root formation via interactions with other phytohormones [38,39]. In *Arabidopsis*, overexpression of cucumber *GA20ox1* promoted the primary root growth and increased the lateral root number [39]. Root architecture, including root length, number of lateral roots, and root hairs, is an important trait for plants to adapt to abiotic stresses [40]. Thus, GA-modulated plant root growth might also contribute to stress adaptation. Moreover, under abiotic stresses, the level of bioactive GAs could be immediately downregulated by inducing the expression of different *GA2ox*s [34,37,41]. Recent reports have shown that stress responsive *AtGA2ox9* and *AtGA2ox10* directly regulate plant adaptation to cold stress in *Arabidopsis* [42]. Thus, the stress-responsive modulation of endogenous bioactive GA levels and GA-regulated root architecture might also play an important role in regulating plant adaptation to environmental changes.

In several perennial woody plant species, GAs are important regulators that control multiple developmental aspects, including wood formation and stress adaptations. However, in *Eucalyptus*, the role of GA in regulating the above-mentioned processes largely remains elusive. In addition, there is still a lack of systemic identification and characterization of the key genes involved in GA synthesis, degradation, signaling, and of investigation of their molecular functions in the regulation of diverse aspects of plant growth and development. Based on the third-generation transcriptome sequencing strategy by PacBio Iso-Seq, the genome-wide expressed genes in the main vegetative organs of *E. grandis* × *E. urophylla* were identified. Based on this, we comprehensively identified the key genes involved in GA synthesis, degradation, and early signaling components and constructed GA-related networks. In addition, we also investigated the expression pattern of the main GA synthesis and degradation genes in response to abiotic stresses (salt and osmotic). To further investigate the molecular functions of the identified *EguGA20ox* and *EguGA2ox* genes in *E. grandis* × *E. urophylla*, we selectively cloned and overexpressed *EguGA20ox1*, *EguGA20ox2*, and *EguGA2ox1* in *Arabidopsis* and *Eucalyptus* and analyzed their role in controlling plant growth, root initiation and growth, xylem development, and abiotic stress responses. This study not only systematically identified the key gene families involved in the regulation of GA synthesis, degradation, and signaling for the first time, based on third-generation transcriptome sequencing, but also provides evidence that GA is stress-responsive and might directly regulate stress adaptation via regulation of GA homeostasis in *Eucalyptus*. Therefore, this study aimed to identify GA-related gene families and their expression patterns in response to environmental factors, and to characterize the functions of key GA metabolism-related genes; this identification and characterization could greatly improve our understanding of the role of GA in the regulation of plant growth and stress-adaptation in *Eucalyptus*, and facilitate the selection of high-yield and stress-resistant cultivars though genetic modification strategies.

## 2. Results

### 2.1. Transcriptome Sequencing of Five Major Vegetative Organs of E. grandis × E. urophylla

**Figure 1:**
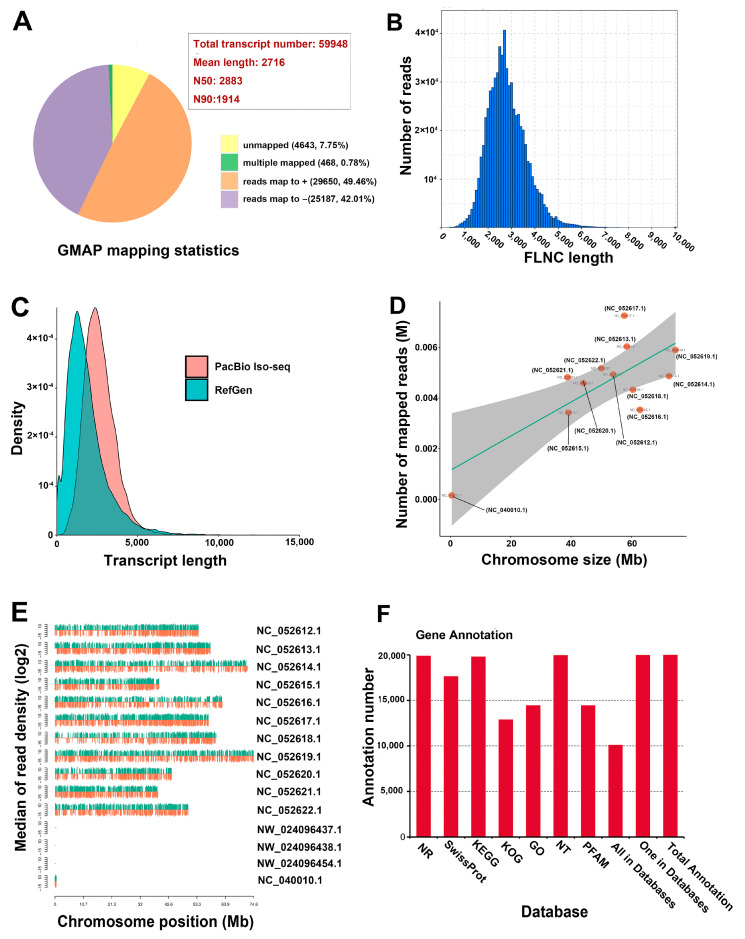
Overview of the transcriptome sequencing of the five major vegetative organs of *Eucalyptus grandis* × *E. urophylla*. (**A**) Statistics of GMAP mapping. (**B**) FLNC length distribution of the transcripts. (**C**) Comparison of the transcript length distribution against the reference genome of *E. grandis*. (**D**) Number of the mapped reads in different chromosomes of *E. grandis*. (**E**) Reads density in different chromosomes. (**F**) Gene annotation in different databases.

The vegetative organs of *Eucalyptus*, such as wood and fibers, as well as cineole, are the primary resources for industrial purposes. To investigate the global gene expression in the vegetative organs of *E. grandis* × *E. urophylla*, tissues from the stem, root, mature leaf, young leaf, and shoot apex were collected for transcriptome sequencing. The third-generation transcriptome sequencing platform PacBio Iso-Seq was used for the analysis. A total of 71.59 Gb (approximately 100-fold the *E. grandis* genome size) of subreads were obtained, and a total of 26 M subreads were collected. After error correction, a total of 59,948 consensus transcripts were obtained, with a mean length of 2716 bp, N50 of 2883, and N90 of 1914 (Figure 1A). The read length range is shown in Figure 1A, in which the most abundant read length was from 2000 to 4000 bp (Figure 1B). As the genomes of *E. grandis* × *E. urophylla* and *E. urophylla* are lacking, the consensus transcripts were mapped to the genome of one of the hybrid’s parents, *E. grandis*. The results showed that 91.47% of the transcripts could be mapped to the *E. grandis* genome (sequence similarity >97%), 0.78% were multiple-mapped, and 7.75% were unmapped (Figure 1A). The genome of *E. grandis* × *E. urophylla* included both parental genomes; thus, we postulated that 7.75% of unmapped transcripts could be specifically transcribed from the *E. urophylla* genome. Nevertheless, the possibility that *E. grandis* and *E. urophylla* might share high sequence similarity of many genes in each other’s genomes cannot be ruled out. Moreover, the results showed that the density of the transcript length of *E. grandis* × *E. urophylla* was higher than that of *E. grandis* (Figure 1C). A total of 91.47% of the transcripts were finely mapped to the eleven *E. grandis* chromosomes, and some transcripts were mapped to the mitochondrial genome (Figure 1D).

Typically, with increasing chromosome size, the number of expressed genes would increase accordingly. Interestingly, NC_052619.1 and NC_052618.1 were the largest chromosomes, but the number of mapped reads of these two chromosomes were significantly lower than that of NC_052617.1. NC_052621.1 was the smallest chromosome, but the number of mapped transcripts was no less than that of NC_052614.1 (Figure 1D,E). These results suggested that the gene intensity might not always be in accordance with chromosome size. However, we cannot rule out the possibility that some high-similarity genes from the *E. urophylla* chromosomes were also mapped to their homologs in the *E. grandis* chromones, which might have caused the changed gene density at the chromosome level. Gene annotation of all the mapped and unmapped transcripts was then carried out using seven gene annotation databases: NR, NT, Pfam, KOG/COG, SwissProt, KEGG, and GO. A total of 18,957 mapped transcripts and 4643 unmapped transcripts were annotated (Figure 1F); this facilitated further characterization of the gene functions of *E. grandis* × *E. urophylla*.

### 2.2. Identification of Key Gene Families in Each Step of GA Metabolism and Signaling

**Figure 2:**
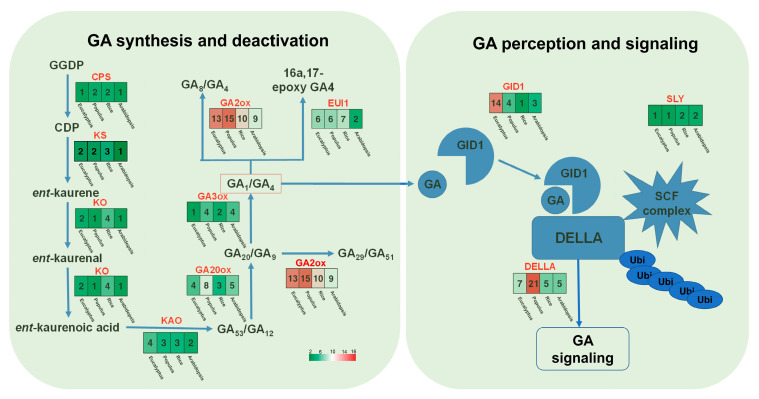
Overview of the gene families in the synthesis, degradation, and signaling of gibberellin. The number of the family members in *E. grandis × E. urophylla*, *Populus*, *Oryza sativa*, and *Arabidopsis* was showed in the heatmaps.

**Figure 3:**
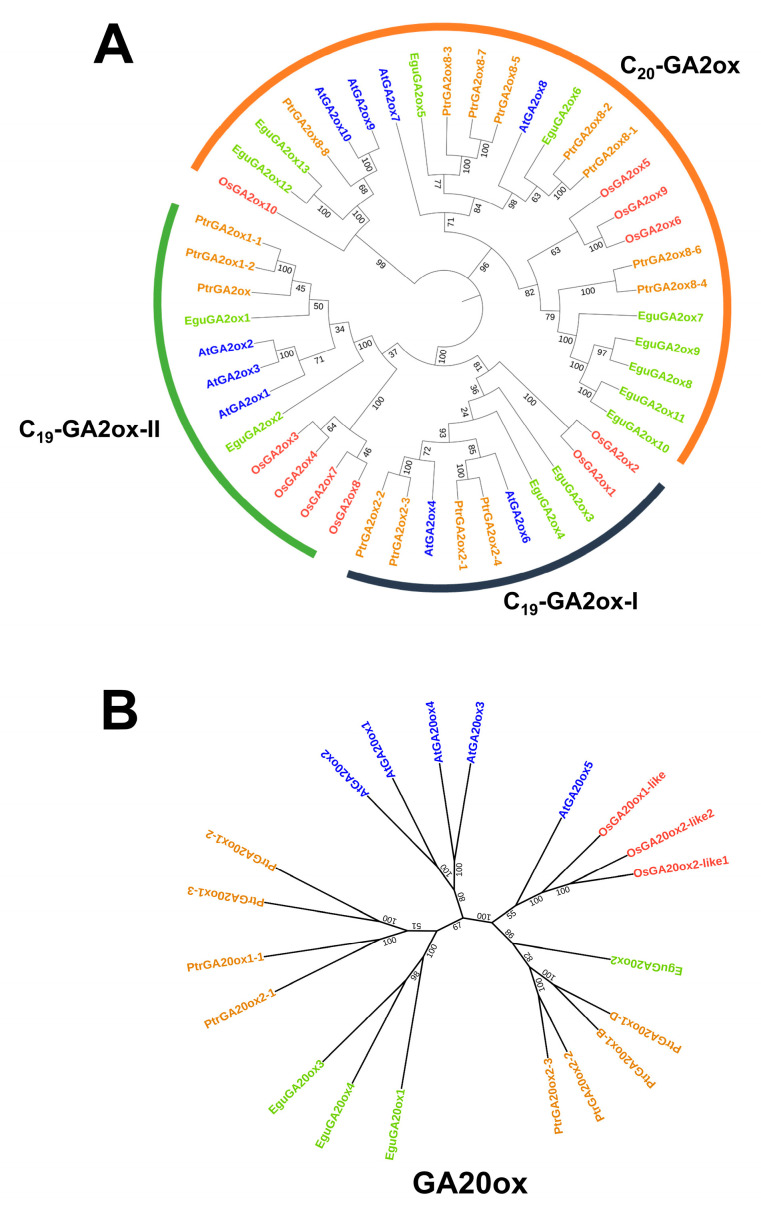
Phylogenetic analysis of GA2ox (**A**) and GA20 (**B**) families of different plant species (*E. grandis* × *E. urophylla*, *Arabidopsis thaliana*, *Oryza sativa*, and *Populus trichocarpa*). The sequences were aligned using the Mega 5.0 software. A maximum likelihood tree was calculated based on the alignment and evaluated using 1000 bootstrap repetitions.

The *E. grandis × E. urophylla* hybrid exhibits excellent growth rate, wood quality, and abiotic stress resistance. As GA is a multifaceted phytohormone that directly or in part regulates the abovementioned traits [10,11,12,43], investigation of the role of GA in *Eucalyptus* might help to clarify the molecular mechanism governing the formation of these economic traits and further facilitate genetic modifications. Although the genes of the main steps in GA biosynthesis, degradation, and signaling have been well studied in *Arabidopsis* and rice in recent decades [10], there is still a lack of integrative investigation of the whole pathway based on genomic and transcriptomic data for most species, including *Eucalyptus*. Here, using the protein sequences of *Arabidopsis* CPS, KS, KO, KAO, GA20ox, GA3ox, GA2ox, GID, and DELLAs as the blast query for BLAST analysis, together with conserved domain analysis using TBtools [44], we identified their potential homologs in the transcriptome of *E. grandis* × *E. urophylla*. Then, the synthesis, degradation, and signaling routes of *Arabidopsis*, rice, *Populus trichocarpa*, and *E. grandis × E. urophylla* were constructed and compared (Figure 2). It can be clearly seen that the number of family members in some GA-related gene families was conserved, including CPS, KS, and KAO (Figure 2). In this *Eucalyptus* hybrid, a total of 14 GA receptor *GID* genes were identified, whereas there was only one in rice, three in *Arabidopsis*, and four in *Populus* (Figure 2). Among all the families, *GA2ox* was one of the largest gene families in these species, with 15 members in *Populus*, 13 in *E. grandis* × *E. urophylla*, 10 in rice, and 9 in *Arabidopsis* (Figure 2). Moreover, phylogenetic analysis was carried out to investigate the phylogenetic relationships of the GA2ox and GA20ox families among these four plant species (Figure 3). The results showed that in *E. grandis* × *E. urophylla*, the 13 members of EguGA2oxs can be divided into two major types, C19-GA2ox and C20-GA2ox (Figure 3A), which is similar to that in peach [26]. Moreover, the phylogenetic tree also showed that C19-GA2ox can be further divided into two clades, C19-GA2ox-I and C19-GA2ox-II (Figure 3A). The results also showed that, with the exception *EguGA20ox2*, the other *EguGA20ox*s showed lower genetic homology with the GA20oxs from the other three species (Figure 3B). These phylogenetic trees suggested the diverse evolutionary formation processes of different GA2ox and GA20ox members and indicated the functional differences among different members.

### 2.3. Expression Profile of the Genes Involved in the Regulation of GA Metabolism in Different Vegetative Organs

**Figure 4:**
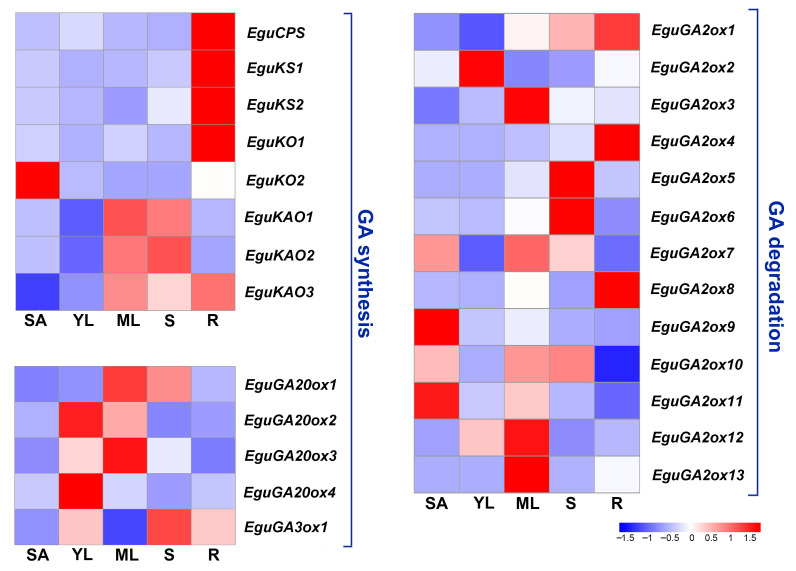
Gene expression profile of the GA synthesis and degradation genes in five different vegetative organs of *E. grandis* × *E. urophylla*. The expression level of each gene was normalized to the reference genes *PP2A-1* and *PP2A-3*. The heatmaps were plotted using data obtained by RT-qPCR with three biological replicates. SA, shoot apex; YL, young leaf; ML, mature leaf; S, stem; R, root.

Different members of a family might have similar functions in enzymatic activities or signal transduction; however, they might be temporally or spatially regulated in plants to achieve more precise and flexible regulation, as with the *GA2ox* family genes in peach and *Liriodendron chinense* [26,27]. In *Eucalyptus*, each step of GA metabolism is regulated by multiple gene members. To further investigate the expression pattern of these genes, Real-time quantitative PCR (RT qPCR) was used to survey the expression pattern of all the identified GA metabolism-related genes in five major vegetative organs of *Eucalyptus* (root, stem, mature leaf, young leaf, and shoot apex). The results showed that several early GA synthesis genes, such as *EguCPS*, *EguKS1*, *EguKS2* and *EguKO1*, were highly expressed in the root, while *EguKAO1* and *EguKAO2* were mainly expressed in the stem and mature leaf in the *E. grandis* × *E. urophylla* seedlings (Figure 4). *EguKAO3* showed relatively high expression in both mature leaves and roots (Figure 4). GA20ox is the key rate-limiting enzyme catalyzing the synthesis of GA intermediates. The results showed that four *EguGA20ox*s only showed high expression levels in the leaves, with *EguGA20ox1* and *EguGA20ox3* in the mature leaves and *EguGA20ox2* and *EguGA20ox4* in the young leaves. Thirteen *EguGA2ox*s also showed diverse expression patterns in the five different organs. *EguGA2ox1*, *4*, and *8* were found to be highly expressed in the root, *EguGA2ox2* in the young leaf, *EguGA2ox3*, *12*, and *13* in the mature leaf, *EguGA2ox5* and *6* in the stem, and *EguGA2ox9* and *11* in the shoot apex (Figure 4).

### 2.4. Diverse Expression Patterns of GA Metabolism-Related Genes in Response to Abiotic Stress

**Figure 5:**
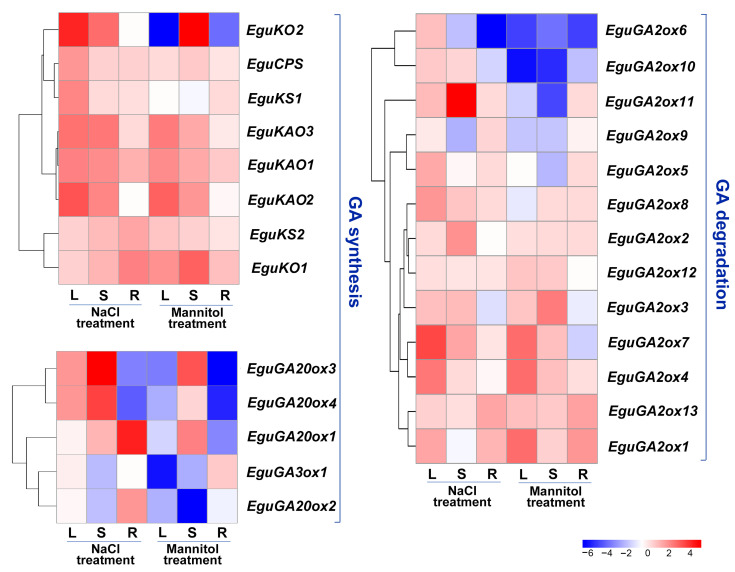
Gene expression profile of the GA synthesis and degradation genes in the leaves, stems, and roots of the three-month-old *E. grandis* × *E. urophylla* seedlings at 24 h after being subjected to salt (200 mM NaCl) or mannitol (300 mM) treatment. The expression level of each gene was normalized to the reference genes *PP2A-1* and *PP2A-3.* The heatmaps were plotted using data obtained by RT-qPCR with three biological replicates. L, leaf; S, stem; R, root.

Gene expression patterns are closely related to biological functions. We further investigated the expression of GA metabolism genes in response to abiotic stresses (salt and osmotic stress) in three different organs (leaf, stem, and root) of *E. grandis* × *E. urophylla* seedlings at 24 h after treatment with 200 mM NaCl and 300 mM mannitol, respectively. The results showed that the expression of early GA synthesis genes, including *EguCPS*, *EguKO*, *EguKS*, and *EguKAO*, was mostly upregulated in one or more tissues in response to either stress treatment, except that *EguKO2* was downregulated in the leaves and roots after mannitol treatment (Figure 5). Four *EguGA20ox* genes exhibited diverse responses to both stresses, whereas the expression of *EguGA3ox1* remained unaffected or was inhibited in response to the stresses (Figure 5). *GA2ox* genes play a pivotal role in counterbalancing bioactive GAs. Most of the *EguGA2ox* genes were found to be upregulated in response to the stresses (Figure 5), suggesting that GA might be negatively regulated in response to the stresses in *Eucalyptus*.

### 2.5. Overexpression of EguGA20ox Promoted Plant Growth and Biomass Accumulation in Arabidopsis

**Figure 6:**
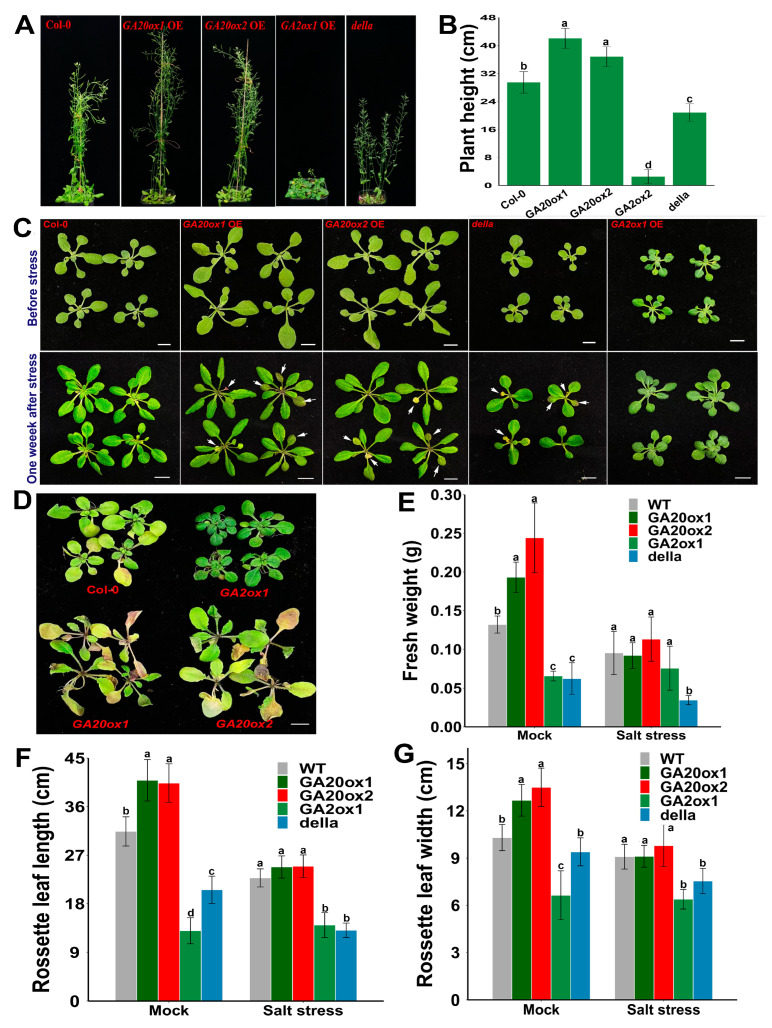
Overexpression of either *EguGA20ox1* or *EguGA20ox2* promoted vegetative growth but decreased salt stress tolerance in *Arabidopsis*. (**A**) Plant growth status of different *Arabidopsis* lines at 45 d. (**B**) Plant height at 45 d (*n* = 20). (**C**) Plant status of different *Arabidopsis* lines before (upper layer), and one week after salt stress treatment (under layer). (**D**) Plant growth status two weeks after stress treatment. Fresh weight (**E**), leaf length (**F**), and width (**G**) of the mature rosette leaves of different *Arabidopsis* lines were calculated at one week after mock or stress treatment (*n* > 10). Data represents as mean ± sd. In each group, different lowercase letters represent the degree of significant difference (*p* < 0.05). Bars = 1 cm.

The expression of several GA metabolism-related genes was diversely regulated in response to abiotic stress treatment. Specifically, several *EguGA2ox*s, including *EguGA2ox1*, *3*, *4*, *7*, and *11*, were upregulated, whereas *EguGA20ox2*, *3*, and *4* were downregulated after stress treatment (Figure 5). This stress-responsive expression pattern of the GA-metabolism genes in *Eucalyptus* is similar to that in *Liriodendron chinense*, where the expression of several *GA2oxs* was significantly up-regulated in response to different abiotic stresses [27]. Thus, we postulated that GA might be a negative regulator in controlling stress adaptations. To further investigate the molecular functions of the genes involved in GA synthesis (*EguGA20ox*) or catabolism (*EguGA2ox*), we selectively cloned and overexpressed *EguGA20ox1*, *EguGA20ox2*, and *EguGA2ox1* in *Arabidopsis*. For each gene, over ten independent transgenic *Arabidopsis* lines with similar phenotypes were obtained. The gene expression of the transgenic *Arabidopsis* lines was analyzed by RT-qPCR, as shown in Figure S1. The results showed that overexpression of either of the *EguGA20oxs* significantly increased plant height and caused an early flowering phenotype, while *EguGA2ox1*-overexpressing (OE) lines exhibited a delayed flowering phenotype (Figure 6A,B), suggesting a classic role of GA in the promotion of flowering in *Arabidopsis*. Surprisingly, we also detected that overexpression of either *EguGA20ox1* or *EguGA20ox2* led to significantly enhanced vegetative growth, which resulted in enlarged leaf size and increased biomass accumulation (Figure 6C–G). The GA signal is primarily transduced via the degradation of DELLA proteins. In this study, we discovered that the *della* mutant did not exhibit similar phenotypes to the *EguGA20ox1*- or *EguGA20ox2*-OE lines, except for the early flowering phenotype (Figure 6A). Due to early flowering, the final number of rosette leaves of the *EguGA20ox* lines was lower than that of the Col-0 plant (Figure S2). Nevertheless, before the plant started bolting, the number of leaves of either *EguGA20ox* line was significantly greater than that of Col-0 or *della* mutant (Figure S2).

### 2.6. Overexpression of EguGA20ox and EguGA2ox in Arabidopsis Led to Diverse Responses to Salt Stress

Salt stress treatment was used to investigate the abiotic stress tolerance of *EguGA20ox1*, *EguGA20ox2*, and *EguGA2ox1* transgenic lines. Seven days after subjecting the roots to treatment with 200 mM NaCl, the growth status of *EguGA20ox1*, *EguGA20ox2*, *EguGA2ox1*, and Col-0 plants was recorded. The results showed that both the *EguGA20ox1*- and *EguGA20ox2*-OE lines were hypersensitive to salt stress and had a more severe leaf decoloring and withering phenotype, while the *EguGA2ox1*-OE line exhibited higher stress tolerance one week after treatment (Figure 6C,D). Due to stress hypersensitivity, the biomass and leaf size of the *EguGA20ox*-OE lines were more likely to be reduced in response to salt stress, while plant growth seemed to be unaffected in *EguGA2ox1* plants (Figure 6E–G).

**Figure 7:**
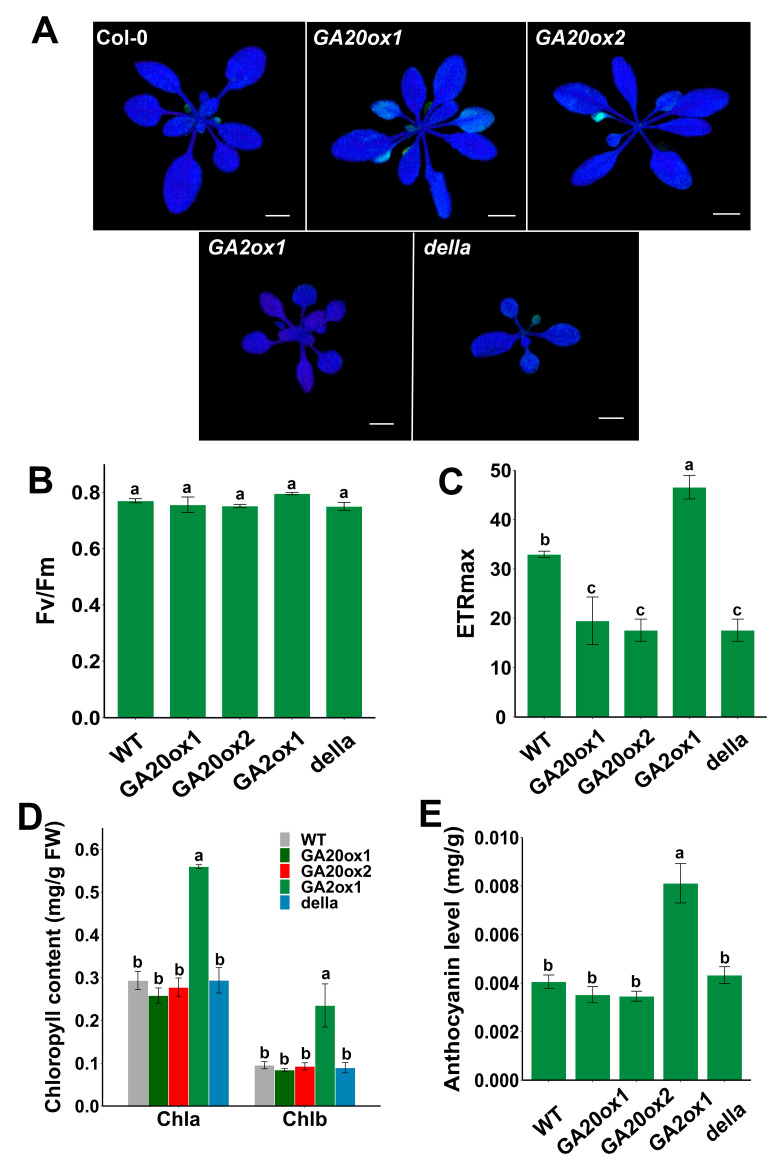
*EguGA2ox1* overexpression increased the maximum electron transport ratio and the content of chlorophyll and anthocyanin in the leaf. (**A**) Picture of the photosynthetic inflorescence. Fv/Fm (**B**) and ETRmax (**C**) of different *Arabidopsis* lines one week after salt stress treatment (*n* = 4). (**D**,**E**) Chlorophyll and anthocyanin content of three-week-old *Arabidopsis* seedlings (*n* = 3). Data represents as mean ± sd. Different lowercase letters represent the degree of significant difference (*p* < 0.05). Bars = 1 cm.

The photosynthesis parameters were analyzed to further clarify whether overexpression of *EguGA20ox* or *EguGA2ox* could alter the key photosynthetic parameters, which were also regarded as important markers for stress tolerance. The results showed that salt stress could impair the photosynthetic ability of *EguGA20ox*-OE plants; this was reflected by the decreased chlorophyll fluorescence in some leaves (Figure 7A). Intriguingly, the quantum efficiency of photosystem II (Fv/Fm) of *EguGA20ox* was not significantly affected but was only slightly lower than that of *EguGA2ox1* (Figure 7B). However, it can be clearly seen that the maximum electron transport rate (ETRmax) of *EguGA20ox1-OE*, *EguGA20ox1-OE*, and *della* plants was approximately two-fold lower than that of Col-0 plants, while *EguGA2ox1-OE* exhibited the highest ETRmax. These photosynthetic parameters reflected the higher tolerance to salt stress of the *EguGA2ox1*-OE lines. Moreover, we also found that the *EguGA2ox1* plants exhibited much higher chlorophyll (chla and chlb) and anthocyanin levels (Figure 7D,E). As anthocyanins are the key antioxidants [45], the significantly higher anthocyanin level in *EguGA2ox1* plants greatly facilitates adaptations to severe environmental stresses (Figure 6).

### 2.7. GA20ox Promotes Root Initiation and Lateral Root Growth in Eucalyptus

**Figure 8:**
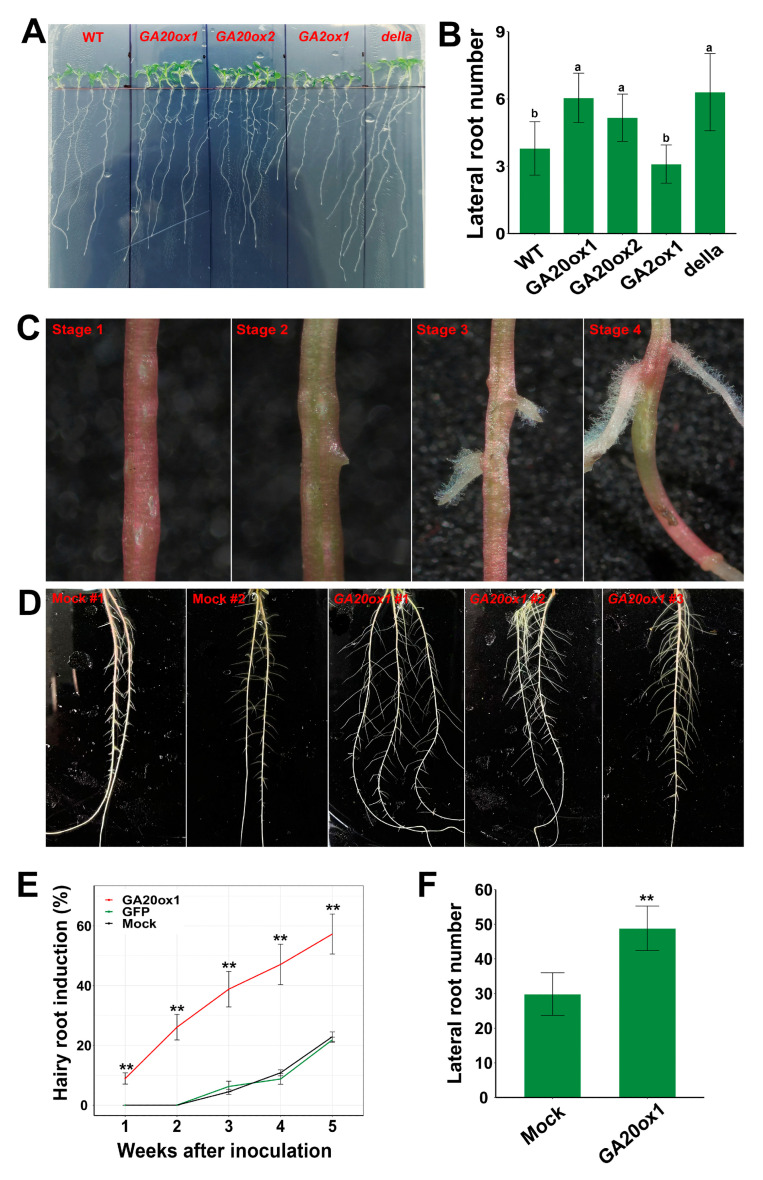
Overexpression of *EguGA20ox* in *Arabidopsis* and *Eucalyptus* improved the root growth. (**A**,**B**) Lateral root number of different *Arabidopsis* lines was counted two weeks after germination on the MS plates (*n* = 12). (**C**) Four stages of the hairy root induction in *Eucalyptus* seedlings. (**D**) Root growth status after growing in the liquid culture for two weeks. (**E**) Hairy root induction ratio after transformation with mock, GFP, or *EguGA20ox1* (*n* = 3, 90 seedlings for each replicate). (**F**) Lateral root number of the transgenic hairy roots of *E. grandis* overexpressing *EguGA20ox1* (*n* = 10). Data represents as mean ± sd. **, *p* < 0.01. Different lowercase letters represent the degree of significant difference (*p* < 0.05).

In addition to the significant promotive role of *EguGA20ox* in shoot growth, we also detected that the expression of *EguGA20ox* and *EguGA2ox* affected root development in *Arabidopsis*. Previous reports showed that GA could directly affect lateral root growth but exhibited diverse effects in different species [38,39]. Here, our results showed that the number of lateral roots of the *EguGA20ox1*- and *EguGA20ox2*-OE *Arabidopsis* lines was significantly increased, while the *EguGA2ox1*-OE line exhibited the lowest number of lateral roots among all tested *Arabidopsis* lines (Figure 8A,B). GA signal transduction was mostly dependent on the degradation of DELLA proteins. The results also revealed that the *della* mutant also showed an increase in the number of lateral roots. These results suggested that GA might be positively correlated with lateral root development in *Arabidopsis*.

The gene expression profile of the GA biosynthesis and metabolism genes in different organs of *Eucalyptus* showed that several early GA synthesis genes (*EguCPS*, two *EguKS*s, and *EguKO1*) and GA degradation genes (*EguGA2ox1*, *EguGA2ox4*, and *EguGA2ox8*) exhibited high expression levels in the root (Figure 4), suggesting that GA might be important for root growth and development in *Eucalyptus*. Here, we used *Agrobacterium rhizogenes*-mediated *Eucalyptus* transformation to investigate whether overexpression of *EguGA20ox* had any effects on root development in *E. grandis* seedlings. In comparison with two control groups that were separately inoculated with *A. rhizogenes* K599 or K599 harboring the 35S::GFP plasmid, the seedlings inoculated with *EguGA20ox1* (K599) showed significantly accelerated hairy root induction at one week after inoculation at the seedling hypocotyl (Figure 8C); it took almost one month for the control groups to start root formation. Within 3 to 4 days after inoculation with *EguGA20ox1* (K599), obvious callus formation can be clearly seen at the inoculation site (Stage 1). One week later, some inoculated seedlings showed obvious lateral root growth (Stage 2). Frequently, more than one lateral root could be induced at the wound site by *EguGA20ox1* (indicated in Figure 8C, Stage 3). Two weeks after inoculation, the hairy root induction ratio reached 25%, and a more significant increase (to 47%) was observed at four weeks (Figure 8E). These results suggested that overexpression of *EguGA20ox1* conferred fast *A. rhizogenes*-induced hairy root formation.

For both *EguGA20ox1* and mock-inoculated seedlings, the plants were then transplanted into liquid medium one week after hairy root induction for further analysis of root growth and development. Two weeks after transplantation, the hairy roots overexpressing *EguGA20ox1* showed a significantly increased number of lateral roots (Figure 8F), suggesting the potential pivotal role of GA in the regulation of lateral root growth in *Eucalyptus*. These results suggest that GA might play a pivotal role in the regulation of root growth and development in *Eucalyptus*. Nevertheless, the molecular mechanisms of the GA-regulated signaling pathway governing root growth in *Eucalyptus* require further investigation.

### 2.8. Overexpression of GA20ox1 Significantly Promoted Xylem Development in Eucalyptus

**Figure 9:**
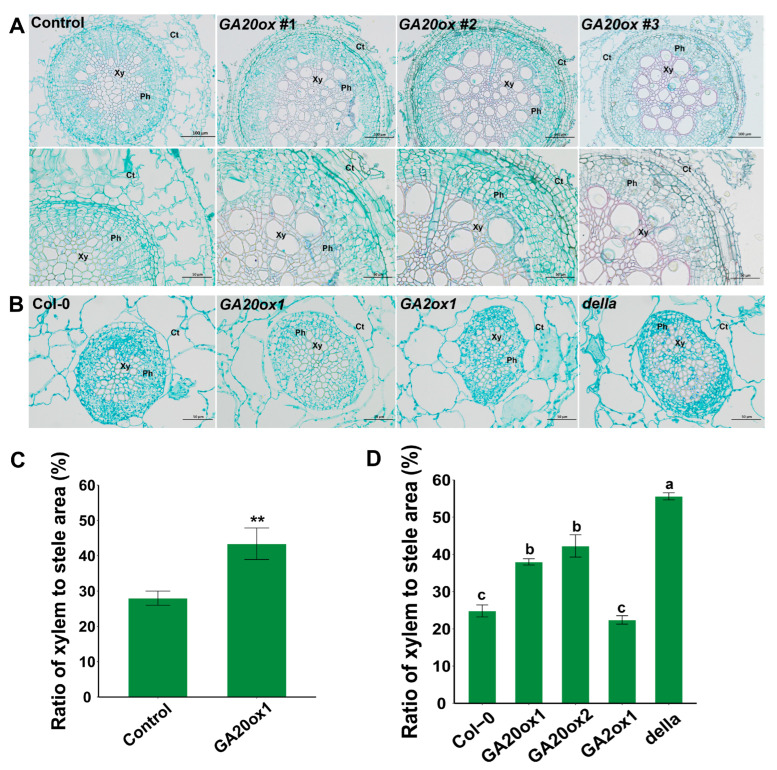
Overexpression of *EguGA20ox1* promotes xylem development in *Eucalyptus* and *Arabidopsis*. (**A**) Cross-section of the hairy root of control and *EguGA20ox1* OE lines (upper layer, 50-fold enlarged, under layer, 100-fold enlarged). (**B**) Cross-section of the hypocotyl of three-week-old *Arabidopsis* seedlings. Ratio of xylem to stele area of *Eucalyptus* (**C**) and *Arabidopsis* (**D**) (*n* > 4). Data represents as mean ± sd. **, *p* < 0.01. Different lowercase letters represent the degree of significant difference (*p* < 0.05). Xy, xylem; Ph, phloem; Ct, cortex.

In *Arabidopsis* and some other perennial woody plants, such as *Populus*, GA is one of the most important regulators involved in xylem development [10]. Nevertheless, the role of GA in controlling woody tissue formation has scarcely been studied in *Eucalyptus*. After two weeks in liquid medium, the hairy roots of mock- and *EguGA20ox1*-OE plants, as shown in Figure 8D, were used for histochemical microscopic examination. The results showed that overexpression of *EguGA20ox1* significantly improved xylem differentiation in the transgenic *Eucalyptus* hairy roots (Figure 9A); this is consistent with previous results in another tree species hybrid (aspen), which showed that increased GA levels could lead to enhanced xylem differentiation and cell elongation [46]. As a comparison, we investigated the xylem development of the hypocotyl of the *Arabidopsis* lines separately overexpressing *EguGA20ox1*, *EguGA20ox2*, and *EguGA2ox1* (Figure 9B). The results showed, similar to those in *Eucalyptus* (Figure 9C), that the *EguGA20ox1*- and *EguGA20ox2*-OE *Arabidopsis* lines exhibited a high level of xylem differentiation; this was reflected by the significantly increased xylem-to-stele ratio (Figure 9D). The *della* mutant also showed significantly improved xylem development, whereas the *EguGA2ox1*-OE line showed no difference from Col-0 (Figure 9D). Conclusively, these results suggested that overexpression of *EguGA20ox1* could improve xylem differentiation in *Eucalyptus*.

## 3. Discussion

Gibberellins play a pivotal role in regulating diverse aspects of plant growth, development, and responses to environmental stimuli, including seed germination, hypocotyl and stem elongation, wood formation, flowering, and stress adaptations [47,48]. The homeostasis of GA signaling is strictly regulated by various steps in GA biosynthesis, degradation, or signaling, most of which are controlled by a group of gene families, (*GA20ox*, *GA3ox*, and especially *GA2ox*) [43]. The temporal and spatial expression of these family genes in different plant organs, and in response to diverse environmental stimuli, lead to a more precise manipulation of GA homeostasis; this is very important for maintaining the adequate balance between fast growth and stress adaptions in plants. In *Eucalyptus*, one of the fastest growing woody plants, the roles of GA in regulating the abovementioned biological processes are poorly understood. Thus, identification and functional characterization of GA synthesis, degradation, and signaling pathways could further facilitate the molecular breeding of *Eucalyptus*. In this study, based on the third-generation transcriptome sequencing strategy PacBio Iso-Seq, we first identified the potential genes involved in GA synthesis, degradation, and signaling in the *Eucalyptus* hybrid *E. grandis* × *E. urophylla*, investigated the expression pattern of GA biosynthesis and degradation genes in response to abiotic stresses, and functionally characterized a few genes related to GA synthesis and degradation in *Arabidopsis*.

Gibberellin is one of the major signals that both regulates vegetative growth and controls the vegetative to reproductive transition in plants [43]. It positively regulates flowering in *Arabidopsis* [49], though it inhibits flowering in several woody plants, such as *Jatropha curcas* [50] and *Prunus persica* [51]. Overexpression of either *EguGA20ox1* or *EguGA20ox2* significantly shortened the flowering time, while *EguGA2ox1* delayed flowering in *Arabidopsis* (Figure 6), suggesting a conserved role of these genes from *Eucalyptus* in the regulation of flowering. Countless studies have demonstrated that early flowering is frequently accompanied by decreased vegetative growth. Early flowering caused a significant reduction in biomass accumulation by overexpressing *CO* and *FT* in *Arabidopsis* [52,53]. Our results showed that although the *EguGA20ox1*- and *EguGA20ox2*-OE lines both exhibited early flowering in *Arabidopsis*, they also promoted vegetative growth, resulting in significantly larger rosette leaves and increased numbers of leaves and biomass accumulation under normal growth conditions (Figure 6). The promotive effects of overexpressing *GA20ox* in vegetative growth were also reported in a few studies in tobacco and *Arabidopsis* [54,55,56,57]. In contrast, overexpression of *EguGA2ox1* caused a significant dwarf phenotype and was accompanied by decreased biomass and a dark-leaf phenotype, which is similar to that in rice [58,59,60]. As a comparison, we also analyzed the phenotype of the quintuple DELLA *Arabidopsis* mutant (*della*). The *della* mutant exhibited a significantly early flowering phenotype but had no growth-promoting phenotypes, as seen in the *EguGA20ox*-OE plants (Figure 6). In trees, high doses of GA might be toxic and can cause abnormal growth and cell death, as found in our previous study that showed different doses of GA in controlling shoot branching in *J. curcas* [61]. The diverse vegetative growth status of *GA20ox* OE and *della* mutants suggested that GA-regulated plant growth might be dosage-dependent, and that a moderate increase in bioactive GA or GA signaling in plants could improve vegetative growth.

Plants have developed elaborate strategies by spatially and temporally regulating the endogenous hormonal homeostasis to facilitate plant growth or adaptation to complex environments [27]. Phytohormones, such as abscisic acid (ABA), ethylene, jasmonic acid (JA), and salicylic acid (SA), are crucial factors in controlling plant adaptations to environmental stresses [62,63]. In recent years, an increasing number of studies have demonstrated that GA synthesis and degradation are tightly regulated in response to abiotic stresses [27,35,64,65,66,67]. In this study, more than half of the GA metabolism-related genes in *Eucalyptus* were significantly affected by salt or drought treatment (Figure 5). In *Arabidopsis*, stresses positively regulate the expression of GA degradation-related genes, while downregulating GA synthesis-related genes [21,37]. Moreover, overexpression of *GA2ox* confers abiotic stress tolerance in *Arabidopsis*, rice, and switchgrass [35,65,68,69]. However, the expression of *GA20ox2* can be induced by flooding stress in rice; this could promote stem elongation and enhance the flooding stress resistance [64]. In *Eucalyptus*, our results not only showed that the expression pattern of the key GA metabolism-related genes is closely correlated with stress responses, but further evidence in *Arabidopsis* showed that the transgenic *EguGA2ox1*-OE plants exhibited enhanced tolerance to abiotic stress (Figure 5 and Figure 6). On the contrary, *EguGA20ox*-OE plants were hypersensitive to salt stress and exhibited a significant decrease of the biomass accumulation by salt treatment. The significantly increased anthocyanin content in the leaf as a result of overexpressing *EguGA2ox* might contribute to the enhanced abiotic stress tolerance (Figure 7E). The GA-DELLA signaling pathway is central in regulating the anthocyanin synthesis in plants [70,71]. Thus, GA depletion by overexpressing GA degradation genes might enhance DELLA-mediated anthocyanin production, which not only promotes the abiotic stress tolerance, but also prevents chlorophyll degradation (Figure 7D) and the maintenance of photosynthetic capacity (Figure 7C), as previously reported [45]. Conclusively, our results agree with those in found in *Arabidopsis* and rice [37,65], which might suggest a common negative role of GAs in the regulation of abiotic stress adaptation. However, the molecular mechanisms of GA in the regulation of abiotic stress adaptations need further investigation in *Eucalyptus*.

Root morphogenesis and architecture are mainly controlled by auxin, cytokinin, and ethylene [40]. In recent years, more discoveries have shown that strigolactones also directly control root morphogenesis in response to environmental stimuli, mainly phosphate and nitrogen levels [30]. However, the role of GA in the regulation of root architecture is poorly understood. Here, we discovered that overexpression of *EguGA20ox* promoted the lateral root growth in both *Arabidopsis* and *Eucalyptus* (Figure 8). In *Populus*, overexpression of *GA2ox1* seems to increase the lateral root (LR) number and LR length [38,55], suggesting a negative role of GA in the regulation of root growth. Nevertheless, a recent report examining tobacco and *Melia azedarach* showed that ectopic expression of the *GA20ox* gene driven by a root preferential promoter significantly promoted root growth and biomass accumulation [55]. Here, we discovered that in both *Arabidopsis* and *Eucalyptus*, overexpression of *EguGA20ox1* could increase LR growth (Figure 8). These results might suggest the diverse roles of GA in regulating LR growth and development across different species. Intriguingly, we also discovered that in *Eucalyptus* seedlings inoculated with K599 (*EguGA20ox1*), transgenic hairy roots can be efficiently induced within days; hairy root induction typically takes a much longer time (at least three-four weeks), suggesting that GA might be a positive regulator in controlling root formation and development. Moreover, many GA metabolism-related genes exhibited high expression levels in *Eucalyptus* roots, especially early synthesis genes (Figure 4), which suggests that *Eucalyptus* root could be a potential place for the synthesis of GA or GA intermediates. Most previous studies of GA have focused on plant aerial organ growth and development, such as the elongation of the hypocotyl, stem, and leaves, and the regulation of seed germination and flowering. As we showed in this study, GA also directly participates in root growth in *Eucalyptus*, which should be highlighted in future research on perennial woody plants.

The role of GA and its interactions with other phytohormones in the regulation of xylem development have received considerable attention in recent years. However, wood formation in perennial woody plants seems to be more complex and lacks sufficient molecular and genetic evidence due to the lack of a genetic transformation strategy in most woody plants. In our study, we first obtained transgenic *Eucalyptus* roots overexpressing *EguGA20ox1* based on a modified transformation strategy, as reported previously [72], and investigated its role in the regulation of *Eucalyptus* root growth, including xylem development. The results showed a conserved role of GA synthesis genes in the regulation of xylem development, as reported in *Populus* and *Arabidopsis* [73,74]. Importantly, this system in *Eucalyptus* might be a convenient strategy for further investigation of the role of GA in the regulation of diverse aspects of root development and may facilitate the high-throughput selection and functional characterization of candidate genes in *Eucalyptus*.

## 4. Materials and Methods

### 4.1. Plant Materials and Growth Conditions

*E. grandis* × *E. urophylla* hybrid “Guanglin 9” clonal seedlings (three months after transplanting in the soil) were provided by the Guangxi Academy of Forestry (Nanning, China). *E. grandis* seeds were provided by the Guangxi Dongmen Forestry Farm (Chongzuo, China). The hybrid clonal seedlings and *E. grandis* seedlings were all planted in a growth chamber (25 °C, 100 μmol·m^−2^·s^−1^, 16-h light/8-h dark photoperiod) and fertilized every week with 1/2 Hoagland solution. Wild-type *Arabidopsis* Col-0 was used for genetic transformation. *Arabidopsis* plants were planted in a growth chamber (22 °C, 16-h light/8-h dark photoperiod). The *Arabidopsis della* quinary mutant (*gai*; *rga*; *rgl1*; *rgl2*; *rgl3*, CS16298) was ordered from the Nottingham Arabidopsis Stock Centre.

### 4.2. RNA Extraction, cDNA Synthesis and Quantitative Real-Time PCR

Total RNA of *Arabidopsis* and *Eucalyptus* was extracted using the Plant Total RNA Extraction Kit (Omega, Shanghai, China). The quantity and quality of RNA was verified by using gel electrophoresis and a Nanodrop 2000c ultraviolet spectrophotometer (Eppendorf, Framingham, MA, USA). The purified RNA was reverse transcribed into cDNA using the HiScript III 1st Strand cDNA Synthesis Kit (+gDNA wiper) (Vazyme, Nanjing, China). The quality of the cDNA was verified by amplification of the reference genes *ACT2* and *PP2A* from *Arabidopsis* and *Eucalyptus* using PCR. The relative gene expression level was quantified by qPCR using SYBR Master Mix (Vazyme, Nanjing, China) according to the manufacturer’s protocol on a LightCycler 96 system (Roche, Basel, Switzerland). PCR conditions were set as follows: 95 °C for 5 min, followed by 40 cycles of 95 °C for 15 s and 60 °C for 1 min. After amplification, a melting curve was obtained to confirm the presence of a simple amplicon. The relative gene expression level to the reference genes *Arabidopsis ACT2* or *Eucalyptus PP2A-1* and *PP2A-3* [75] was calculated based on the 2^−ΔΔCt^ method. The qPCR experiments were conducted in triplicate. Information on the primers is listed in Table S1.

### 4.3. Gene Cloning, Overexpression Vector Construction and Arabidopsis Transformation

The full-length sequences of *EguGA20ox1*, *EguGA20ox2*, and *EguGA2ox1* were amplified from the cDNA of *E. grandis* × *E. urophylla* using primers containing restriction sites and inserted into the pOCA30 expression vector after sequencing. The overexpression vectors *EguGA20ox1*, *EguGA20ox2*, and *EguGA2ox1* were introduced into the *Agrobacterium tumefaciens* strain *EHA105* and used for *Arabidopsis* transformation. Four-week-old *Arabidopsis thaliana* Columbia (Col-0) plants were transformed using *A. tumefaciens* and the floral dip method as previously reported [76]. More than ten independent transformants for each gene were obtained. The T2 homozygous *Arabidopsis* transgenic lines were used for further physiological and biochemical analysis.

### 4.4. Eucalyptus Transformation

*Eucalyptus* transformation to induce transgenic hairy roots was performed as previously described [72,77]. Briefly, *E. grandis* seeds were germinated in sterilized peat soil. One-week-old *Eucalyptus* seedlings were used for transformation. The seedling hypocotyls were infected with a needle swabbed with *Agrobacterium rhizogenes* (K599, OD600 = 2.0) by stabbing four holes along the hypocotyl. The infected seedlings were then transferred to a plastic box (20 cm × 10 cm × 5 cm) containing sterilized peat soil that was saturated with 1/2 Hoagland solution before use. Then, the plastic box was covered with a preservative film to maintain high humidity and kept under dim light conditions. One week after inoculation, the induction efficiency and the root phenotype were recorded. To calculate the transformation efficiency, at least three independent transformation experiments were performed.

### 4.5. Sample Collection, Iso-Seq Library Construction and Transcriptome Sequencing

Five different plant tissues from the three-month-old *E. grandis* × *E. urophylla* clonal seedlings were collected and frozen at −80 °C. The total RNA of the mixture of the five tissues was isolated following the aforementioned method. The quality of the RNA was further verified using Qubit and Agilent 2100. The Iso-Seq library was prepared following the isoform sequencing protocol of the Clontech SMARTer PCR cDNA Synthesis Kit and BluePippin Size Selection System, as described by Pacific Biosciences. Sequence data were processed using SMRTlink 5.0 software. Additional nucleotide errors in consensus reads were corrected using the Illumina RNA-seq data with LoRDEC v0.9 software. The consensus reads were then aligned to the reference using GMAP with the default parameters. The functional annotation of the unmapped transcripts and novel gene transcripts was based on the following databases: NR (NCBI nonredundant protein sequences), NT (NCBI nonredundant nucleotide sequences), Pfam (Protein family), KOG/COG (Clusters of Orthologous Groups of proteins), Swiss-Prot (a manually annotated and reviewed protein sequence database), KO (KEGG Ortholog database), and GO (Gene Ontology).

### 4.6. Sequence Analysis of GA Synthesis, Degradation and Signaling Genes

To identify the GA synthesis, degradation, and signaling genes in the *E. grandis × E. urophylla*, *A. thaliana*, *Oryza sativa*, and *Populus trichocarpa* genome, BLASTP with default parameters of the data files of different species was performed based on the conserved amino acid sequences using the amino acid sequences of *Arabidopsis* GA synthesis, degradation, and signaling-related genes as queries on the TBtools v1.115 software [44]. The search results were aligned by the ClustalW algorithm with default parameters to reduce duplicate and redundant sequences. The amino acid sequences of all GA-related proteins of *Arabidopsis*, rice, and *Populus* used in this study were downloaded from the NCBI database. The phylogenetic trees were constructed using the neighbor-joining method in MEGA (version 5.1). Unless specifically mentioned, the default parameters were used for all bioinformatic analyses. All the detailed information of the used sequences are listed as the Table S2.

### 4.7. Salt and Osmotic Stress Treatment of Eucalyptus and Arabidopsis

Three-month-old *E. grandis* × *E. urophylla* clonal seedlings with similar growth status were used separately for salt and drought treatments using 200 mM NaCl and 300 mM mannitol (to mimic drought stress), respectively. The growth pots were directly submerged in different solutions until the soil was completely saturated. After that, the plants were kept under normal growth conditions. Then, different tissues of the seedlings were collected for gene expression analysis. For the analysis of the stress response of different *Arabidopsis* lines, the same approaches were carried out on the three-week-old *Arabidopsis* seedlings. The survival rate, growth status, and other physiological parameters were recorded after stress treatment. For all the treatments, the experiments were conducted in at least three replicates.

### 4.8. Analysis of the Photosynthesis Parameters of Arabidopsis Seedlings after Stress Treatment

Light responses were monitored by simultaneously obtaining the chlorophyll fluorescence and other photosynthetic parameters in the leaves by using the Imaging-PAM M-Series Chlorophyll Fluorometer (Heinz Walz, Effeltrich, Germany). To investigate whether abiotic stresses could have diverse effects on photosynthetic activities, three-week-old transgenic *Arabidopsis* seedlings (*EguGA20ox1*-, *EguGA20ox2*-, and *EguGA2ox1*-OE lines, Col-0, and *della* mutant) subjected to salt stress for one week were used for analysis. Three mature leaves per plant (>3 plants per line) were selected for the parameter detection.

### 4.9. Paraffin Section and Microscopic Observation

Hypocotyl and root cross-sections of *Arabidopsis* and *Eucalyptus* plants were collected and histologically analyzed. Briefly, the plant samples were fixed in FAA solution and then dehydrated in an increasing concentration of ethanol from 30% to 100%. After hyalinization in xylene, the samples were embedded in paraffin. Then, the samples were cut into slices using a microtome. After dewaxing, the samples were double stained with 1% sarranine and 0.5% fast green. Images of stem sections were used to measure the xylem diameter using ImageJ v1.54d software.

### 4.10. Statistical Analysis

Multiple comparisons between different groups were carried out using R (version 4.2.2). The data were analyzed using one-way ANOVA and Duncan’s test, with significance set at *p* < 0.05. Student’s t test was also used to analyze the significant differences between the indicated groups and the control. The heatmaps were constructed using the pheatmap package (1.0.12).

## 5. Conclusions

In this study, the regulatory gene network of gibberellin biosynthesis, degradation, and signaling was comprehensively analyzed, based on the transcriptome of five major vegetative organs of *E. grandis* × *E.urophylla*. Our results showed that the genes involved in GA synthesis and degradation were diversely regulated in different vegetative organs and in response to the biotic stresses in *Eucalyptus*. the expression of most *GA2ox* genes was positively regulated in response to stress treatment. Importantly, the functional characterization of GA synthesis genes *EguGA20ox1* and *EguGA20ox2*, and GA degradation gene *EguGA2ox1* in *Eucalyptus* and *Arabidopsis*, demonstrated that they participated in the regulation of plant growth, root initiation and growth, abiotic responses, and xylem development. Our study provides the first comprehensive understanding of regulatory pathways of GA metabolism and signaling, and demonstrated the key role of GA in regulating the balance between plant growth and stress responses. This could further benefit molecular breeding for obtaining high-yield and stress-resistant *Eucalyptus* cultivars. 

## Data Availability

The raw data of the transcriptome is available in Genbank with the accession numberPRJNA944269.

## Supplementary Materials

The supporting information can be downloaded at: https://www.mdpi.com/article/10.3390/ijms24087051/s1.
