# Supplementary material for: Transcriptome-Based Construction of the Gibberellin Metabolism and Signaling Pathways in Eucalyptus grandis × E. urophylla, and Functional Characterization of GA20ox and GA2ox in Regulating Plant Development and Abiotic Stress Adaptations
Source: Int J Mol Sci. 2023 Apr 11;24(8):7051. doi: 10.3390/ijms24087051 (PMC10138970; doi:10.3390/ijms24087051)
Supplement: Supplementary file 1 [file ijms-24-07051-s001.zip › Figure S1, S2, and Table S1.pdf]

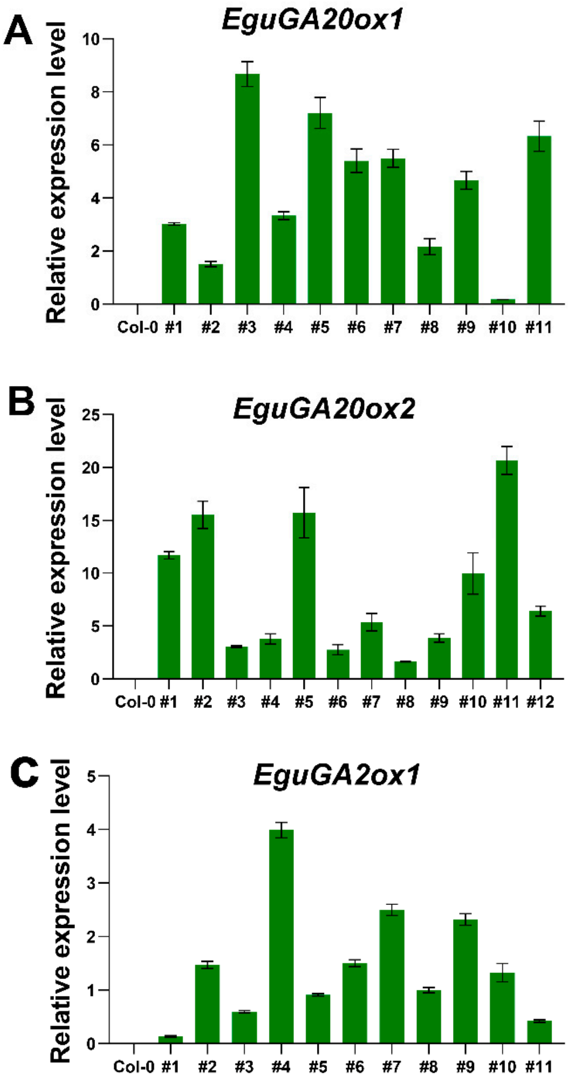

Figure S1. Gene expression of different transgenic *Arabidopsis* lines overexpressing *EguGA20ox1*, *EguGA20ox2* and *EguGA2ox1*. Eleven *EguGA20ox1* OE lines (A), twelve *EguGA20ox2* lines (B), and eleven *EguGA2ox1* lines (C) were used for the gene expression analysis by RT qPCR (n = 3). The data represents a relative expression of the target gene to the reference *ACT2*. Data represents as mean ± sd.

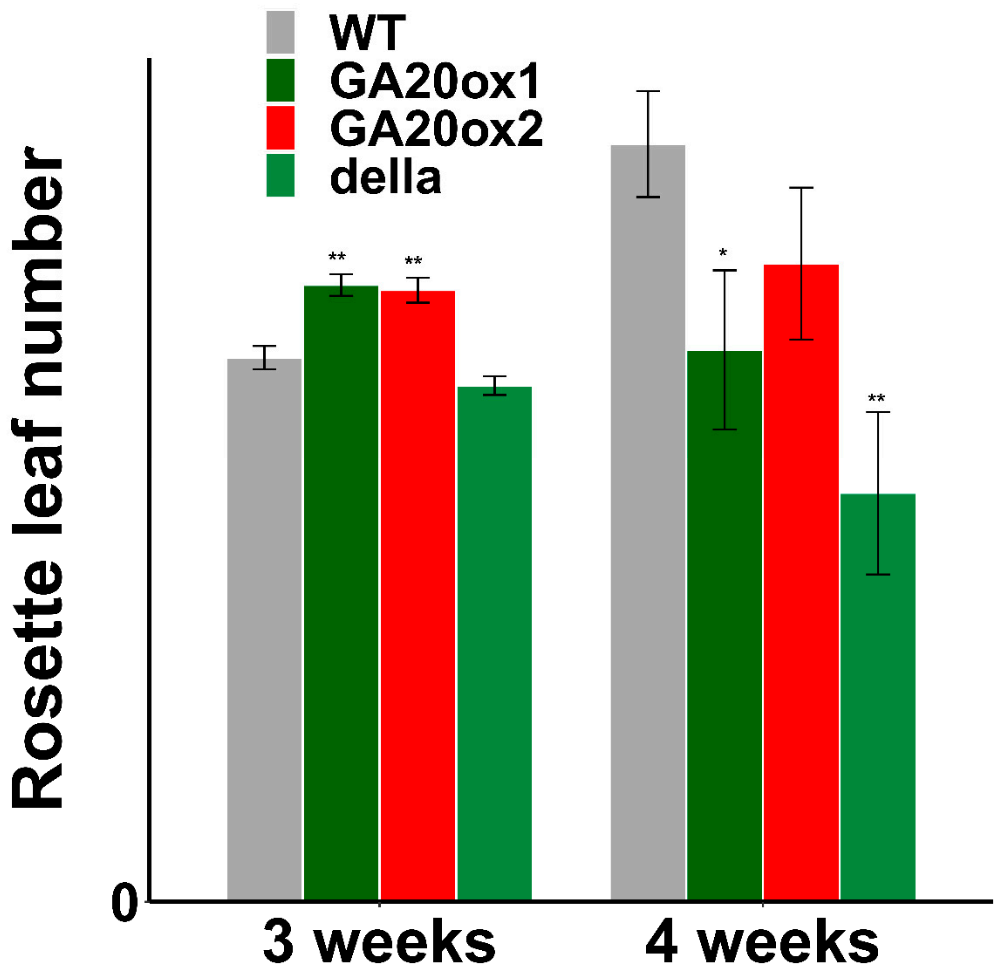

Figure S2. Number of the rosette leaves before and after bolting of different *Arabidopsis lines* (n = 20). Data represents as mean ± sd. *, $p < 0.05$, **, $p < 0.01$.

Table S1. Information of the primers used in this study.

Gene	Primer name	Primer sequence	Application
EguGA20ox1	Forward	TGAATGCAATGGCAGTTGATTGCCT	Overexpression
	Reverse	AAATCTTCATCACCGAACTGGTTCA	
EguGA20ox2	Forward	ATGTCTTTCTTATTGGACACAAGAA	Overexpression
	Reverse	CTAGAGAGACATTTAGGCTTTGGGA	
EguGA2ox1	Forward	GATGGTTGTCCCGTCCAAACTAG	Overexpression
	Reverse	CGACATCAAGAAGCGGCTACTCT	
EguCPS	Forward	GCAGTCACTGGAATGTTCAACCTCTATA	qPCR
	Reverse	TCGCCGCCATACTGGTCTATGTAA	
EguKS1	Forward	TGACAGCAGCATACGCCGATTG	qPCR
	Reverse	ACAAGAACAAGCATCGATCACCAACTA	
EguKS2	Forward	TATTGTTCCTCTCTTTGTTCTCACCTCG	qPCR
	Reverse	GCCGCCATATGCTCTTACATCCC	
EguKO1	Forward	GATCGTATCCCAGCACTTGCAGC	qPCR
	Reverse	ATCACCGGCCCGTACTTCTTGG	
EguKO2	Forward	ACGGCATGGAGGAGCTGAACA	qPCR
	Reverse	GGCAAGGAGTTACACCACATTCGTTA	
EguKAO1	Forward	GATTCCTTCATGTCCTCCTTCGTCAC	qPCR
	Reverse	AGTGGCAGTCAAACGCCTAAGC	
EguKAO2	Forward	GCCAAGGTCATTGACGAAACACTTC	qPCR
	Reverse	CCGCTCTGGTATGTAATTATCCATCTGG	
EguKAO3	Forward	GTTGATTGTGACCATGCCTGAGCC	qPCR
	Reverse	CCATCTTTGACCACTTATCAAGTGCAGA	
EguGA20ox1	Forward	GCCTCAAGAGCTTGTAGCTTCCTC	qPCR
	Reverse	GTAGTGGTAAAGAGCACGGTGAGTTC	
EguGA20ox2	Forward	CCAGAAGTATTGTGAAGCGATGAAGGA	qPCR
	Reverse	TGGTGGAGGATGGTCAAGGATGTC	
EguGA20ox3	Forward	CCTATCGCCCATTTCTTTCGTGACA	qPCR
	Reverse	TAGCAGCTCCATTATTCCTAGAGACAGT	
EguGA20ox4	Forward	TCTCGAGTTCACTCAGAAGCATTACAG	qPCR
	Reverse	TCTTCTTTGTCGTCGTCAATGTCATCT	
EguGA2ox1	Forward	CTGGAGGAGGAAGCCGTCAAGT	qPCR
	Reverse	CACCGCCGATGTGTAATCACTCAA	
EguGA2ox2	Forward	CTCCGCATCATACCATCATCATTGTCT	qPCR
	Reverse	GACCACCTTGAAGAACCCGAACTC	
EguGA2ox3	Forward	GACAAGCGTCTCGACCTCTTCAAG	qPCR
	Reverse	CAACAGTACACCAACACTAGGAGAGC	
EguGA2ox4	Forward	GAGGTTGAAGAGCGTGAGGCATC	qPCR
	Reverse	TTGAAGAGGTCGAGGCGAGAGTC	
EguGA2ox5	Forward	GGTCATCAGTTGTGGGAGCAAAGT	qPCR
	Reverse	TGGTTGGCAGCGAGGATGGT	
EguGA2ox6	Forward	CTGTTAAACCTAATCCCGATGCTCTCAT	qPCR

	Reverse	CATCTTCTTGGACCTGTCGTCTGAATT	
EguGA2ox7	Forward<br>Reverse	ACGTGCTTCCTCCGGCTGAA<br>GCCTGAAGAAGGTCTCCGATGTTG	qPCR
EguGA2ox8	Forward<br>Reverse	TGGCTGAGCAGTGCGTGTGA<br>CGGCGGTAGGAGTTGTTGAGGA	qPCR
EguGA2ox9	Forward<br>Reverse	AGAAAGCCCTCCGTCTACAGAAAGT<br>GCCTCAGCATCTCCTCGTCCAT	qPCR
EguGA2ox10	Forward<br>Reverse	GGAGAGGTTCTGGCCGTTGC<br>TAGACGGAGGGCTTTCTGCAGC	qPCR
EguGA2ox11	Forward<br>Reverse	AGAGAGGTTCTGGCCGTTGCC<br>AAGACGGAGGGCTTTCTGCAGAA	qPCR
EguGA2ox12	Forward<br>Reverse	GACCTTAGACTTGATCCAGCACACTC<br>ACTTGTATCCATTGCTCGTCCTTGTAG	qPCR
EguGA2ox13	Forward<br>Reverse	TAGGCGATATGTTGCAGCTATGAGC<br>TGAACCCAGGGTCCTGACGTTT	qPCR
EguGA3ox1	Forward<br>Reverse	TCTCGTTCCCCAGAAGGCATCTC<br>TCTTCCTTTGTCAACCCCAAAGAGC	qPCR
EguPP2A-1	Forward<br>Reverse	AAGGAACTTGAAGAACCAGATGGAAGG<br>AGAACCTTGTGCCAATGATGCTCTC	qPCR
EguPP2AA3	Forward<br>Reverse	GAGGTCCGCTGCATCGAATCTG<br>CGCACTCGCCAAGACTTATCCTG	qPCR

Table S2. Gene ID and sequences of the genes used in this study (Supplied as a single document).
