# Supplementary material for: Transcriptome-Based Construction of the Gibberellin Metabolism and Signaling Pathways in Eucalyptus grandis × E. urophylla, and Functional Characterization of GA20ox and GA2ox in Regulating Plant Development and Abiotic Stress Adaptations
Source: Int J Mol Sci. 2023 Apr 11;24(8):7051. doi: 10.3390/ijms24087051 (PMC10138970; doi:10.3390/ijms24087051)
Supplement: Supplementary file 1 [file ijms-24-07051-s001.zip › Table S2 Gene ID and sequences of the genes used in this study.pdf]

Table S2. Sequence information of the sequences used in this study.

1、 Eucalyptus grandis x Eucalyptus urophylla

>*EguCPS*　m64168e_220610_021218/64553640/ccs

GGGATTATACCTCCTCATTCCCCATCAAGCAACAGCAGCAGCAGCAGGCCGCGACATCGCACGCACGCGACAGCACCAGC
TCCGACTCTAGCGTCAACCGCACGCGAACATGTCCTCCATCGCTTCCTTCCTCTCTCCGCTCCCGCTCCCCCCGCGACGT
GTCGCTCGCCCTCCATTCTCGGCCAGCGTCGGTACTCCCCCGCCAAGCCTTCCCAGCTTCTGGGTGTTTTGCCTTCCCCG
GATAACGACAAGCGTCATCTTCGCTCCGATATTCGCCCCAGATGTAGCGCCATATCCAAACCTCTCACCCAAGAGTATGA
TGTGCTTCAGAATGGTCTGCCAATTATCAAGTGGCGAGAGATTGTGGAGGATGACTTAGGAGTTGAAGGTGTTAAGGTTG
CTCATCCATCAAGTGGGATCACCAAACGTGTCGAAGCAACCAAGTCGATGTTGGGGTCGATGACCGATGGAGAAATAAGT
ATATCGGCCTATGATACGGCCTGGGTTGCTCTTGTAGAGGACGTCAGCGGGAGCGGTAGCCCTCAGTTCCCTGAAGCTCT
ACGGTGGATCGTGGACAATCAGCTTCCCGACGGTTCTTGGGGCGACGATCTCATCTTCTCCCCTCATGATCGAATCATCA
ACACCTTGGCTTGTGTCGTGGCTCTGAAGTACTGGAAGATTCATCCTGACAAATGCGAAAAAGGAATGACGTTCTTCAGA
GACAACATATCTAAACTTGGAGAGGAGGACTCCGAGCACATGCCCATCGGGTTCGAAGTTGCTTTTCCTTCGCTTATCGA
GACCGCTCGGAAATTGGAGATCAAGATATGTGACGACTCTCCCGTCTTGCAAGAGATATACGCGAAGAGGAATCTGAAGC
TCAACAAGATACCAAAGGACATAATGCACCAAGTGCCCACAACTCTACTCCATAGCTTGGAGGGGATGGAGGGCTTAAAT
TGGGAAAAGCTCCTGAAACTGCAGAGCGCAGATGGATCGTTCTTGTTCTCTCCCTCCTCCACTGCATTTGCACTCATGCA
AACCAAGGATGCTAATTGCTTGGATTATTTAAGCAGAGCTGTCCAAAGATTTAACGGAGGAGTTCCAAATGTGTATCCGG
TTGACTTGTTCGAACATCTGTGGGCCGTTGACCGCTTGCAGCGACTCGGAGTCTCTAGGTACTTCAAGGATGAGATCAAA
GGATGCATGAGCTACGTTCAAAGATATTGGAGTGAAAAAGGGATCTGCTGGGCGAGAAATTCAAGAATCAGTGATATTGA
CGACACTGCCATGGGATTCAGGCTCTTGAGACTCCATGGCCACGAAGTGTCTGCTGACGTGTTTGAACAATTTAAGCGCG
GGGATGTGTTCTCAACCTTCATGGGGCAGTCGACGGAGGCAGTCACTGGAATGTTCAACCTCTATAGGGCCTCCGAGTTG
ATCTTTCCAGGCGAGAAAATCCTCGAGGATGCGAAAAGCCATGCAGTGAAGTTTCTGAGACGGAAACGAGAAGCTAACGA
GCTACTTGATAAGTGGATCAGAACCAAGGACTTACCTGGCGAGGTCGGGTACGCTCTGGATGTTCCATGGTATGCAAGCT
TACCTCGTGTGGAGACAAGATTTTACATAGACCAGTATGGCGGCGAAAACGACGTTTGGATTGGCAAGACTCTCTACAGG
ATGGGGTACGTTAACAACAATGTCTATCTCGAGCTCGCAAAGCTGGATTACAACAATTGCCAAGCCTTGCACCTCTCGGA
ATGGGACAATTTTCAAGGTGGTATTGCGAGAGCAAACTTGTGGACTTCGGGACAAGTGGGAAGACTCTCCTTTATTCTTA
CTTTACGGCAGCAGCAAGCATTTACGAACCGGAACGGGCGAGAGAGAGACTCGCGTGGGCCAAAACGAGTGTGTTGGTGG
ATGCGATCGCGTCGTACTTGGAGGGAGAGGGAACCTCTCGTGAGCGAAGAAGAGGCTTCGTTCGTGAGTTCCAGCAATTC
TCCAAGAAACAGAAGCACATAAACGGAAGCAGGAGTCCATTCCCAGCAAGGGAGGACAAGGGCTCATTACAGCTCTGCTT
GGAACCCTAGACCAACTTTCATTGGAAGTCTTGGTGGCTCGTGGTAAAGACATTGGCCATGCTTGCGTACTGCGGTAAAG
CCTATATTCCACTAATTAAATTGAATACAGTTGTTACAATGTATAACCACATTATGATCTTTCATTAGGAAAAGAAAAGA
CAGGGTAACCAT

>*EguKS1*　m64168e_220610_021218/25756518/ccs

GGGGTTTCTCTCTTTGTTCTCACCTCGGCTCTCACAAATTCCAGCAGTCTCACAGGTTCTCTGTCTTCCGTCTCGTGTGA
ACAGCTCGTTGTTGAAAATGCTTCACCTACTTGTGATATCCCCCCCCGCGACTGGTTCTCGTGAGAAATGATGGTATATA
TAGGACGGCCAAAAGGGTCTGCAAATTTTAACGGCCCTGAAATATGGGATGCGTTTGCTGGGGGTTGTAAGAGCATATTG
CGGCTCACTAGATGGAACCCTGCTGCAAAAGAATGAAGATTCCCTTGAACATCAAGCTTTGTCATCAAGCTCTTGTTGCT
TATGCCAACTACATTCGATGGCATGGTTGATCGTCTGTCTTCTCGTCCTCATGGGTTTTGAGATTAACTTGCATTCGAGT
TGGAGCTGAGGAGAAACTAGCGGATAAAGTGGTGCTGCTGATTTTCTCATACTGTTCTACCATGGGACACTCACATTTCT
CTATTCGTGGGTTGTCTGCTCCGATATCAGGAGCATTTCCTTAGACTTTGGATTAATATCTGTTAAACAATCATCATCAT
GTGTTGCCGTTGCGTTGGGCGAAGAAGTAAAGAACGATTAGTGATATGTTTAACAGGTTGAACTCTCTCTTTCTTCATAT
GACACTGCTTGGGTGGCTATGGCCCTTCGCCTTGTTCTCCACATAAGCCCCCTTTTCCCTCATGTGTCCTTGCACTTAAG
CATTGGGGTGTTGGTGAAAGGCAGAAAAACAAAGGGCCTTGAATTTATTGCATCGAACTCTGCTTCTGTAATCGATGACA
GCAGCATACGCCGATTGGATTCAATATTTTGCTTTCTGGCTGATTGAGCAGCTGATTGCCTAAATTAAACCTACCATGAG

AACCTGCAGTTTGATTCTGTGTCTTACAAAGAATTTTGGAAGTGAAGAGGAAAAATATTGGTGATCTTCCCGTTTTGGTT
TTCTTCAATTAACCAATGTGCAAATCATGAACTAAGTTGTTCTTATAGTTGGTGATCGATGCTTGTTCTTGTATTTCCTT
GAATATTTACTGCACAAAACTTATATGCTATAGTGAAAGCAGTATATGAACTTCATTTATAGGATCAGTGAATTCTGTGT
GCTTTGTCTCTTAGAGCTTTCCGGGATCAGTGGAAGATACTTGTCTTATGTTGCAGAAGGAATGGGAAGTTCTGCAGATG
GAAATGATTATGAAGTATCAACGGAAAAAATGGGTCACTATTCAATTCACCATCAACAAGCTGCTGCTCTAACTCACCTT
CAAAATGCAGTTGTCTACTCTAACCTTGAATCGGTATAGAGATTTTGGTGATGAAGTTCCAACAATCTATCTTTGGAAAT
ATAGCTCGTCTATGTATATAGGAATTTGAGAGATTGGCATTATCGATATTTCAGAAGGGAAGTAAATTCATGTATTGGAC
GACACGTACAGATGCTGGTGCAGGGCGAAGAAGAGATTTTTCTGGACATTGCCACCACCGCAATGGCATTTAGGATGCTG
CGATCCCATGGATATGATGTCTCTTCAGATGCATTAAATCAATATGCTGAAGAGGATCAATTTTGCAATACACTTGAAGG
ATATGTGAAGGATGCTGGCTCTGTCTTGGAATTGTATAGGGCTTCACAGCTGATCATCAATGATGATGAAATTATTCTGG
ATAAAATCAACTCTTGGACCTATGATTTTCTTAGAATAGGTTTAACACACTGGTAAATATGCATGCCAATAGATTGAGAG
TTATATATGTGGAGAGGTGGGTGAATGCACTTAAATTTCCTCTCCATGCAAACTTGGAAAGGGTAGCAAACAGGAGAAAT
ATAGAGCTCCTACAACATAGACAGTACAAGAGTTCTAAATGTGGTCTCCGTCCTGTAACTTTTGCAACACAAGATTTCCT
GAATCTGGCAGTGAATGACATAACAACTGTCAAGCAATATACCAAGAAGAACTCAAATATCTGGAAAGATGGTCAAGAGA
AAAGGTTAGACAACTGAAATTTGCGCGACAAGAAGCTGGCATATTGTTACTTTTCTGCTGCAGCAAGCTTCTCGCCACCT
CAATTATCAGAGGCCCGCATTTCTTGGGCCAAAAATTGGTGTCCTCACTACAGTGGTTGACGACTTCTTGATGTTGGTGG
CTCTGCGGAGGAGCTTGAAAATTTTATATGGTGGTTAAAAGGTGGAATGTAAACTTGAGTGCTGACTGCTGTTCTGACGC
AAGTTCAAATCATATTTTCAGCACTTCACTCTACTATTTCTGAGATTGGAGACAAAGCAGTGACATGGCAAGGAAGAAAT
GTGACGGGTCATGTGGCTCAAATTTGGTTGAATTGCTCGAGTCTATGCTGACTGAGGCCAAGTGGACTAGAAAGAAGGCA
GTGCGACGATGGATGAATACATGGCAAATGCATACGTCTCATTTGCCTTTTTGGGGGACCTATTGTTCTCCCAGCTCTCT
ACCTTGTTGGTCCTAAACTTTCGGAGGAGCAAGTTGAAAGCCTTGAGTACCGAAAGTTATTCAAGCTGATGAGCACTTGT
GGGCGCCTTCTAAATGACATGCAGGGTTTCAAGAGGGAATCCAGGGAAGGGAAGTTGAATGCGGTGACATTGCAGATGCT
TGACAGGAGCAGCACAGTGAAAGGGAGGCCACAGAGAGGATGAGCAGTATTATCATCAGTAAAAGGAGGGAACTCTTGAA
GTTGGTTTTGCAAGAAAGGGATAGCATAGTTCCAAGAGCGTGCAAGGACTTATTCTGGAAAATGAGCACTGTGCTGCACT
TCTTCTACATGGATGATGATGGCTTCACTTCAGATGAAAAAACTAGTGCTGTTAAAAGCTCTCCTTGATCAGCAATCACT
TCAATGAACTGGAATTGCTTGAGAGCTCGCGAGCAGAACTCCTATAGCACTGCTGTCTATGTCTGTGGGAACGTCGATCG
CACCTGTATATTGCATGACAATATTGTACTAACATTTCTGTGCCTAATGACGGAGGCGTGATC

>*EguKS2* transcript_HQ_hun_transcript20615/f3p0/2998

GGATATTGTTCCTCTCTTTGTTCTCACCTCGGCTCTCACAAATTCCAGCAGTCTCACAGGTTCTCTGTCTCTCCGTCTCG
TGTGAACAGCTCGTTAGTTGAAATGCTTCACCTACTTGTGATCTCCCCCCCCGCGACTGGTTCCTCGTGCAGAAATGATG
TATATATATAGGACGGCCAAAAGGGTCTGCAAATTTTAACTGCCCCTGAAATATGGGATGCGTTTTCTGGGGATGTAAGA
GCATATGGCGGCTCCACTAGATGGAACCCAGCTGCAAAAGAATGAAGATTCCCCTTGAACATCAAGCTTTGTCATCAAGC
TCTTTGTTGCTTATGCCCAACTACATTCGAGTGGCATGGTTGATCGTCTGTCTTCTCGTCCATCATGTGTTTTGAGATTA
ACTTGCATTCGAGTTGGAGCTGAGGAGAAACTAGCGGATAAAGCTGGTGCTGCTGATTATTCTCATACTGTTCTACCATG
GCACACTCACATTTCTCTATTCGTGGGTTGTCTGCTCCTATATCAGGAGCATTTCCTTTAGACTTTGGATTAAAATCAGT
TAAACAATCATCATCATGTGTTGCCGTTGCGTTGGGCGAAGGAAGTAAAGAACGAATTAGTGATATGTTTAACAAGGTTG
AACTCTCTCTTTCTTCATATGACACTGCTTGGGTGGCTATGGTCCCTTCGCCTTGTTCTCCACAAAGCCCCCTTTTCCCT
AGGTCTGTGAGTTGGTTGATGAATAATCAATTATGTGATGGTTCTTGGGGTCTCCCTGATCATCATCCATTATTAATCAA
GGATGCTTTGTTATCGACTTTAGCATGTGTCCTTGCACTTAAGCAATGGGGTGTTGGTGAAAGGCAGATAAACAAAGGCC
TTGAATATATTGCGTCGAACTCTGCTTCTGTAATCGATGACAGGCAGCATACGCCGATTGGATTCAATATTTTGCTTTCT
GGCATGATTGAGCAGGCTGATTGCCTAAATTTAAACCTACCCCTGAGACCTGCAGATGTAGATTCTGTGTCTTACAAGAG
AAATTTGGAAGTGAAGAGAGGCTTATCCGGGATCAGTGGAAGATACTTGTCTTATGTTGCGGAAGGAATGGGAAGTTCTG
CAGACTGGGAAATGATTATGAAGTATCAACGGAAAAATGGGTCACTATTCAATTCACCATCAACCACAGCTGCTGCTCTA
ACTCACCTTCAAAATGCCAGTTGTCTACACTACCTTGAATCGGTACTAGAGAAGTTTGGTGATGCAGTTCCAACAATCTA
TCCTTTGGAAATATATGCTCGTCTATGTATGATAGAGAATTTGGAGAGATTGGGCATTGATCGTTATTTCAGAAAGGAAG

TAATTCATGTATTGGACGACACGTACAGATGCTGGCTGCAGGGCGAAGAAGAGATTTTTCTGGACATTGCCACCACCGCA
ATGGCATTTAGGATGCTGCGATCCCATGGATATGATGTCTCTTCAGATGCATTAAATCAATATGCTGAAGAGGATCAATT
TTGCAATACACTTGAAGGATATGTGAAGGATGCTGGCTCTGTCTTGGAATTGTATAGGGCTTCACAGCTGATCATCAATG
ATGATGAAATTATTCTGGATAAAATCAACTCTTGGACCTATGATTTTCTTAGAAAAGGTTTACACACTGGTAAAATGCAT
GCCAATAGATTGGAGAGTTATATATGTGGAGAGGTGGATGATGCACTTAAATTTCCTCTCCATGCAAACTTGGAAAGGGT
AGCAAACAGGAGAAATATAGAGCTCTACAACATAGACAGTACAAGAGTTCTAAAATGTGGTCTCCGGTCCTGTAACTTTT
GCAACAAAGATTTCCTGAATCTGGCAGTGAATGACTTCAACAACTGTCAAGCAATATACCAAGAAGAACTCAAATATCTG
GAAAGATGGGTCAAAGAGAAAAGGTTAGACAAACTGAAATTTGCCCGACAGAAGCTGGCATATTGTTACTTTTCTGCTGC
AGCAAGCTTCTCGCCACCTCAATTATCAGAGGCCCGCATTTCTTGGGCCAAAAATGGTGTCCTCACTACAGTGGTTGACG
ACTTCTTTGATGTTGGTGGCTCCGCGGAGGAGCTTGAAAATTTTATATGGTTGGTTAAAAGGTGGAATGTAAACTTGAGT
GCTGACTGCTGTTCTGAGCAAGTTCAAATCATATTTTCAGCACTTCACTCTACTATTTCTGAGATTGGAGACAAAGCAGT
GACATGGCAAGGAAGAAATGTGACGGGTCATGTGTCTCAAATTTGGTTGGAATTGCTCGAGTCTATGCTGACTGAGGCCA
AGTGGACTAGAAAGAAGGCAGTGCCGACGATGGATGAATACATGGCAAATGCATACGTCTCATTTGCTTTGGGACCTATT
GTTCTCCCAGCTCTCTACCTTGTTGGTCCTAAACTTTCGGAGGAGCAAGTTGAAAGCCTCGAGTACCACAAGTTATTCAA
GCTGATGAGCACTTGTGGGCGCCTTCTGAATGACATGCAGGGTTTCAAGAGGGAATCCAGGGAAGGGAAGTTGAATGCGG
TGACATTGCAGATGCTTGACAGGAGCAGCACCAGTGAAAGGGAGGCCACAGAGAGGATCAGTAGTATTATCATCAGTAAA
AGGAGGGAACTCTTGAAGTTGGTTTTCCAAGAAAGGGATAGCATAGTTCCAAGAGCGTGCAAGGACTTATTCTGGAAAAT
GAGCACTGTGCTGCACTTCTTCTACATGGATGATGATGGCTTCACTTCAGATGAAAAAAACTAGTGCTGTTAAAGCTCTC
CTTGATCAGCCAATCACTCTCAATGAACTGAAATTGCCTTGAGAGCTCGCGAGCTCGCAAGCAGAACTCCTATGGCACTG
CTGTCTATGTCTGTGAGAATGTCGATTGCACCTGTATATTGCATGACAATATGTACTAACATTTCTGTGCCTAATGACGG
AGGCGTGATCATATCCTACTTATGCTTCTTGCTTTTCG

>*EguKO1* transcript_HQ_hun_transcript51409/f76p0/1823
GGGCCCGCGCTCTTCACTAAATATTGGATCGTATCCCAGCACTTGCAGCGCATAGATGGCGATTCTCGCCGACAAAGTCC
TGCTCCGAGAGATCGCCTCCGCGGCTCTCCTCTTCCTCATTACCCGATTCTTTGTCAGCTCTCTCCTGCTTCGCTTGAAC
TCCTTTAAGCTCCCGCCTGGCCCGAAAGGGTGGCCGGTCATCGGCGCCATCCCTCTCTTGGGGAACATGCCCCATGTAGC
GCTGGCCAAAATGGCCAAGAAGTACGGGCCGGTGATGCACCTGAAAATGGGCACGTGCGGCATGGTCGTGGCGTCGACCC
CTGACGCCGCCCGGGCTTTCTTGAAAACCCTAGACATCAACTTCTCCAATAGGCCCCCGAATGCGGGCGCGACCCACTTG
GCCTATGACGCGCAGGACATGGTCTTCGCTGACTATGGCCTGAGGTGGAAGTTACTTAGGAAGCTGAGCAATTTGCACAT
GCTCGGAGGGAAGGCCCTTGAGGACTGGGCTCGCGTCCGAACGTCCGAGCTAGGGTACATGCTTCAAGCCATGTGCGAGT
GCAGCAAGCGAGGCGAGCCGGTGGTGGTGCCGGAGATGTTGACCTATGCCATGGCGAACATGATAGGGCAAGTCATTTTG
AGCCGAAGGGTGTTCGTGACGAAGGGCTCCGAGTCTAACGAATTCAAGGACATGGTGGTAGAGCTGATGACAAGCGCAGG
GTTCTTTAACATCGGCGACTTCATCCCGTCAATTGCATGGATGGACTTACAAGGGATCGAAGGCGGAATGAAGCGCTTGC
ACAAGAAATTCGACGGGCTGTTGACGAAGATGATCGAGCAACACTCGGCAACTGCTCACGAGCGTAAAGGAAACCCGGAT
TTTCTCGATGTGGTCATGGCTAACCGAGATAACTCCGGGGACGAGAAGCTTAGTTTGACCAACATTAAGGCTCTTCTCTT
GAATTTGTTCACTGCGGGCACTGATACTTCATCGAGCATAATCGAGTGGTCGCTGGCCGAGATGTTGAAGAACCCCAGCA
TCCTCAAGCGAGCCCACGAAGAGATGGACCGAGTCATCGGGCGGCACCGACGCCTTGAGGAATCCGACCTCCCGAAGCTC
CCGTACTTGCAGGCCATATGCAAGGAGAGCTTCCGAAAACATCCATCGACACCGCTAAACCTACCGAGGGTCTCGACCGA
GGCGTGCCAAGTGAACGGCTACTACATCCCCAAGAACACGAGGCTGAGCGTGAACATATGGGCGATCGGGCGGGACCCGG
ACGTGTGGGAGAATCCTCTGGACTTCACCCCAGAGCGGTTCTTGAGTGGGAGGAACGCGAGGATCGACCCGCGCGGGAAC
GACTTTGAGCTGATCCCGTTCGGGGCAGGGCGGAGGATCTGCGCCGGGACGAGGATGGGGATCGTGCTGGTCGAGTACAT
ACTGGGGACTTTGGTCCACTCGTTCGACTGGAAGCTGCCGGACGGCGTGGAGGAGCTGAACATGGACGAGGCGTTCGGCC
TGGCGTTGCAAAAGGCCGTGCCCCTCTCGGCCGTCGTCACCCCACGGCTCTCCGTTAGCGCGTACGCACCTTAAAGCTTC
CGCAAAAAGCTTTTCCACTATGAGAATCCGACATTATCATGACACTCGGAGTTTTATTAGACTAGTGATGGGTGGTTCCC
CAGATGGGGCCTATGGCGAATGCGACGTAACTCCCTGCTCTTTCGGTGTATTCTCTTTTCTGTGTTTTCGAATAATCCAG
TGTCTTTGTGTGATTGTAATAATATGAACTTAGTGTTATATAATATACGTGTGTGCTTTGCTT

>*EguKO2* transcript_HQ_hun_transcript51487/f25p0/1801

GGGCCCGCGCTCTTCACTAAATATTAAATCGTATCCCAGCACTTGCAGCGCATAGATGGCGATTCTCGCCGACAAAGTGC
TGCTCCGAGAGATCGCCTCCGCGGCTCTCCTCTTCCTCATTACCCGATTCTTTGTCAGCTCTCTCCTGCTTCGCTTGAAC
TCCTTTAAGCTCCCGCCTGGCCCGAATGGGTGGCCGGTCATCGGCGCCATCCCTCTCTTGGGGAACATGCCCCATGTAGC
GCTGGCCAAAATGGCCAAGAAGTACGGGCCGGTGATGTACCTGAAAATGGGCACGTGCGGCATGGTCGTGGCGTCGACCC
CTGACGCCGCCCGGGCTTTCTTGAAAACCCTAGACATCAACTTCTCCAATAGGCCCCCGAATGCGGGCGCGACCCACTTG
GCCTATGACGCGCAGGACATGGTCTTCGCTGACTATGGCCCGAGGTGGAAGTTACTTAGGAAGCTGAGTAATTTGCACAT
GCTCGGAGGGAAGGCCCTCGAGGACTGGACTCGCGTCCGAACGTCCGAGCTAGGGTACATGCTTCAAGCCATGTGCGAGT
GCAGCAAGCGAGGCGAGCCGGTGGTGGTGCCGGAGATGTTGACCTATGCCATGGCGAACATGATAGGGCAAGTCATTTTG
AGCCGAAGGGTGTTCGTGACGAAGGGCTCCGAGTCTAACGAATTCAAAGACATGGTGGTAGAGCTAATGACAAGCGCAGG
GTTCTTTAACATCGGCGACTTCATCCCATCAATTGCGTGGATGGACTTACAAGGGATTGAAGGCGGAATGAAGCGCTTGC
ACAAGAAGTTCGACGTGCTGTTGACGAAGATGATCGAGCAACACTCGGCAACTGCTCACGAGCGTAAAGGAAACCCAGAT
TTTCTCGACATCGTCATGGCCAACCGAGATAACTCTGGGGGCGAGAAGCTTAGCTTGACCAACATTAAGGCTCTTCTCTT
GAATTTGTTCACTGCAGGCACCGATACTTCATCGAGCATAATCGAGTGGTCGCTGGCCGAGATGTTGAAGAACCCCAGCA
TCCTCAAGCGAGCCCACGAAGAGATGGACCGAGTCATCGGGCGGCACCGACGCCTCGAGGAATCCGACCTCCCGAAGCTT
CCGTACTTGCAGGCCATTTGCAAGGAGAGCTTCCGAAAACATCCATCGACACCGCTAAACCTACCGAGGGTCTCGACCGA
GGCCTGCCAAGTGAACGGCTACTACATCCCCAAGGACACAAGGCTGAGCGTGAACATATGGGCGATCGGGCGGGACCCAG
ACGTGTGGGAGAACCCTCTTGACTTCACCCCGGAGCGTTTCCTGAGCGGCAGGAACGCGAAAATTGACCCGCGTGGGAAT
GACTTTGAGCTGATCCCGTTCGGGGCCGGGCGGAGGATCTGCGCTGGGACGAGGATGGGGATCGTGCTGGTCGAGTACAT
ACTGGGGACGTTGGTCCACTCGTTCGACTGGAAGCTGCCGAACGGCATGGAGGAGCTGAACATGGACGAGGCGTTCGGCC
TGGCGTTGCAAAAGGCCGTACCCTTGTCGGCCGTCGTCACCCCACGGCTTTCCGTTAGCGCATATGTACCTTAAAGCTTT
CTGCGGAAAGCTTTTCCACTACGAGAATGCGACGTTATTTTAGTACTGGGAGAACTCTTAAGACTCGTGATGCGATGGGC
CGCTGGATGGGGTCTATAACGAATGTGGTGTAACTCCTTGCCCCTCTCGTGTGTACTCTTTATTATGTATTTGAAAAATT
CGGTGTTTTTATGTGGTTGTGATAATATGAACTTAAGTGTT

>*EguKAO1* transcript_HQ_hun_transcript50295/f3p0/1851

GGGGAGACTGAGGGATGTTTCTGAGTTCCACTCTCTAAAACACCTCTCTCTCTCTCTCTCTCTCTCTCTCTCTCTCACAA
CTCTGTTTCCTCATCAGCTGTTTGCGAAGAAAAGCTAATAGTTGAGGAAAAGGCTTGCGTGGCCCCATCAAAGCAGAACG
CAAGAGAGGTTCATCGCCATCCTTTGTCATGGAGCTCGCTTCCTCCTTTTCCTCTGCTTCGTTTCTGATGCTCGTGGCAT
TGATGACGCCGGCATGGATGGTTCTGGCCTGGCTTCTCAAGCATGCAAATTGGTACTTGTACGAGACCCAGCTGGACAGG
GAGCGACGACGCTCGTTACCTCCCGGTGATCTCGGATGGCCTTTCATTGGGAACATGTGGTCCTTCCTCAGGGCATTCAA
GTCTGGCGATCCTGATTCCTTCATGTCCTCCTTCGTCACCAGATTTGGTAATACCGGAATCTACAAGGCCTTCATGTTTG
GGAGCCCGAGCGTGATTGTGACCATGCCTGAGCCATGCCGAAGAGTGTTGACCGATGATGAGAAATTCCAGCCTGGTTGG
CCCATTTCAACAATGAAATTGATCGGGAGGAAATCATTTATAGGGATTTCATATGAAGAACACAAAAGGCTTAGGCGTTT
GACTGCCACTCCTGTCAATGGTCACGAATCTTTATCTCTGTATATGGGATACATTGAAGAAAATGTCATATCTGCACTTG
ATAAGTGGTCAAAGATGGGGCAAATTGAGTTCTTGACTGAACTTCGGAAGCTCACCTTCAGGATAATCATGTATATCTTT
CTTAGCTCAGAGGGTGAACTGGCGTTGGAGGCTTTAGAAAGGGAATACACCACTCTCAATTACGGTGTTAGAGCCATGGC
AATCAACCTTCCAGGATTTGCTTATCACAAAGCACTCAAGGCTCGGAAAAAATCTAGTGGCAATATTCCAGTCCATAGTGG
AGGAACGACGGAATCAGATGAAGACTAAGGTTTCTGCAAAGAAGGACATGCTGGATGCCCTACTAGAAGTGGAAGACGAA
AATGGTCGAAAGTTGGACAACGAGGAAATCATTGATGTGTTATTAATGTACTTAAATGCGGGCCACGAATCTTCCGGTCA
CATCACGATGTGGGCGACTATCTTCCTACAAGAGAATCAGGAGTTCCTCAAGAAAGCCAAGGAGGAACAAGAAGAGATCA
TGAAGAAGAGACCGCCAACACAAAAGGGTTTGACCCTGAAGGAATTTCGGCAGATGGAATACTTGTCCAAGGTCATTGAC
GAAACACTTCGCTTGATCACATTCTCACTGGTGGTTTTTCGTGAAGCAAAATCAGATGTCAGTATGAATGGTTACACTAT
TCCTCAGGGTTGGAAAGTTCTGGTGTGGTTCAGAACCGTTCATTTGGATCCCGAAATATATCCAAATCCAAATAAATTTG
ATCCTTCCAGATGGGATAATTACATACCCAGAGCAGGAAGTTTTCTTCCCTTTGGAGCAGGAAGCAGGATGTGCCCTGGA

AATGATCTCGCGAAGCTTGAAATAGCGATTTTCCTGCATCATTTCCTTCTTAACTATAGGCTCGAACGACTCAATCCCGG
ATGTGCAACAAGGTACTTGCCCCATTCGAGGCCGAAAGATAACTGCCTAGCACGAGTCATAGCACTCTCATCTCCATTCG
CATAAGAGGGAAAGACGGGCGGGAAATTTTCTCTTCGTTTCTTATTCTTCCACAGTAGTATGTTAATATCCTGTATGAGA
AACGCCGTAACAGACCTTAACGAGGCTCTCTCTGTTTTTCGGGAGAAGTATGAACTCTAAAGGAATCAAAATACAGAAGT
TGGTTCAGTGT

>*EguKAO2*   m64168e_220610_021218/111215420/ccs
GGAGACTGAGGATGTTTCTGAGTTCCACTCTCTAAAACACCTCTCTCTCTCTCTCTCTCTCTCTCCTCACAACTCTTT
TCCTCATCAGCTGTTTGCGAAGAAAAGCTAATATTGAGGAAAAGGCTTGCGTGGCCCAATCAAGCAGAACGCAAGAGAGG
TTCATGCCATCCTTTGTCATGGAGCTCGCTTCCTCCTTTTCCTCTGCTTCGTTTTGATGCTCGTGGCATTGATGACGCCG
GCATGGATGGTTCTGGCCTGGCTTCTCAAGCATGCAAATTGGTACTTGTACAGACCCAGCTGGACAGGAGCGACGACGCT
CGTTACCTCCGGTGATCTCGATGGCCTTTCATTGGAACATGTGGTCCTTCCTCAGGGCATTCAAGTCTGGCGATCCTGAT
CCTTCATGTCCTCCTTCGTCACAGATTTGGTAATACCGAATCTACAAGCCTTCATGTTTGGGAGCCCGACGGTGATTGTG
ACCATGCTGAGCCATGCCGAAGAGTGTGACGATGATGAGAAATTCCAGCCTGGTTGGCCATTTCAACAATGAAATTGATC
GGGAGGAAATCATTTATAGGATTTCATATGAAGAACACAAAGGCTTAGGCGTTGACTGCCACTCCTGCAATGGTCACGAA
TCTTTATCTCTGTATATGATACATTGAAGAAATGTCATATCTGCACTTGATAAGTGGTCAAAGATGGGAAATTGAGTTCT
TGACTGAACTTCGAAGCTCACTTCAGGATAATCATGTATATCTTTCTTAGCTCAGAGGTGAACTGGCGTTGGAGGCTTTA
GAAAGGGAATACACCACTCTCAATTACGGTGTTAGAGCCATGGCAATCAACCTTCCAGGATTTGCTTATCACAAAGCACT
CAAGGTCGAAAAATCTAGTGGCAATATTCCAGTCCATATGGAGGAACGACGGAATCAGATAAGACTAAGGTTTCTGCAAA
GAAGGACATGCTGGTGCCTACTAGAAGTGGAAGACGAAAATGGTCGAAAGTTGGACAACGAGGAAATCATTGATGTGTTA
TTAATGTACTTAAATGCGGCCACGAATCTTCGGTCACATCACGATGTGGGCGACTATCTTCCTACAAGAGAATCAGGAGT
TCCTCAAGAAAGCCAAGGAGAACAAGAAGAGATCATGAAGAAGAGACCGCAACACAAAAGGTTTGACCTGAAGGAATTTC
GGCAGATGGAATACTTGCCAAGGTCATTGACGAAACACTTCGCTTGATCACATTCTCACTGGTGGTTTTTCGTGAAGCAA
AATCAGATGTCAGTATGAATGGTTACACTATTCTCTGGGTTGGAAAGTTTGGTGTGTTCAGAACGTTCATTTGGATCCCG
AAATATATCCAAATCCAAATAAATTTGATCCTTCCAGATGGATAATTACATACCAGAGCGGAAGTTTTCTTCCTTTGGAG
CAGGAAGCAGGATGTGCCCTGGAAATGATCTCGCGAAGCTTGAAATAGCGATTTTCTGCATCATTTCTTCTTAACTATAG
CTCGAACGACTCAATCCGGATGTGCAACAAGTACTTGCCCCTTCGAGCCGAAAGATAACTGCTAGCACGAGTCATAGACT
CTCATCTCATTCGCATAAGAGGGAAAGAGGGCGGGAAATTTTTCTTCGTTTCTTATTCTTCCACAGTAGTATGTTAATAC
TCTGTATGAGAAACGCCGTAACAGACCTTAACGAGGCTCTCTCTGTTTTTCGGGAGAAGTATGAACTCTAAAGGAATCAA
AATACAGAAGTTGGTTCAGTGTACTTGGAATGTTTCATGGGTTCAGCGCCATTCAGCTGTAAAGACAGCAAAATCGCAGG
ACGGTGGAATCGATCTCTTTTCATGC

>*EguKAO3*   m64168e_220610_021218/25034999/ccs
GGGGAGACTGAGGGATGTTTCTGAGTTCCACTCTCTAAAACACCTCTCTCTCTCTCCTCTCTCTCTCTCTCTCACA
ACTCTGTTTCCTCATCAGCTGTTTGCGAAGAAAAGCTAATAGTTGAGGAAAAGGCTTGCGTGGCCCCATCAAAGCAGAAC
GCAAGAGAGGTTCATCGCCATCCTTTGTCATGGAGCTCGCTTCCTCCTTTTCCTCTGCTTCGTTTCTGATGCTCGTGGCA
TTGATGACGCCGGCATGGATGGTTCTGGCCTGGCTTCTCAAGCATGCAAATTGGTACTTGTACGAGACCCAGCTGGACAG
GGAGCGACGAACGCCGTGACCGTGACCTCCCGGTGATCTCGGATGGCCTTTCATTGGGAACATGTGGTCCTTCCTCAGGGC
ATTCAGTCTGGCGATCCTGATCCTTCATGTCCTCCTTCGTCACCAGATTTGGTAATACCGGAATCTACAAGGCCTTCATG
TTTGGAGCCCGAGCGTGGTTGATTGTGACCATGCCTGAGCCATGCCGAAGAGTGTTGACCGATGATGAGAAATTCCAGCC
TGGTTGGCCCATTTCAACAATGAAATTGATCGGGAGGAAATCATTTATAGGGATTTCATATGAAGAACACAAAAGGCTTA
GGCGTTTGACTGCCACTCCTGTCAATGGTCACGAATCTTTATCTCTGTATATGGGATACATTGAAGAAAATGTCATATCT
GCACTTGATAAGTGGTCAAAGATGGGGCAAATTGAGTTCTTGACTGAACTTCGGAAGCTCACCTTCAGGATAATCATGTA
TATCTTTCTTAGCTCAGAGGGTGAACTGGCGTTGGAGGCTTTAGAAAGGGAATACACCACTCTCAATTACGGTGTTAGAG
CCATGGCAATCAACCTTCCAGGATTTGCTTATCACAAAGCACTCAAGGCTCGGAAAAATCTAGTGGCAATATTCCAGTCC
ATAGTGGAGGAACGACGGAATCAGATGAAGACTAAGGTTTCTGCAAAGAAGGACATGCTGGATGCCCTACTAGAAGTGGA

AGACGAAAATGGTCGAAAGTTGGACAACGAGGAAATCATTGATGTGTTATTAATGTACTTAAATGCGGGCCACGAATCTT
CCGGTCACATCACGATGTGGGCGACTATCTTCCTACAAGAGAATCAGGAGTTCCTCAAGAAAGCCAAGGAGGAACAAGAA
GAGATCATGAAGAAGAGACCGCCAACACAAAAGGGTTTGACCCTGAAGGAATTTCGGCAGATGGAATACTTGTCCAAGGT
CATTGACGAAACACTTCGCTTGATCACATTCTCACTGGTGGTTTTTCGTGAAGCAAAATCAGATGTTACACTATTCCTCA
GGGTTGGAAAGTTCTGGTGTGGTTCAGAACCGTTCATTTGGATCCCGAAATATATCCAAATCCAAATAAATTTGATCCTT
CCAGATGGGATAATTACATACCAGAGCAGGAAGTTTTCTTCCCTTTGGAGCAGGAAGCAGGATGTGCCCTGGAAATGATC
TCGCGAAGCTTGAAATAGCGATTTTCCTGCATCATTTCCTTCTTAACTATAGCTCGAACGACTCAATCCCGGATGTGCAA
CAAAGGTACTTGCCCCATTCGAGGCCGAAAGATAACTGCCTAGCACGAGTCATAGCACTCTCATCTCCATTCGCATAAGA
GGGAAAGACGGGCGGGAAATTTTCTCTTCGTTTCTTATTCTTCCACAGTAGTATGTTAATATCCTGTATGAGAAACGCCG
TAACAGACCTTAACGAGGCTCTCTCTGTTTTTCGGGAGAAGTATGAACTCTAAAGGAATCAAAATACAGAAGTTGGTTCA
GTGG

>*EguGA20ox1*   m64168e_220610_021218/134220437/ccs

GGGGTATCGACCCCTGCAACTGCAATGCGCAGATTCTGAATTACAGACCTCAGAACAAATCTCTGTCCGGGTCCTCTTGT
CCTAAAAGATTGTCCCTCCTTACATGAACGAATCACCATCATCTCTCTCTCTCTCTCTGTGGCTCATTTAAGGTCCTGTA
ATTTTGCCTCAAGAGCTTGTAGCTTCCTCCAAAACATTCCCCTTGTCTCTTTTCCCAAGGGCAGAAGTTCCCTGACTCCC
AATCAAAATTTGGTTTTTCAATTCCCCTCAGGTAAAAGGGAAAGAAGATCCTCAACAAAAGTTCCTTCTTTCCCCAAATC
ACCATCAGTTCAGTCCCTCCCGCAAGAACTCACCGTGCTCTTTACCACTACAACAAAAGCTTCGGTTTTTATTTCTTAGT
GTGAAAAGTAGATGCAAAATATTCCCTGCCCATTATTTCGTCCCCATTTTTTTCAGCCACTTTGCTCTGTTGTGAGCCAA
ACTTGGAATGGCGTAGCTGACTCTAAATGATTTTGATTGGGCAGATACAGAAGACAGGGTGACATTAGCACTCCTTCTCT
TTCCTCCAAAGAAACGCCAAAAACGCATTGGGAAAAGCTCTTTTGGCAACTACAGAAAGGACAACACTCCTAGAATCAAA
TTGGGGCAATATAATAGTGTGAAAATTACTAAAATCTTTTTTGTCAAATTGACAAGCACCAACCGATGCAAATTCACTCT
CTTTGTGGTCCTCGTTTGCAAACAAACAGCTTACACAGCTCCAGATAGAGAGCTATAAAGATTCCTACATTTTGTGCACT
CATCTCCCCCCATAAACGGCCTTGGCTTTTCTTTTATCCCAACTCTCAAATTTATTCCCCGCCCACTCCTCCCTCATTTC
CCCTGCACAGGAAAAAAGTCGGCTCACATATATAGCTTCCTGAATGCAATGGCAGTTGATTGCCTCACAAGTAAAACCTC
ACCAGCCATGCCTCCGCAGCACAAAGATGAAGCCAGAGAGGATAAAAAACATCTAGTTTTGACGCCTCAGTGATCCGGCA
CCAACCGACATCCCGAAACAGTTCATTTGGCCCGACGAGGAAAAGCCGTGTGCGAACGCCCCGGATCTCGCCGTCCCGCT
CATCGACTTGGACGGGTTCCTCTCCAAAGACCTGAGTGCCTCCGAGGAGGCATCGAGGCTCGTGGGGGATGCGTGCCAGA
AGCACGGCTTCTTCCTTGTCGTCAATCACGGCGTGGATGCTGGCCTCATATCGGACGCTCACAAGTACATGGACAAATTC
TTCGGGTTACCGCTCAGCGAGAAGCAGAGGGCTCAGAGGAAGCTCGGTGAGCATTGTGGATATGCCAGCAGTTTCACTGG
CAGGTTCTCTTCCAAGCTCCCATGGAAAGAAACGCTTTCCTTCGGCTACTCCGCCGAGAAAAGCTCGGCCAATGTCGTGG
AAGACTACTTCAAGAACACCATGGGCGAAGAGTTTGAGCAATCCGGGAGGTGTACCAGGACTATTGTGAGGCCATGAGCA
GACTGTCTCTAGGAATAATGGAGCTGCTAGAATGAGCCTAGGAATCGGCAGAGACCATTTCAGGGAGTTCTTCGAAAGCA
ACGATTCGATCATGAGGCTCAACTACTACCTCCGTGCCAGAAGCCGGACCTCACCCTAGGAACCGGTCCCCACTGCGACC
CGACATCCTTAACCATCCTACACCAGGACCAAGTTGGCGGGCTCCAAGTGTTCGTCGACAACGAGTGGCGTTCCATCAGC
CCGAACTTCAACGCGTTCGTCGTCAACATCGGCGACACTTTCATGGCTCTATCAAACGGGCTATACAAGAGCTGTTTGCA
CAGAGCAGTGGTGAACAGCCGAACTCCGAGGAAGTCCTCGCCTTCTTCTTGTGCCGAGGAGCGACAAAGTGGTGAGACCA
CCGAGTGAGCTAGTCGCAATGTCCTGTCCGAGAGCGTACCCGGACTTCACATGGCCGGTGCTCCTCGAGTTCACTCAGAA
GCATTACAGGGCCGACATGAACACGCTCCGAGCATTCACCAACTGGCTTCAACAGAGAACATCTGAACCAGTTCGTTGAT
GAAGATTTGTCACAAGTAGAGAGATCTATTTGGAGGTCCGAAAAGTTGCGGCTAACAAAGGGGTGAAAGAGCCTCTCTGC
CAAAGGAAAGAAGATGACATTGACGACGACAAAGAACAGAGATCAAAAGGAAGTGGTGGGTTTTTTTGAAGGACGTTGGA
GAGGGACAAACAGAGAGTTAGAGGAAAAGCCAAATATCTTTTACGTTCAAGGGTACGTCTTCTGTAGCCAGATAGTACTG
GCACCATGATGGTCACGATGATCAAAGGCAAGAGATCAAGAAACATGAGAACCAATAAAGAGCTGTAATACATCAGCTAA
TTTTTGTTTTGTTTTTTTTCCTTCTCCTTTTGTCCTTGGTTAATGTAGAAAAGTTTACCCAACAGCAACCCTGCCTTGAT
GTAAATTTGCATTTTGGAGAATAGATGCTTCATTAAATATTCCTAGTTTTTTC

＞*EguGA20ox2* **transcript_HQ_hun_transcript21786/f2p0/2931**

GGAATAAAAAAACATGTCCTTCTTATTGGACACAAGAACTTCCCCATCAACCCATGTTTATGTCCTGCAATGGAGCTAA
AGGATGGGAAGGAGACCCTCGTATTCGATTCCTCCTTGCTTCAAAAGCAACCAAACTTGCCAAAAGAGTTCATTTGGCCA
CACAAAGACCTTGTTCGCACCCAAGAAGACCTCAAAGAGCCACTCATAGACTTGTCAGGCTTCCTAAAAGGCAATGAAGA
AGCAACTTCCCGAGCAGCTGAGCTCATCAGGACTGCTTGCACCAACCATGGCTTTTTCCAAGTCATCAACCATGGAGTCG
ACCCAGGCCTCATAAGCGCCGCCCACAATGAGATTGACTCGATTTTCAAGCTCCCCATAAGCAAGAAGCTCGGCATGAAG
AGGAGGCCCGGTGGAGTGTTTGGTTACGCCGGTGCTCATGCGGATCGGTACTCATCCAAGTTGCCATGGAAGGAGACTTT
CTCTTTTGAGTTTCGCAAGAACGAGAGCTCTGAGCTTTTGGTGGTTGATTACTTCAAATCTGTCTTGGGCGATGATTTTG
AACATACAGGATGGGTGTACCAGAAGTATTGTGAAGCGATGAAGGAGCTAGCACTGGTGATCATGGAGTTACTGGCGATA
AGCTTGGGGATCGACCGCATGCACTACAGGGACTTCTTCGAGGACGGGAGCTCAATAATGAGGTGCAATTCGTACCCGCC
GTGCCTGAACCACGGGCTCGCGATGGGCACGGGTCCTCACACCGACCCGACATCCTTGACCATCCTCCACCAGGACCAAG
TCGGTGGCCTCCAAGTCTTTGCCAGCAACAAGTGGCAAGCCATCACGCCTCGTCAAGACGCACTCGTCATCAACATTGGA
GACACTTTCATGGTACTTTCCCACAACTTTTTCCCAACTCCATTTGAAAGTTCTAAACTTTGATAAATCTAGTAATTACT
GTTATTTTTGCAATGAGTCGGAGAATTTTTCAAAATACACTCATCGATATCATCATCATCCTGACTCTTTTCCAATATAT
AATGATCATGCAACATAATTCTGTTGTTTTTCGAATAGTAAGAATGAGCAAAGTAATGACCGACATAAAATATCACTTAT
TACTTAAATACAGGATTGTAATAAGTCTCATACTCACATGAAAGAATCCAACTTGAAAAGTTCTTGAATTGGAATTAGGT
GAGCCTGTTTCCACATCCATCCTGGCCCAATTGTATATCAACGTATTTTGGGTGTGGCTCTTGTTCATCCTATTCTCATT
TCCGCAATTTCATTCCTGTTGTTTATAGGGATTTATTGTGATATACATGTGAGAGCGGGTGTTATAAAATACCAAACATG
CTTAGGTACGGGATTATAATAGTATTTGTATCAATGTAACTTACTTTCATTTAAAAATTTAACTTTGGGGTTAACTTTCT
AACAACCTCAAAGGTCACATGTCTAAAGTTTGGAGATGACGAATTTTAGGACTTGCAATATAGATGATTTACGTAAGAGT
CCAAAATAGAACGATGAAAAAGTTGCTCATCAATTAGCACTTTATAATATGTTAAAGTGAACCTATCATAAAAAGAGAAG
TTGAAAAAAGATACACGGCAAGCCAGGAAAAAAGGCTTGCACAATCCGAGTAATTCTAACTAAAAGTAGTCCATACTGTC
CAGTCAAATAAATCATCCCCGGTCGGTTCATATATAGTCGACTTGTGGCGCTATTCTTGTCCGTTCTTGATACTAAAAAT
TGTACTTTTATTGGTTCTCGTCCTAGTAATGCAAAATCTATCATTCTTGACAATGATTTCACGCGCCACAACTAAAACAG
GCAGGATGAAATTGGCCGATGAAGTGGTTATACATCGTTGGGTCAATAACAACGGGAGTAGCTAGAAATAATAATACTAA
CTTGTCAACGTTGGAACAGTAGTTACCTCGACCTCTATTTTTCGTCTTGTGTTTTAGCTGTTCCGCTGAATCATCAGCCA
ACTTGTCCTTCGACCGTATGTGCTTGCCAATGAACATGCAAGGCGACGTGGGCACTGGGGGGACCACTAGTTCTGTTGCC
TCCATCTCCATAATAATTTAGGGTTTGTTCAAGATGAGAGTGTGATGCGTAAGGAGCTTTAAGAGTGAAACTAAAGCATT
ACTGAGACAAGAGGAGGGCGAATTGTTTTTTTTTTTTTTTGGTAAAAGGTAAGAAATATATTAAGGAATGGCCAAATGTA
CAAAAGATCTCGACACCAGGAACTTGCACTCCAAATGTAGTAACAAAAGAAGTGGAAGGGTGACGAGGGTGAATTGTTTT
CTTCTGAGATTTCACCCAAAACCCTAGGACACCATCCATGGTTTCAATATTTGTCGAGTTAGAGTTAGAAAGCCATCTCA
ACAGTTGTTTTAATGGCAATTGATCGAGAAAGTATTGAGATGCAGGCATTAACGAATGGGATGTACAAGAGCTGCTTGCA
CAGGGCGGTGGTGAACAGGGAGAGGGAGAGGAAGTCACTGGTGTTCTTCGTGTGCCCGAGAGAGGACCGGATTGTGAGAC
CCCCACCAGATCTTCTTGGGATCGACATGGAAGTGCCAAGGAAGTACCCGGACTTCACATGGTCCGACCTCTTTGAGTTC
ACGCAGACCCACTATCGCAGTGACGTCGCTACCTTCCAAAGCTTCATCCACTGGTTCCTATCTCCCAAAGCCTAAATCTC
TCTCTAGTTTTGGGCATGCATGGACTTTGGATGCTGTGGATCATCATATGGTTTGTCTTATATGTGTCATCACTTTGTCC
CATCTCTCGTCTGTATAGATCCCCCCCAATCCCCCATATTTTGGATCATTTGGGCGGTTATATCATTGTGTGTACGTGTC
GGACTCTCTCAGCTTTCTTGCCACATTATTATTTAATGGTATTTTTTTTCT

＞*EguGA20ox3* **m64168e_220610_021218/78577894/ccs**

GGGATCCAACTCTCAAATTTATTCCCCGCCCACTCCTCCCTCATTTCCCCTGCACAGGAAAAAAGTCGGCTCACATATAT
AGCTTCCTGAATGCAATGGCAGTTGATTGCCTCACAAGTAAAACCTCACCAGCCATGCCTCCGCAGCACAAAAGATGAAG
CCAGAGAGGATAAAAAACATCTAGTTTTTGACGCCTCGGTGATCCGCGGCACCAACCCGACATCCCGAAACAGTTCATTT
GGCCCGACGAGGAAAAGCCGTGTGCGAACGCCCGGATCTCGCCGTCCCGCTCATCGACTTGGACGGGTTCCTCTCCAAAG
ACCCGAGTGCCTCCGAGGAGGCATCGAGGCTCGTGGGGGATGCGTGCCAGAAGCACGGCTTCTTCCTTGTCGTCAATCAC

GGCGTGGATGCTGGCCTCATATCGGACGCTCACAAGTACATGGACAAATTCTTTCGGGTTACCGCTCAGCGAGAAGCAGA
GGGCTCAGAGGAAGCTCGGTGAGCATTGTGGATATGCCAGCAGTTTCACTGGCAGGTTCTCTTCCAGCTCCCATGGAAAG
AACGCTTTCCTTCGGCTACTCCGCCGAGAAAAGCTCGGCCAATGTCGTGGAAGACTACTTCAAGAACACCATGGGCGAAG
AGTTTGAGCAATCCGGGTACGAAACCTATCGCCCATTTCTTTCGTGACAATCGAGCATGTTCCTCGGTCGTCGAAAGAAA
ACATAATTTCCCCCACACTTTATTAGCATCAATCTATGAAGAGATTAGTCAATAACTCTATAGTTTTATTGTGTAGTGTG
TACCAAGACAGATGATGATTTTGGTTTTCTTGTGTTTCAGGAGGGTGTACCAGGACTATTGTGAGCCATGAGCAGACTGT
CTCTAGGAATAATGGAGCTGCTAGGAATGAGCCTAGGAATCGGCAGAGACCATTTCAGGGAGTTCTTCGAAAGCAACGAT
TCGATCATGAGGCTCAACTACTACCCTCCGTGCCAGAAGCCGGACCTCACCCTAGGAACCGGTCCCCACTGCGACCCGAC
ATCCTTAACCATCCTACACCAGGACCAAGTTGGCGGGCTCCAAGTGTTCGTCGACAACGAGTGGCGTTCCATCAGCCCGA
ACTTCAACGCGTTCGTCGTCAACATCGGCGACACTTTCATGGCTCTATCAAACGGGCTATACAAGAGCTGTTTGCACAGA
GCAGTGGTGAACAGCCGAACTCCGAGGAAGTCCCTCGCTTCTTCTTGTGCCCGAGAGCGACAAAGTGGAGACCACCGAGT
GAGCTAGTCGCAATGTCCTGTCCGAGAGCGTACCCGGACTTCACATGGCCGGTGCTCCTCGAGTTCACTCAGAAGCATTA
CAGGGCCGACATGAACACGCTCCGAGCATTCACCAACTGGCTTCAACAGAGAACATCTGAACCAGTTCGGTGATGAAGAT
TTGTCACAAGTAGAGAGATCTATTTGGAGGTCCGAAAAGTTGCGGCTAACAAAGGGGTGAAAGAGCCTCTCTGCCAAAGC
AAAGAAGATGACATTGACGACGACAAAGAAGAGAGATCAAAAGGAAGTGGTGGGTTTTTTTAAAGGACGTTGGAGAGGG
ACAAACAGAGAGTTAGAGGAAAAGCCAAAATATCTTTTTACTTCAAGGGTACGTCTTCTGTAGCCAGATAGTACTGGCAC
CCATGATGGTCACGATGATCAAAGGGCAAGAGATCAAGAAACAGGAGAACCAATAAAGAGCTGTAATACATCAGCTAATT
TTTGTTTTGTTTTTTTCCTTCTCCTTTTGTCCTTGGTTAATGTAGAAAAGTTTACCCAACAGTAACCCCTGCCTTGATGT
AAATTTTGCATTTTGGAGAATAGATGCTTCATT

＞*EguGA20ox4*　m64168e_220610_021218/180553832/ccs

GGGATAAACGGCCTTGGCTTTTTTTATCCCAACTCTCAAATTTATTCCCCGCCCACTCCTCCCTCATTTCCCTGCACAG
GAAAAAAGTCGGCTCACATTATAGCTTCCTGAATGCAATGGCAGTTGATTGCCTCACAAGTAAAACCTCACCACCATGCC
TCCGCAGCACAAAGATGAAGCCAGAGAGGATAAAAAACATCTAGTTTTTGACGCCCGGTGATCCGGCACCAACCCGACAT
CCCGAAACAGTTCATTTGGCCCGACGAGAAAAGCCGTGTGCGAACGCCCCGGATCTCGCGTCCCGCTCATCGACTTGGAC
GGGTTCCTCTCCAAAGACCCGTGCCTCCGAGGAGGCATCGAGGCTCGTGGGGGATGCGTGCCAGAAGCACGGCTTCTTCC
TTGTCGTCAATCACGGCGTGGATGCTGGCCTCATATCGGACGCTCACAAGTACATGGACAAATTCTTCGGGTTACCGCTC
AGCGAGAAGCAGAGGGCTCAGAGGAAGCTCGTGAGCATTGTGGATATGCCAGCAGTTTCACTGGCAGGTTCTCTTCCAAG
CTCCCATGGAAAGAAACGCTTTCCTTCGGCTACTCCGCGAGAAAAGCTCGGCCAATGTCGTGGAGACTACTTCAAGAACA
CCATGGCGAAGAGTTTGAGCAATCCGGGAGGGTGTACCAGGACTATTGTGAGGCCATGAGCAGACTGTCTCTAGGAATAA
TGGAGCTGCTAGGAATGAGCTAGGAATCGGCAGAGACCATTTCAGGGAGTTCTTCGAAAGCAACGATTCGATCATGAGGC
TCAACTACTACCCTCCGTGCCAGAAGCCGGACCTCACCCTAGGAACCGGTCCCCACTGCGACCCGACATCCTTAACCATC
CTACACCAGGACCAAGTTGGCGGGCTCCAAGTGTTCGTCGACAACGAGTGGGTTCCATCAGCCCGAACTTCAACGCGTTC
GTCGTCAACATCGCGACACTTTCATGGCTCTATCAAACGGCTATACAAGAGCTGTTTGCACAGAGCAGTGGTGAACAGCG
AACTCCGAGGAAGTCCCTCGCCTTCTTCTTGTGCCCGAGGAGCGACAAAGTGGTGAGACCACCGAGTGAGCTAGTCGCAA
TGTCCTGTCCGAGAGCGTACCCGGACTTCACATGGCCGGTGC**TCTC**GAGTTCACTCAGAAGCATTACAGGGCCGACATGA
ACACGCTCCGAGCATTCACCAACTGGCTTCAACAGAGAACATCTGAACCAGTTCGGTGATGAAGATTTGTCACAAGTAGA
GAGATCTATTTGGAGGTCCGAAAAGTTGCGGCTAACAAAGGGTGAAAGAGCCTCTCTGCCAAAGCAAAGAAGATGACATT
GACGACGACAAAGAAGAGAGATCAAAAGGAAGTGGTGGGTTTTTTTAA<u>AGG**CT**</u>TGGAGAGGGACAAACAGAGAGTTAGAG
GAAAAGCCAAAATATCTTTTACCTTCAAGGGTACGTCTTCTGTAGCCAGATAGTACTGGCACCCATGATGGTCACGATGA
TCAAAGGGCAAGAGATCAAGAAACATGAGAACCAATAAAGAGCTGTAATACATCAGCTAATTTTTGTTTTGTTTTTTTCC
TTCTCCTTTTGTCCTTGGTTAATGTAGAAAAGTTTACCCAACAG

＞*EguGA2ox1*　LOC104450041

CCATCATCTTCAAAACAACTGTCTCTGTCTCTCTCTCTGTCTGTCTGTCTCTCTCTCTCTCTCTCGATTCTCTCCCTAGT
CCATTAGTTCTCGTTGCCGCTTCGTAAACAAGGAAGCACGGCGCACGGCCGTCCGATGGTTGTCCCGTCCAAACTAGCAA
TTGAACAGTTCTCCTACGTTATGAACAGCAACGCATTATCATCCCACCAAATCCCTGTCGTGGACCTCTCGAAGCCCGAC
TCCAAGAGCCTCATCATCAAGGCCTGCGAAGAGTGCGGCTTCTTTAAGGTCGTGAACCACGGCGTCCCGTTGGATTTTAT
CTCCAGGCTGGAGGAGGAAGCCGTCAAGTTCTTCTCTCTGCCTCTCCCCGAGAAGGAGAGGGCAGGCCCTCCTGACCCGT
TCGGCTATGGCAACAAGATGATTGGCCGGAACGGAGATGTGGGTTGGATCGAATACCTCCTCCTGACGACCGATCCCAAC
TTCAACTACCGCAAGCTCCCATCGGCTTTTAACGAAAACCCAGAAAGATTTCGCTCTGCTTTGAGTGATTACACATCGGC
GGTGAGGTACATGGCGTGTGAGATTCTCGAGTTGATGGCCGACGGATTAAGGATTCAACAGAGGAATATATTTAGCAAAC
TTTTGATGGATGAACAGAGCGACTCTGTTTTCAGGCTCAACCACTACCCTCCATGCCCGGAGCTTCAATCCTATGTCGAT
AGGAACATGATTGGATTCGGTGAACACACTGACCCACAAATCATATCTGTTCTCAGATCGAACAACACGTCTGGGCTCCA
AATATCCATGAAAGATGGGACTTGGGTTTCTGTTCCACCGGACCAGAACTCATTCTTCATCAATGTTGGTGACTCCTTAG
AGGTGATGACTAACGGGCGATTCAGAAGCGTGAGGCACAGAGTCCTGGCGAACACCTCGAAGTCCAGGGTCTCGATGATA
TACTTCGGAGGACCACCTTTGAGTGAGAAGATAGCGCCATTGCCGTGCCTCATGAAGGGCAAAGAGAGCCTGTACAAGGA
GTTTACATGGTTCGAATACAAGAAGTCCGCCTACAACACGAGGTTGGCTGATAACAGGCTAGAGCATTTTCAGAGAGTAG
CCGCTTCTTGATGTCGCTCAGAGCGCCAGATGTCAGCAGCAAGAATGGGTTCTTAGGACAGCAACTTTCATCTTCATTTT
GTTCCTTCCTTGTCTCTCTGTATTTTCCATCGGTACTTTCTTGTTCAAACGATGTAAATTACTCTCTTCTTGTCAAATAT
CACAGAGCGTCCATGGTCTGCCACTATCTCTATTTGACAATTTGTAATATGTAATTTTCAATGAAGTCACAGTCACAAGT
CACCTTTCAGACAGACGAGGAAATTGAAAAGGTTCATTTTC

＞*EguGA2ox2*　LOC104440788
TCTCCTGACCCGTTTATAAATACCAACTCATCGTCGTCTCAGCTCCGCATCATACCATCATCATTGTCTTTCCTTCTTTC
TTCTTCTTCTTCAGTCATGGTGGTTCTTTCTCCAGGAGCGATCGACCAATTTGCCCTCATCAAGACGTGCAGGCCGAGCG
GTGCCGTCTTCGATGGGGTCCCCGTCGTCGACCTGTTGAGGCCCGACGCCGCCACGGTCGTGGTCCGGGCGTGCGAGGAG
TTCGGGTTCTTCAAGGTGGTCAACCATGGGGTCTCGCTGGAGGTCATAAATAGGTTGGAGGACGAGGCGGTGAAATTCTT
CGGCTTGCCGCAGTCCGAGAAGGACAAGGCCGGTCCTCCCGACCCTTTTGGCTACGGGAACAAGCGGATTGGCTGGAACG
GCGACGTCGGCTGGATCGAATACCTCCTCCTACACAGCAACTCTAGCGAGGATGACCAGAAACTGTCTCCCTCCTTTTCT
CAGGGAAACCAGGAATTTTTCCGATGTGCAGTGGAGGAATACGTGAGAGCAGTGAAGGAGGTGGCGTTCCGAGTGGCGGA
ATTGATCGCCGACGGGCTCGAGATCGAGCCGAGGGACGTGTTGAGCAGCATGTTGAGGAGCGAGGCGAGCGACTCGTGCT
TCAGGCTGAACCATTACCCGCTGTGCCCGGAGCTGCAGGCGCTGACCCCGAGCCCGAGTAGTCGCAACCTGATCGGGTTC
GGGGAGCACACGGACCCGCAGATAATTTCTATCCTGAGATCCAACAACACGTCGGGCTTCCAGATATGCCTCCGGGACGG
GGCTTGGGTCTCTGTCCCACCCGATCACTCCTCCTTTTTCATCAATGTTGGTGATTCTTTGCAGGCATTGACCAATGGAA
GGTTCTGGAGTGTGAAGCACAGGGTGCTGGCTGGCCATGGCAAATCAAGGATTTCAATGATATACTTTGGGGGACCAGCT
TTAGATGAAAAGATAGCACCTTTGCCCTCCCTGGTGGCTGAGGGGGAGAGCAGCTTGTACAAGGAGTTCACATGGTCTGA
ATACAAGAGATCTGCTTACAAAACTAGGCTGGCTGATTACAGGCTTGGACTCTTTGAAAAACCAAGGGTCCAGTGATTAC
TTTTCTTCTTGTTCAAGATTCAAGAAAACAAAACAAAAGATTAACCCCATTGGAGCAAAATCATATACTAGGGTTTTTTC
TTCTTCTTGAAGCTTTTCAACTAGCCACAGATTGGAAGCCAAACAGGGGGTTTGACTGGTCCTTTCTTGATTAGATTCTT
TTTTTGCCAACCTTTTTCCTCATTAACTAGTGTTTTGCTCATTTCCCCTTTGCCCAAATAATGATATGAGGACATGATGT
AATTTCATCACCTGAACCGAATTGTTCCTTTTACACCCTCTCACCGTGGGCCTAGCATATGCAGGAACTCAGATTCAGTA
AATGATGGAGTACATGGCAAAGGAAGAGGTGCACTCTAGTATCCTCCTTTATATGGAAGTCCTTAACTCAAGAATGTAAG
CTGTTGACTGGTGGAGGGCACATGTGTCTGATTGTATATTGGTCGAGGGGGCAGTGGTATCTGACCCGAATTGATAGGAA
ATAG

＞*EguGA2ox3*　LOC104434098
CTCTCGTGTCCAAGTAAAAGGAAAACGAAAAGAGACAGAAAACAGAAAAGCGGCGGCGATGGTGGTGGCGAGTCCGAACC

CACGACGGGCCGAGAAGATCCAGGCCCTCGAGCTCCCGGCGATCGACCTCTCGCCGTCGGGGAGATCCGCGGCGCCGCGG
CTCATCGTGGAGGCCTGCGAGAGGTACGGCTTCTTCAAGGCGGTCAACCACGGCGTCCCCGCCGAGATCGTGTCGAGAAT
GGACGAGGCGAGCGCCGGCTTCTTCGCGCGGCCCGCCTCCGAGAAGCGGCTCGCCGGGCCCGCCGACCCGTTCGGGTACG
GGAGCAAGAGCATCGGGTTCAACGGCGACGTCGGCGAGGTCGAGTACCTCCTCCTCGAGTCCGACCCCGCCTTCGTCTCC
CGCAGGTCCGCCAGCATCTCCGACGACCCCACCCGGTTCAGCGCTGCTGTGAATGTTTACATAGAAGCAGTCAAGGACCT
GGCCTGTGACATATTGGATCTTATGGCCGAGGGCCTGGGGGTCCGGGACACGTCGGTTTTCAGCAGGCTCATCAGGGCCG
TCGACGGCGACTCGGTCTTCCGGATCAACCACTACCCCCAGTGCGTGGTCCTTCACGGCGAGGTCGGGTTCGGGGAGCAC
TCCGACCCTCAAATCCTGACCGTCCTCCGATCCAACAATGTGGGCGGCCTCCAGATCTCACTCGAAGACGGGGTGTGGAC
CCCGGTGCCCCCAGACCCCGCAGCTTTCTGGATCAATGTGGGCGATCTTTTGCAGGCCATGACGAACGGGAGGTTCTCGA
GCGTGCGGCATAGGGCGGTAACCAACCCCTTCAGGTCCAGAACGTCGATAGCGTTCTTCGGGGCGCCTCCGCTGGACGCG
CGGATCGCTCCCCAGCGGGAGCTCGTCACTCCTCGAAGGCCCCGTCTCTACAACCCCTTCACCTGGGCCGAGTACAAGAA
AGCCGCCTACTCCCTCAGGCTCGGGGACAAGCGTCTCGACCTCTTCAAGGCCTGCAGAGAAGACGGCGGCATCGATCTGT
GAGCAGATGGAGGAGATGGGTCGTCTCTTTTCTGCCCTTTTCTCTCTCTCTTGTTGCTGGGCCTGTCGTGAAGGGAATTT
TGGGTTTGCGTGTTCTGCTCCCCTTCCTCTGTTTTTAGCAGCAAAGAGAAGCTCTCCTAGTGTTGGTGTACTGTTGTAAT
CAATGGAAAGGTATGTTAGGCGACGATATTATGTTTTGGCTTTTATCTATCAATCGACCCATCGGTTGATTTTATCTATC
CTCGTATTTGTATCTATTGCACTAGACATATTGAGAAAGCTTACGTACACTCGA

>*EguGA2ox4*　LOC104426836

CATTATAAATATATATATGGAGTGTTATCAACTTACCTATGATCATCATACCCTCCAAAGTTCAAGGTTCAAATTCTCCC
TGCTTCTTCCATTTCAACTTCCTGGCTCTCCACCATACCTCCCCTAGATTTCTCCCCTACAGTTCAGCTCATCACTTTTG
AACTAGAGAGAGAGAGAGAGAGAGAGAGAGAGAGAGAGAGAGAGAGAGAGAGAGAGAGAGAGACAGAAATGGTGATGCCGTCTCCT
AGCCCGATTCGGACCAAGAAAACCTGGGCGGTCGGGATCCCGACCGTCGACCTCTCCCTCGGTCGGCCTGAGCTGTCCGA
GCTGATCCTGAGGGCCAGCGAGGAATATGGCTTCTTCAAGGTCACGAACCACGGCGTGTCGCAGAGGGCGGTCTCGAGGC
TGGAAGAGGAAGTGGTCGAGTTCTTCGAGAAGCCCGCGCCGGAGAAGCAGCGGGCGGGGCCGGCCAGCCCGTTCGGATAC
GGATGCAAGAACATTGGGCCCAATGGCGACATGGGCGAGCTCGAGTATCTCCTCCTCCATTCGGATCCCATCTCCATTGC
CGAGAGATCCAAAACCATCTGCGGCGACCCGGGCAATTTCAGTTCTGCGGTGAATAGCTACATAGCTACGGCTCGCGAGC
TGACGTGCGAGATACTGGACCTGCTGGCGGAAGGACTGTGGCTCTCGGATAAGTACGCCTTGAGCGGCCTCATCAGAGAC
CGGCAGAGCGACTCCCTCCTCCGGATCAACCACTATCCTCCGGCCAAGGACGTCAAGAACATCGGCTGGGACCTCTCGAC
GAGGCTCGACCAGCACTCACGCGGCGGCAACGGGCGGGTTGGGTTCGGGGAGCACTCCGACCCCCAGATCCTGACCCTCC
TGTGGTCCAACGACGTGGAAGGCCTCCAGATATGCCTGCCCGACGGCCTGTGGATCCCCGTCCCTCCCGACCCCAACGCG
TTCTACGTCATGGTCGGCGACATGCTTCAAGCGCTGACAAATGGGAGGTTGAAGAGCGTGAGGCATCGAGCGATGGCAAA
CTCGCCAAAGCCAAGGTTGTCGCTGGTGTATTTCGGAGCCCCACCAGTGAATGCTTGGATCTCTCCTTTGCCCGAGCTGG
TCTCCCCACGAGCCCCCACTCCCACTCTCTACAGGCCATTCACTTGGGGCGAGTACAAGAAGGCCATGTACTCGCTCCGG
CTCAGTGACTCTCGCCTCGACCTCTTCAAGATCCAAAGCAGCAGCAACAAGATCGCTTCATGATCACAGCATTAGTATCA
TATAAATTAAAAACTCGGTCAACTGCTAGAGGAGCCCACAGCTACAGATCATTAAAGAAGGGCAAAAGAAAGGAGAGATA
GGTGGAGAGATGTACATCAGTTAGAGATTTAGTTCAAGGGTGTTTCCTTTTCTTTTTGTTTTTTTTCCTTGGGTAGGATC
GATAGGTTTTTACCTTTTCTTCTTTTTATTTTTCATGTATTCATAGTTTAAAGTGCATTTTCATTTTATTTCAATTTGGG
GCGGCTTTGTTAAAGTTTTAGCCTGATGAGGATGTTGAATTATTTTTGTACTTAATCACTCTATATGGTATTGACATGCC
AAGTTAGCATGCAA

>*EguGA2ox5*　LOC104452105

ATGGATTTTGAGCCACCATTTCATGAGAATTACAAGACCCTTTTGCAGAACAAACCTGACAAATTAGATGATGAAAATGG
TGGCCGACAACCTCCACCACAGCTCATGCTTATTGAGCACTGTGAGTTACCTCTCATTGATCTGAGCCATCTACAAACTC
GAGGGTCATCAGTTGTGGGAGCAAAGTGCAAGGAGAACATTGTGGAAGCTGCACGAAAATGGGGTTTCTTTCAAATATTG

AATCATGGGGTCCCACCAGAGATATTGGAAAGCATGAAGCATGAGCAGACAAAGCTCTTCAGATTGCCTTTTCAGAAGAA
AGCTGAGCTCAGTTTCTTGAATTTGTTGCCATCGCCGTCATTACCATCCTCGCTGCCAACCAAGAGTTACCGCTGGGGAA
ACCCGCAGGCCACTTGCTTGAAGCAGCTTCCTTGGTCTGAGGCCTTCCAAATACCTCTAGCAAGCATCTCAAGAATGAAA
GAGAGTGACAATTTCAGAGTCGCCGTGGAGACCTACGGGATGACTGTTGCTAATCTTGCAGCATTGATAACTGAAATTCT
AGCCGAGGGTTTGGGCGTCAAAGGCGCCAATTACTTTAAAGAGAATTGCACGTTGAGTACTTGTTTCCTTCGGATGAACA
GATACCCTCCATGCCCGCTTGCTTCCCAAGTCTTTGGATTGATGCCTCACACGGACACCGATTTCCTCACGGTCTTGTGT
CAAGATAAGATTGGAGGGTTGCAGCTGATGGAAGGTGAGAGATGGGTTAGTGTTAAGCCTAATCCAAATGCTCTCCTCGT
CAACGTTGGCGACTTATTTGAGGCTTGGAGCAATGGCGTTTACAAGAGTGTGAAGCACAGAGTGATGGCCAGCCAAGAAG
TGGAAAGGTTCTCAATGGCGTTTTTCTATTGCCCCTTCAAACACACGTTGATCGAGAGCCCCCTCAAGCCAGCCCTCTAT
AGAAGCTTTAGCTTCGAAGAATACAAAGCGCAAGTTCACAGGGATGTTCAGGCCAATGGAGACAAAGTCGGCCTCTCTCG
GTTCCTGATTCAGTGAAATTATAGCACACCACTCTAAATGGGTACTCTTGTATAGGCATACATGTGTGCGTATAGGTGGT
CATTTTGTGGTAATTTTGCTTGCGAGTTC

>*EguGA2ox6*   LOC104442720

GGGAAAAGCAGAAACCGCCCTCTTCATGTCCCTTGTATATATAACCCAAGACATCCATCTGTCTGTCTTGCAGCCTCTAA
CCATGGACTCATCGGACCCGCCATTTCAAGAAACCTACAAGGCCCTCTTCAAGAAGGTAGCCAACAAAGGCCCGACCACG
CCCATTGTCGTCGAAGAGCGCGACCTTCCAGTGATCAACCTCGGTCGGCTGAGCCTCAGCAAGGAGGAGAGACGGGAGTG
CAAGGCGGAGATCGCGAGGGCCTCGCGGGAGTGGGGGTTCTTCCAGGTGTTGAACCATGGGATTTCACGAGAGATCCTGG
AGAAAATGAGGGAGGAACAGATCAAGGTGTTCCGAGAGCCGTTCGAGAAGAAGAGTAGAGAGGACAAGTACTTGGATTTC
TCGGCGGGGAGTTATCGATGGGGGACTCCTTCCGCAACATGCTTGCAGCAGTTGTCGTGGTCCGAGGCTTTCCATGTCCC
TTTGAGTGATATCATCTCCGGCACAAGAGGTTGCAAAAGCACCCTCAGCTCGACAATGGAACAATTTGCCGGGACTGTAT
CAAACCTAGCGCAGAAATTAGCCAAGATCTTAGCAGAGGAACTGGGTCACAAATCGACCTTCTTCATGGAGAATTGCCTG
CCGAGCACATGCTATCTGAGAATGAACAGATACCCACCATGTCCAATTTCACCGGACATGCTCGGGCTGATGCCGCACAC
CGACAGCGACTTCCTCACGATCCTGCATCAGGACGAGGTGGGGGGTCTGCAATTGGTCAAAGATGGGGAGTGGATCGCTG
TTAAACCTAATCCCGATGCTCTCATCATTAACATTGGCGACTTATTTCAGGCATGGAGCAACGATGTGTACAAGAGCGTT
CAGCACCGGGTGATGACCAACTCGAAACGGGAGCGGTACTCGACCGCGTACTTCTTCTGCCCATCGTACGACACTGTGGT
CGAGAGCTGCAACTGGCCTCCTGTCTACAGGAGCTTCAGCTTTCGAGAATTCAGACGACAGGTCCAAGAAGATGTACAAA
AGCTGGGTCACAAGATCGGGCTTCCCAGATTTCTCGTGTAAAAGCTGATGAGAATTTTGTGTCCAGATGTCGTCCTTATG
AATTGACAAGCTAGTTAAGCTAGTTCTTGTGATCATCAAGAAAGTACAGAAAAGAATGGTACTGGAAGAAAAAAGATGG
AAAAGGAAAAAAAAGAGAGAAAATACATGGACAGGTTTGTATATGTACCTTTAGAAGTTCAAAAAAATTTAGGGAGCTAG
AAGATTATTAGCTTATAGTTTTTAGCATAAGTTTGATATCGGTTTGAGAATCCAAATTCTGTCTACGTACAAGGAACTAG
GGTTTTGCAATTTAACATGTAGCTTGTTTTACAACTTTTTATTTGGATGGTATAATGCAAACTTTTGTTCTTTAA

>*EguGA2ox7*   LOC104453584

TGTGCTCTTTCGCCCTCGCCACTCCTCTCCCCACTTATAAATACGAGCGGCCACCGCCCAACCCACAACTCTCTCCACAC
GGTGTTGGAGCGATGGGCTCGATCTCAGACCCACCCCTTCTGTCCGACTTCACCCAACTCCTCCGGAGTTGCCCCACCGT
CTCCTCCTCCTCCTACTCCCCCTCCTCTCCGGCCCTGAATGCCGATGTGGTGACGCCCCGGGAGTGCGAGCTCCCGGTCA
TCGACCTGAGTGGGCTGCGCAGCGAGTGCAAGGCGGAGAGGCGGTCGTGCGCAGCGGCAATCTGCAGGGCCTCCACGGAG
TGGGGATTCTTCCAGGTGGTGAACCATGGGATCAGCAGCGAGCTGCTCGGGGAGATGAGGAGGGAGCAGGGGAAGCTGTT
CGAGACACCCTTCGAGACCAAGGCCCGCTCTGCCCTCCTCAACAACTCCTACCGCTGGGGCAATCCTGCCGCCACCTCCC
CTGCCCAATTCTCCTGGTCCGAGGCCTTCCACGTCCCCCTCACCAAAATCTCCGATCCCTCTTGCTACGGGCCGTTCATC
TCTCTCGGGGGAGTAATGATGGAGTTCGCTGCTGCCATGTCTAGGCTGGCCAGGACGCTGGCGGGGGTCCTGGCGGAGAA
CCTCAGGCATCGGGACTGCAGCGCATTCGACGAGACATGCCACGAGAGCACGTGCTTCCTCCGGCTGAACCGCTACCCCG
TGTGCCCGCTCTCGCCGGAGATGTTTGGGTTGGTGCCCCACACGGACAGCGACTTCCTGACCATACTTTACCAGGACCAA

GTGGGAGGCCTCCAGCTCCTCAAGGACTCCCGATGGGTCGCCGTCAAGCCCAACCGAGACGCTCTTATCGTCAACATCGG
AGACCTTCTTCAGGCGTGGAGCAACGACGTGTACAAGAGCGTGGAGCACAAGGTGATAACGAACGACCAGGTGGAGAGAT
ACTCTGTCGCCTACTTCCTTTGCCCTTCGTACGACTCTCCGATCGGGAGTTGCACAGAGCCCTCCGTCTACAGAAAGTTC
ACTTTCGGCGAGTACCGGAAGCAAGTCCGAGAGGACGTCAAGAGAAACGGCCACAAAGTTGGTCTCCCACGTTTCAAGCT
ACAAATGAGTGCATAAAGACAGTTCGTTTGATTGAAATATACGAAAGCCATCCTATGCGGACTTCCATCTTTTATACCAA
TGCACAATTCTGAGAAATTATTGGTTTATTAAAAGAAAATGAAGAAGGAGAAGAAGAAAAAAGGGAAGAAAAGAGACGGA
GGACAATTTTTCTAGGTGTCTTACGATGTATCTGTAAATCTATAGCATACCATCTATACTCTGCAGCCTCCAAAAAAGCA
AAGAAAGGGGCGGCACATCATGTGTAAGTCAATATGGCCTTTGTATAAGATCTTTTTGACAGACAATATTAGTACTTA

＞*EguGA2ox8*　LOC104446592

ATGGCCGGATCGAGCTCAGCCCCAACCCGTCTGTCCAACGTAATGACACCCCAGGAGTGTGAGCCCCCATTGATAGACCT
GCGTTGGCTGAGCAGTGCGTGTGAGGCAGAGCGGCAGTTGTGTGCGGAGGCCATATGCAAGGCGGCCGCAGACTGGGGAT
TCTTCCAGGTGGTGTACCATGGGATCAGCAGTGAGCTGCTTGGGAAGACGAGGAGGGAGCTGGTTAAGCTGTTTGGAGCA
CCCTTCGACACCAAGGCCCACCCTGGCCTCCTCAACAACTCCTACCGCCGGGGCACTCCCCCTGCCCCCTCCCCCGCCCG
ATTCTCCGGGCCTGAGGCCTTCCATGTCCCCTTCATGAAGATATATGACCCCTCTTGCTACGGGCCATTTACCTCTCTCG
GAGGAGTGACGAGGGAGTTGGCCCCCGCCATGTTTGGGCTAGCGAGGACACTGGCGGTGGTCCTAGCGGAGAGCCTCGGG
CATCACGGCCGCAGCACGTTCTATGAGAAATGCAACGAGAAAACGTGCTTCCTCCGGTTGAACTACTACCCAGTGTGCCC
GCTCTCGCCGCAGACGTACCAGCCGGTGCCCCACGTGGACAGTGACTTCCTGACCGTTCTCTATCCGGACCAGGTAGAAG
GCATCCGGCTCCACATGGACTCCCGATGGGTCGTGGTCAAGCCCAACCGTGACGCTCTCATTGTCAGCACTGGAGACCTT
CTTCAGGCGTGGAGCAATGACGTGTACAGGAGTGTGGAGCAAAAGGTGCGGACAAAGAGAGAAGTTGAGAGATACTCCGT
TGCTTTCTCCCTCTGCCCTTCGCCCGACTCTCAGATCGGGAGCTGCAGAAAGCCCTCCGTCTACAGAAAGTTCACTTTTG
GTGAATACCGGGAGCAAGGCCGTAGATATGGTCTTCCGCATTTCAGGCTAACGGAGAGGGTTGACGTCTCCACAGACTTG
GCGCTCTACGAAGACCCGTGGAAGATCAGGAAAAAGCTAACGGGAAGCGACCTCAGCCCTTTGTCGCGGCTCCTGCTTCC
AGCGGGCTGCCTGCAGACCCACGTGTTCCCGCAGATGGACAAGAAGATGCTGAGGCAGGTCAACAGCGAGGAGGGGATGC
CGGTCGTCGGGAAAGACGTGGCCACAGGCCGCAAGCACCAGTTTGTCTTCCGCTGCTGGAAGTCGACCGGGAGCTACGTG
CTGAACGGTGGCTGGACCAAGGAGTTTGTCGAGGAGAAAAGGTTGAAGGTCGGGGACGAGATCGAGATGGTCTGGGTCAT
GTCCTCTCGCATGTTCCACTTTAAGGTCCTTCACAGGGCCGCTGCTTGA

＞*EguGA2ox9*　LOC104446596

ATGATGATGCCCCAGGAGTGTGAGCTCCCAGTGATAGACCTGAGTCAGCAGGGTGCAGAGGAGCGCATATTCTGGGCCGT
CAAAGAATATGGACTCGTCCAGGTGGTGGACCATGGGATCAGGAGTGAGCTGCTTGCGAAGACGACCGGGGAGCTGGTTA
AGCTGTTTGGAACACCCGTCGAGACCAAGGCCCACCGGGGCCTCCTCAACAACTCCTACCACTGGGGCACTCCCATCGCC
ACCTCCTCCACTCAATTCTCCTGGCCTGAGGCCTTCCATGTCCCCTTCATGAAGATACATGACCCCTCTTGCTACGGGCC
ATTCATCTCTCTCAGAGAACTTATGATGGAGTTGGGCCCCGCCATGTCCAGGCTAGCCAGGATGCTGGCCGGGGTCCTAG
CAAAGAAACTCGGGCATCAGGGCTGCAGCATGTTCAACGACATATGCGATGACAGCACGTGCTTCCTCCGGCTGAACCAC
TACCCAGTGTGCCCGCTCTCGCCGGAGACGTTCCGGCCGGTGCCGCACGTGGACAGTGACTTCCTGACCGTTCTCTACCC
GGACCAGGTAGGAGGCATCCGGCTCCGCAAGGACTCCCGATGGGTCGTGGTCAGGCCCAACCGTGGCGCTCTCGTCGTCA
GCATTGGAGACCTTCTTCAGGCGTGGAGCAATGACGTGTACAGGAGTATGGAGCGAAAGGTGCAGATAAACAGCGAGGTG
GAGAGATACTCCGTTGCTTTCTCCCTCTGCCCTTCGCCTGACTCTCAGATCGGGAACTACAGAAAGCCCTCCGTCTACAG
AAAGTTCACTTTTGGTGAATACCGGGAGAAAGGCCGTATATATGGTCTTCCGCATTTCAGGCAAACGGAGAGGGTTGACG
TCTCCACAGAGTTGGTGCTCCAAAACGACCCGTGGATGATCATGAAGGAGCTAACGATAAGCGACCTCAACGACTTGTCG
TGGCTCCTGCTTCCGCTGGGCTGCCTGAAGGCCCACGTGTTCCCGCAGATGGACGAGGAGATGCTGAGGCAGGTCGTGAG
CAAGGAGGGGATGCAGGTCGTTGGGATGGACGTGGACACAGGCTGCAAGCACTGGTTCGTGTTCCGCCGCTGGGGGTCGT
CCAGGAGCTACGTGCTGAACGGTGGCTGGACCAAGGAGTTTGTCGAGAAGAAAAGGTTGAAGGTCGGGGACCAGATCGGG

ATGGTCTGGTTCATGTCCTCTCGCATGTTCTACTTTAAGGTCTTTCACAGGGCCGCTGCTTGA

＞*EguGA2ox10* LOC104430264

ATGATGAAACCCCAGGAGTATCAGCTCCCAGTGATAGACCTGAGTCAGCCGTGTGCAAAGGAGAACATATTCCGGGCCGC
CAAAAAATATGGACTCCTCCAGGTGGTGAACCATGGGATCAGTAGGGAGCTGCTTGGGAATGTGATGAGGGAGGCGGTTA
AGCTGTTCGAGACACCCCTTGAGACCAGGGCCCACTCTGGCCTCCTCAACAACTCCTCCCCCTGGGGCACTTCCCCTGCC
CAGTTCTCCCACCATGTCCCCCTCGTGAAGATTTATGACCCCTCTTGCCACGGCCAGTTCAACTCTCTCGGAGGAGTGAT
GAGGGAGTTGGCCCCCGCCATGTTTGGGCTAGCCAGGACGCTGGCGGGGGTCCTAGAGGAGAGCCTCGGGCATCAGGGCC
GCAGCACGTTGAACGAGAAATGCGACGAGAACACATGCTTCCTCCGGCTGAACCACTCCCCAGTGTGCCCACTCTCGCCG
GAGAGGTTCTGGCCGTTGCCCCACGCGGACAGTGACTTCCTGACCATTCTCTACCAGGACCAGGTAGGAGGCCTCCGGCT
CCTCAAGGACTCCCAATTCGTCGCGGTCAAGCCCAACCGTGACGCTCTCATCGTCAGCATTGGAGACCTTCTTCAGGCAT
GGAGCAATGACGTGTGCAGGAGTGTGGAGCAAAAGGTGGAGAAAAACAGGGAGGTGGAAAGATACTCCGTTGCTTTCTCC
CTCTGCCCTTCGTCCGGCTCTCAGATCGGGCGCTGCAGAAAGCCCTCCGTCTACAGAAAGTTCACTTGTGGTGAATACCG
GAAGCAAGTTCAAGAGGACATCAAAAGAACCTGCCGTAAAGTGGGTCTTCCACGTTTCAGGCAAATAGAGAGGAATATAT
CATCTGACGTCTCCACGGAGTTGGTGTTCTACGACGACCCGTGGAAGATCAGGAAGGAGCTAACGAAAAGCGACCTCGAC
CACTTGTCGCGGCTCCTGCTTCGGCGGGACTGCGTGAAGGCCCATGTGCTCCGGTGGATGAAGAAGGAGATGGTGGGCAA
GGTCGAGAGCAAGGAGGGGATGGAGGTCGATGTGATAAACTAG

＞*EguGA2ox11* LOC104430763

ATGATGACACCCCAGGAGTATCAGCTCCCAGTGATAGACCTGAGTCAGCCGCGTGCAAAGGAGAACATATTCCGGGCCGC
CAAAAAATATGGACTCCTCCAGGTGGTGAACCATGGGATCAGCAGGGAGCTGCTTGGGAATGTGATGAGGGAGGCGGTTA
AGCTGTTCGAGACACCCCTTGAGACCAGGGCCCACTCTGGCCTCCTCAACAACTCCTCCCCCTGGGGCACTTCCCCTGCC
CAGTTCTCCCACCAGGTCCCCCTCGTGAAGATTTATGACCCCTCTTGCCACGGCCAATTCACCTCTCTCGGAGGAGTGAT
GAGGGAGTTGGCCCCCGCCATGTTTGGGCTAGCCAGGACGCTGGCGGGGGTCCTAGAGGAGAGCCTCGGGCATCAGGGCC
ACAGCACTTTGAACGAGAAATGCAACGAGAACACGTGCTTCCTCCGGCTAAACCACTACCCGGTGTGCCCGCTCTCGCCA
GAGAGGTTCTGGCCGTTGCCCCACGCGGACAGTGACTTCCTGACCATTCTCTACCAGGACCAGGCAGGAGGCCTCCGGCT
CCTCAAGGACTCCCGATTCTTCGCAGTCAAGCCCAACCGTGACGCTCTCATCGTCAGCATCGGAGACCTTCTTCAGGCGT
GGAGCAATGACGTGTACAGGAGTGTGGAGCAAAAGGTGGAGAAAAACAGGGAGGTGGAAAGATACTCCGTTGCTTTCTCC
CTCTGCCCTTCGTCCGGCTCTCAGATCGGGTTCTGCAGAAAGCCCTCCGTCTTCAGAAAGTTCACTTGTGGTGAATACCG
GAAGCAATTTCAAGAGGACATCAAAAGAACCTGCCGTAAAGTTGGTCTTCCACGTTTCAGGCAAATGGAGAGGAATATAT
CATCTGACGTCTCCACGGAGTTGGTGTTCTACGACGACCCGTGGAAGATCAGGAAGAAGCTAACGAAAAGCGACCTCGAC
CACTTGTCGCGGCTCCTGCTTCCGCGGGACTGCGTGAAGGCCCATGTGCTCCGGTGGATGAAGAAGGAGATGGTTGACCA
GGTCGAGAGCAAGAAGGGGATGGAGGTCGATGTGATAAACGAGGACACAGGTGACGAGCACCGGCTCGTGTTCCGCTACT
GGGCATCCTCGGGGTGCTACGTGCTGAATGGTGGCTGGAGCAAGCTGTTCGTCAATGGGGAGAGTTGA

＞*EguGA2ox12* LOC104432636

TCTCGTGTCATATAGTATTCGTACGAGCATTATATATTGAGTCCACCCCAAATTTTCTCGAGCCGCCCCCCCTGCCGAGC
GTTGACATGGAGCTGCAGTCGTCGTATCCTCCTCCGTTTCGCCGCCCGAGCGGCCCGGCCCCGGCCGTCGACCCCGACGA
GGGCTCCCGGACGACGAACCAGATCCAGGAGCAGGACCCGATCCCGGTCGTGGATCTCCAGCGCCTGGACGTGGAGGGCG
AGCGGCTCGGGGAGGCGTGCCGGGCGTGGGGGATATTCCGGGTGGTCAACCACGGGGTCCCTCTCGATCTGCTGACCCGG
CTCAGGGACCGCGCGAGGAGGCTCTTCGCGCTCCCCTTCGAGTCCAAGCAGGCCCTGCTCGTGGCCCCGCTGTCCTACTT
CTGGGGCACCCCGGTCCTGACACCGTCCGGGTCGGCCCTCCTGGGAGGCGCGCAGGGCGTGAACTGGGTGGAGGGCCTGA

ACGTCCCGCTCGCTCAGCTCTCGCTGTTGGGAGACGGCGGCGGCGGCGGCGGCGGCGGCGATGATGATGATGAGGTG
GTGACCGCGGTGGCGAGTTTGAGGAATCTTCTACAAGAATACGGAGTTCACTTAGCAAGAATCGCTAAGCTCTTATTTCA
AGCCATGGCTGAAGACCTTAGACTTGATCCAGCACACTCAAAGAGTTACCTATCGGAGTCGACGGGATTTGTACGCTTGT
ACCGCTACCCACATTGCTCGAAAGCCAGCAGCGACATGTCCGGTATGGAAGCCCACAGCGACAGCTCGGTGCTCTCCATC
CTGTGCCAGGATGAGGTGGGTGGGCTCGAGCTCTACAAGGACGAGCAATGGATACAAGTACAACCTGTCCCCGATGCTCT
AATCGTGAACGCTATGAGCGACGATGAGTACATAAGCGTGAAGCACAGAGTGAAGGTAAAGAAACCACAGGATCGGACCT
CGGTTTGTTACTTCGTGTTCCCAGGCCAAGACAGCGTGATCCGAAGCTCAAAGTACAGGCCTTTCACGTACGGCGACTTC
CAGTCACAAGTGCAGCAAGACGTCAAGACCCTTGGGTTCAAAGTCGGGCTCCCTCGATTCAAGCTTGATTAAAGCTCCGC
CGGTACTAAGAATCGCACATGTCTCGCGAGGCTCTCACGTTACAAACAGTTTTAAAAAAGAAAAAAAGACTCTGACAAGG
CAAAAGAGAGAAGAAAGAAGGTGGAGATGCGACTAATAGCTTCGACTTGTCGGGTTAATGTCATAATCAAGAGTAGATTT
TTGTTTTGGAATCTCTCATTCAACATGAATGGTTTCTATTACTCGTTATTGATGGAAGGGATCTGGTCATATAATCGTTT
ATCA

＞*EguGA2ox13*   m64168e_220610_021218/44762062/ccs

GGGGAGCCGCCCCCCTGCCGAGCGTTGACATGGAGCTGCAGTCGTCGTATCCTCCTCCGTTTCGCCGCCCGAGCGGCCGG
CCCCGGCCGTCGACCCCGACGAGGGCTCCGGACGACGAACCAGATCCAGGAGCAGGACCCGATCCCGGTCGTGGATCTCC
AGCGCCTGGACGTGGAGGGCGAGCGGCTCGGGGAGGCGTGCCGGGCGTGGGGGATATTCCGGGTGGTCAACCACGGGGTC
CCTCTCGATCTGCTGACCCGGCTCAGGGACCGCGCGAGGAGGCTCTTCGCGCTCCCCTTCGAGTCCAAGCAGGCCCTGCT
CGTGGCCCCGCTGTCCTACTTCTGGGGCACCCCGGTCCTGACGCCGTCCGGGTCGGCCCTCCTGGGAGGCGCGCAGGGCG
TGAACTGGGTGGAGGGCCTGAACGTCCCGCTCGCTCAGCTCTCGCTGTTGGGAGACGGTGGCGCGGCGGCGGCGGCGGCG
GCGATGATGATGATGAGGTGGTCACCGCGGTGGCGAGTTTGAGGAATCTTCTACAAGAATACGGAGTTCACTTAGCAAGA
ATCGCTAAGCTCTTATTTCAAGCCATGGCTGAAGACCTTAGACTTGATCCAGCACACTCAAAGAGTTACCTATCGGAGTC
GACGGGATTTGTACGCTTGTACCGCTATCCACATTGCTCGAAAGCCAGCAGCGACATGTCCGGTATGGAAGCCCACAGCG
ACAGCTCGGTGCTCTCCATCCTGTGCCAGGATGAGGTGGGTGGGCTCGAGCTCTACAAGGACGAGCAATGGATACAAGTA
CAACCTGTCCCCGATGCTCTAATCGTGAACGTAGGCGATATGTTGCAGCTATGAGCGACGATGAGTACATAAGCGTGAAG
CACAGAGTGAAGGTAAAGAAACCACAGGATCGGACCTCGGTTTGTTACTTCGTGTTCCCAGGCCAAGACAGCGTGATCCG
AAGCTCAAAGTACAGGCCTTTCACGTACGCCGATTTCAGTCACAAGTGCAGCAAACGTCAGGACCCTGGGTTCAAAGTCG
GGCTCCCTCGATTCAAGCTTGATTAAAGCTCCGCCGTACTAAGAATCGTACATGTCTCGCGAGGCTCTCACGTTACAAAC
AGTTTAAAAAAAAAAGAGACTGTGACAAGGCAAAAGAGAGAAGAAAGAAGGTGGAGATGCAACTAACAGCTTCGACTTGT
CGGGTTAATGTCATAATCAAGAGTAGATTTTTGTTTTGAATCTCTCATTCAACATGAATGGTTTCTATTACTCGTTATTG
ATGGAAGGGATCTGGTCAT

＞*EguGA3ox1*   m64168e_220610_021218/125240890/ccs

GGGAAGTGACAAGTACATTCGCTTTACTCTGTCCATTTGTAAACTGTATCTCAACCTATAACCTGACATCGAACACCCTC
CCGGTTTTCTCTGCAAGTACTCTCTCCCTATTTCTCTCTCTCCCCGCTATGCTTTCCAAACTCTCAGATGCCTTCAGAGC
ATATCCTGTCGAACTCCACCATAAACAACTCGACTTTAGTTCAGCCGAAGAGTTGCCCGACTCATATGCATGGACAGAAC
TTGGTGAGTACCCCTCTTCTCACTGTCCCAGCAAAGAGAGTTTGCCGATCATCGATCTCAATGACCCAAATGCGCTAAAG
CTAATAGGCCATGCATGCAAAAGTTGGGGTGTATATCAAGTCAACAAACCATGGCATACCAGAGGAGCTTCTTCATGATG
TTGAGCGTGCAGCCAAGAATCTTTTCTCTCTGCCGGTGCAACAAAAGCTCAAGGCATCTCGTTCCCCAGAAGGCATCTCG
GTTATGGAGTTGCAAAAATTTCTGCCTTTTTCCCTAAGCAAATGTGGTCTGAGGGTTTCACCATTATGGGGTCACCAATG
GAACATGCTCGCCGACTTTGGCCTCAAGATTACAGCCTATTCTGTGATGTAATTGAAAAGTACCAGAAAGAGATGAAACA
GCTAGCAGAAAGGTTGATGTGGCTTATGCTTGGCTCTTTGGGGTTGACAAAGGAAGACATCAAATGGAGCAGCCCTAAAG
GAGAATTCACAGGGGCAGTTGCTGCTTTGCAATTGAATTCTTACCCTGCTTGCCCAGACCCAGACAGGGCAATGGGTCTT
GCGCTGCTCACACAGACTCTACACTCCTCACAATTCTTCATCAAAATAACACAAGTGGCTTGCAAGTGCTTAAAGAAGGC

AGTGGATGGATCACAGTACCTCCACTTTCCGGTGTACTTGTTGTTAATGTGGGGGACCCTTTCACATATTATCAAATGGC
TTATACCCAAGCGTCCTCCATCGAGCAATGGTTAACCGAACAGAGCACCGGTTATCAGTTGCCTATTTATATGGACCGCC
GAGTTGGGTTCAAGTCTCACCTCTACCAGAACTAATAGACTCTAACCATCCTCCACTTTACAAGCCAGTAACTTGGAGGC
GAGTACCTTGGGACCAAGGCAAAACATTTCAACAAAGCACTGTCATTAGTTCGGCTATGTGCTCTCGTAAATGAACTACC
TGATGTAAGTGATAGTATCGTGAAAAGTCAGCTAAAGTTTTCTAGCTATTGCCCCTCAAAGTGTGTAGTGGTCGGCAAGA
TCAGAGGAACTGAATTCCTGAGGATTAAAGTGTGCAAGTCAAATGACGAACCAGATGAACTCCCATGAGAATGAACCAAA
GGATAAATTGTGTCAATCATAAAAGCGAAAGACAGCTTTTTGCCCATTTGAATATTTAGGTGAAGGACAAACAAAAGGAC
AGCTTTTTGCCCAGATGTGAAGGATGATTTTCCTCCAACGAAAATGACGAAAATCAAGGTAAAAATGGTAGGACTAATTA
TTTATCAAATGAAGCTTGGAAGATGACAAGGGACAAATGAAGACAATTACAGCTATTTCTTGCAAGGGGAAATTCGAACA
ATGACATTTTTTAAAGCAGAAGTTAACTGAGAATGTAAGGCCATGACATTTGACAGCAAGAGTAACAGATCTCTCTCTCT
CTCTCTCCTCTCTCTCTCTCTCTCTCTCTCATGCAGGCATGTGGGCACACACACGCGACACCACACACACAAAGATAATG
CGCATACGCTTGTGGCAAAGCAGTACATTAATATGTGTCAATCTATCAACCCTATCATGGCCTGGCAAAGATTTGCAAGT
TGAAAATGATATTGCCCTTGAAGCCATGTGCAATATTTTGCATGTTGTCTATTAAAGAGATCATCAGGGAGTTGTTT

＞*EguPP2A-1*　m64168e_220610_021218/110757718/ccs

GGGATTCGCATTCGCATTCGAATTTCATTTCGGTCTCTCTCTCTCTCTCTCTCTCACATGCAATTTCATCGGTCGAAACA
TCTGCGCGACATCCAAACTGCGAAGGCGAAGGCGGAAGGCGGAAGGCGGAAGGCGCAGGTGAATTCGCGGAGCTGAGTGA
TGATTGAAAGATCGCGACCCGCTCGATCGCTCGGAGATCCGCTTCTCGCCTCGTTCCGATTCGGAATCCCGATCCCGGCG
CCCGGCGAGCCGTTCCCTTCGACGGGAGCCGAGTGAGAGTGGGGGGAGAAACCGCCTCCCGTCGACGATCGCGCGGTTGT
TTCCGCCCCCCGCCTGCCATTTGCGATCTGCGGGAGCGGTACGAGGGACGAGGGACGAGGAGTGGTGGGTCGGGGCGCCT
GGGAAATATGCCGTCGCACGCGGATCTGGACCACCAGATCGAGCAGCTGATGGAGTGCAAGCCGCTGACGGAGGCGGAGG
TGAAGACGCTCTGCGATCAGGCGAGGGCGATCCTCGTGGAGGAGTGGAACGTGCAGCCGGTGAAGTGCCCGGTCACGGTT
TGCGGCGACATTCACGGCCAGTTCTACGATCTCATCGAGCTCTTTAGGATAGGCGGCAACGCGCCTGATACGAATTATCT
GTTTATGGGAGATTATGTTGATCGGGGGTACTACTCAGTGGAGACTGTTACGCTTTTAGTGGCTTTGAAAGTTCGCTATA
GAGATAGAATCACTATCCTTAGGGGAAATCATGAAAGTCGGCAAATAACACAAGTGTATGGTTTCTATGATGAATGCTTG
AGAAAGTATGGAAATGCCAATGTCTGGAAATTTTTCACCGACTTGTTCGATTATCTTCCACTAACTGCTCTTATTGAGAG
TCAGATCTTTTGTTTGCATGGAGGTCTATCACCCTCTTTGGATACACTAGACAATATCCGAGCCCTGGACCGTATACAAG
AGGTTCCTCATGAAGGACCAATGTGCGACCTCTTGTGGTCTGATCCAGACGATCGCTGTGGCTGGGGAATATCTCCACGT
GGTGCTGGTTATACCTTTGGTCAGGACATTGCTGCTAATTTTAACCACACCAATGGTCTCACTCTGATATCGAGAGCCCA
TCAGCTTGTCATGGAAGGATACAATTGGTGTCAGGAAAAAAATGTGGTCACTGTATTTAGTGCTCCCAATTATTGTTATC
GGTGTGGAAATATGGCTGCAATTCTTGAGATTGAGAGAATATGGATCAGAATTTCCTTCAATTTGAACCAGCACCACGTC
AAATTGAACCGGACACAACGCGCAAGACTCCTGATTATTTCTTGTGATTCAAGAAATGTACTTGCTGCTTGTAATTCAGG
TTTGACGCCATCTTGCTGTTGGTATGTCTTATGGAAAAAGAATTTCATCTTTAAGATCAGACTTCAGAGTGACGAGCAAT
GCCATTCTTCTGTTTGGAGCTTGTCTTGCCGCCTTGCAGCTTGTCTTTGTTGAGATCAAGGAACTTGAAGAACCAGATGG
AAGGCATCGGCGTTCTTGTTTGTCCTGTATATTCATCGAAAAATGGGGGTGGCAATAATAAGGTCTAATCTCGTTCTTCA
ACCCGAGTAGAAAATTTTTGAAGCTGTTGAAAGTTTTCCATTTTCAAATTTAGCAGACCGAAGAGCTGTGATGAGTTTAC
TCGGATAGAGAGCATCATTGGCACAAGGTTCTTGAAAGTTTGTTTTGCGGGGTGGATGAGCCGTGAAGTGGAAATTGTTT
TTGGTTCTTCGTCTGTAACACTGCTTATGAACTATTTCGGAGCATTTCTTCCTTCATGCTCGAAGTAAAGTCCATTAGGA
TTCTTTTCGCTGGATAAACGTCTTCTATGAATTGACTGCAAGGAATCTGCTTGCACGGAAGCTTCATATATTAAGATGTT
TGGTAACGTGGCAGGAAAAACTTGTTTCTGTTTTTTGGTTCTCCAGAACAGAAATAGAATAGAAACATGTTCATT

＞*EguPP2AA3*　m64168e_220610_021218/39321722/ccs

GGGGTCGCAGGACCAGAGAAGCAGGGTGCCGTAAGTCCTTCCTAGTTTTGTTCGCTGTATTGGGGCGAGACAGATCTGCA
TAGCTTCTGGAGCTGCTACTTACAGCTGAGTTACCATGTCTATGGTTGACGAGCCTCTTTATCCAATAGCGGTTCTCATA

GATGAGCTCAAGAATGATGACATCCAGTTACGATTGAATTCTATTCGGAGACTATCGACAATTGCCCGTGCTCTCGGAGA
AGAAAGGACGCGGAAGGAATTAATTCCTTTTTTAAGTGAGAACAATGACGACGACGACGAGGTGCTTCTTGCAATGGCGG
AAGAACTGGGTGTGTTTATTCCTTATGTTGGTGGAGTCGAGCATGCCAATGTCCTGCTTCCACCATTGGAGACCCTCTGT
GCTGTTGAGGAAACTTGTGTTAGGGACAAAGCAGTGGAATCCTTATGTAGAATTGGATCTCAAATGAGGGAGAGTGATTT
GGTCGACCATTTCATTCCTTTGGTTAAGAGGTTGGCGGCCGGTGAGTGGTTTACTGCTCGAGTTTCTGCATGTGGGCTGT
TTCATATTGTGTATCCTAGTGCCCCGGACATGTTGAAGACAGAGTTGCGTTCGATATACAACCAGCTATGCCAAGATGAC
ATGCCCATGGTTAGGAGGTCCGCTGCATCGAATCTGGGAAAATTTGCTGCGACTGTTGAATACGCTCATCTGAAGACTGA
TATTATATCGATATTCGAGGACCTCACACAAGATGATCAGGATTCTGTTCGATTATTGGCCGTTGAGGGTTGTGCTGCTC
TTGGGAAATTGTTGGAGGCCCAAGATTGTGTGCAACATATACTCCCCGTTATAGTTAATTTCTCTCAGGATAAGTCTTGG
CGAGTGCGCTACATGGTGGCTAATCAATTGTATGAGCTTTGCGAAGCTGTGGGACCAGAGCCTACGAGGACGGACTTGGT
CCCTGCATATGTGCGTTTACTTCGGGATAATGAGGCTGAAGTACGTATAGCAGCTGCTGGAAAAGTTACCAAGTTTTGTC
GGATTCTGAACCCAGAACTTTCCATTCTACATATACTCCCATGTGTAAAGGAACTGTCGTCGGATTCTTCCCAACATGTT
CGGTCAGCTTTAGCCTCAGTTATAATGGGAATGGCACCAGTCCTAGGGAAGGATGCTACCATTGAGCAGCTTCTTCCAAT
ATTTCTTTCCCTTCTGAAAGATGAATTTCCTGATGTGCGTCTCAACATTATTAGCAAGCTTGATCAAGTCAATCAGGTTA
TTGGTATCGATCTTTTGTCACAATCGTTATTACCAGCAATCGTAGAGCTTGCCGAGGACAGACATTGGAGGGTCCGGCTT
GCCATAATAGAGTACATACCTCTATTGGCCAGTCAATTGGGTGTAGGGTTTTTGATGATAAGCTTGGCTCTCTTTGCAT
GCAATGGTTGCAAGATAAGGTTTACTCAATTCGTGATGCAGCGGCAAACAACTTGAAGCGCCTCGCCGAAGAATTCGGTC
CTGACTGGGCAATGCAGCACATAATTCCCCAGACATTGGAGATGATAAGTAATCCACACTACTTATACAGAATGACAATT
CTGCGTGCAATTTCTCTTCTTGCCCCTGTGTTGGGGCCAGATATCACTTGCTCGAAGCTGCTGCCGGCGGTGGTTAATGC
ATCGAAAGACAGGGTGCCGAATATCAAGTTTAATGTGGCAAAAGTGCTTCAGTCCCTCATGCCAATAGTTGACCAGTCTA
CGGTGGAGAAAATGATTCGGCCCTGCTTAGTTGAGCTTAGCGAGGATCCTGATGTTGATGTCCGCTTTTTTGCCAATCAA
GCTCTTCAGGCTATTGACCAGGTTATGATGACTAGCTAGAAATGCTACTCTTGATAGATTCAGGTTGACGCATGTATGTA
AGCCAGTGTTTGATGTCTCTCTGGAATTGCTGTTACGTCACATTGGATTAATATACTTCTCACAGTTTGCGTGAGGTTTT
AACATTGGATCCTTGATTTCTAATTCCGTTGAAGGAGTAGCTTTTGTACTGTTAAGTCAGATTAGAATATGTTCTGATTG
AGGTT

2. Arabidopsis sequences

>AtCPS

TCATCATCTCTTCTTCACTAAATACTTAGACAGAGAAAACAGAGCTTTTTAAAGCCATGTCTCTTCAGTA
TCATGTTCTAAACTCCATTCCAAGTACAACCTTTCTCAGTTCTACTAAAACAACAATATCTTCTTCTTTC
CTTACCATCTCAGGATCTCCTCTCAATGTCGCTAGAGACAAATCCAGAAGCGGTTCCATACATTGTTCAA
AGCTTCGAACTCAAGAATACATTAATTCTCAAGAGGTTCAACATGATTTGCCTCTAATACATGAGTGGCA
ACAGCTTCAAGGAGAAGATGCTCCTCAGATTAGTGTTGGAAGTAATAGTAATGCATTCAAAGAAGCAGTG
AAGAGTGTGAAAACGATCTTGAGAAACCTAACGGACGGGGAAATTACGATATCGGCTTACGATACAGCTT
GGGTTGCATTGATCGATGCCGGAGATAAAACTCCGGCGTTTCCCTCCGCCGTGAAATGGATCGCCGAGAA
CCAACTTTCCGATGGTTCTTGGGGAGATGCGTATCTCTTCTCTTATCATGATCGTCTCATCAATACCCTT
GCATGCGTCGTTGCTCTAAGATCATGGAATCTCTTTCCTCATCAATGCAACAAAGGAATCACGTTTTTCC
GGGAAAATATTGGGAAGCTAGAAGACGAAAATGATGAGCATATGCCAATCGGATTCGAAGTAGCATTCCC
ATCGTTGCTTGAGATAGCTCGAGGAATAAACATTGATGTACCGTACGATTCTCCGGTCTTAAAAGATATA
TACGCCAAGAAAGAGCTAAAGCTTACAAGGATACCAAAAGAGATAATGCACAAGATACCAACAACATTGT
TGCATAGTTTGGAGGGGATGCGTGATTTAGATTGGGAAAAGCTCTTGAAACTTCAATCTCAAGACGGATC
TTTCCTCTTCTCTCCTTCCTCTACCGCTTTTGCATTCATGCAGACCCGAGACAGTAACTGCCTCGAGTAT
TTGCGAAATGCCGTCAAACGTTTCAATGGAGGAGTTCCCAATGTCTTTCCCGTGGATCTTTTCGAGCACA
TATGGATAGTGGATCGGTTACAACGTTTAGGGATATCGAGATACTTTGAAGAAGAGATTAAAGAGTGTCT
TGACTATGTCCACAGATATTGGACCGACAATGGCATATGTTGGGCTAGATGTTCCCATGTCCAAGACATC

GATGATACAGCCATGGCATTTAGGCTCTTAAGACAACATGGATACCAAGTGTCCGCAGATGTATTCAAGA
ACTTTGAGAAAGAGGGAGAGTTTTTCTGCTTTGTGGGGCAATCAAACCAAGCAGTAACCGGTATGTTCAA
CCTATACCGGGCATCACAATTGGCGTTTCCAAGGGAAGAGATATTGAAAAACGCCAAAGAGTTTTCTTAT
AATTATCTGCTAGAAAAACGGGAGAGAGAGGAGTTGATTGATAAGTGGATTATAATGAAAGACTTACCTG
GCGAGATTGGGTTTGCGTTAGAGATTCCATGGTACGCAAGCTTGCCTCGAGTAGAGACGAGATTCTATAT
TGATCAATATGGTGGAGAAAACGACGTTTGGATTGGCAAGACTCTTTATAGGATGCCATACGTGAACAAT
AATGGATATCTGGAATTAGCAAAACAAGATTACAACAATTGCCAAGCTCAGCATCAGCTCGAATGGGACA
TATTCCAAAAGTGGTATGAAGAAAATAGGTTAAGTGAGTGGGGTGTGCGCAGAAGTGAGCTTCTCGAGTG
TTACTACTTAGCGGCTGCAACTATATTTGAATCAGAAAGGTCACATGAGAGAATGGTTTGGGCTAAGTCA
AGTGTATTGGTTAAAGCCATTTCTTCTTCTTTTGGGGAATCCTCTGACTCCAGAAGAAGCTTCTCCGATC
AGTTTCATGAATACATTGCCAATGCTCGACGAAGTGATCATCACTTTAATGACAGGAACATGAGATTGGA
CCGACCAGGATCGGTTCAGGCCAGTCGGCTTGCCGGAGTGTTAATCGGGACTTTGAATCAAATGTCTTTT
GACCTTTTCATGTCTCATGGCCGTGACGTTAACAATCTCCTCTATCTATCGTGGGGAGATTGGATGGAAA
AATGGAAACTATATGGAGATGAAGGAGAAGGAGAGCTCATGGTGAAGATGATAATTCTAATGAAGAACAA
TGACCTAACTAACTTCTTCACCCACACTCACTTCGTTCGTCTCGCGGAAATCATCAATCGAATCTGTCTT
CCTCGCCAATACTTAAAGGCAAGGAGAAACGATGAGAAGGAGAAGACAATAAAGAGTATGGAGAAGGAGA
TGGGGAAAATGGTTGAGTTAGCATTGTCGGAGAGTGACACATTTCGTGACGTCAGCATCACGTTTCTTGA
TGTAGCAAAAGCATTTTACTACTTTGCTTTATGTGGCGATCATCTCCAAACTCACATCTCCAAAGTCTTG
TTTCAAAAAGTCTAGTAACCTCATCATCATCATCGATCCATTAACAATCAGTGGATCGATGTATCCATAG
ATGCGTGAATAATATTTCATGTAGAGAAGGAGAACAAATTAGATCATGTAGGGTTATCAAAAAAAAAATA
AAAAAATTAGATCATGTACACATTAATCAAACCTTTGAATATCTATTTGCCTCCATTTAATAGATATTCA
TATATCTATGTAAGG

>AtKS

AGATATTTTCGGGTCCTACGGTCGTCGTCAGTCGTCAAGGCTAATTCGTCGCGAGTTGCTACGACGCCGT
TTCGGTTGCTTCTGGTTTCTTTATGTCTATCAACCTTCGCTCCTCCGGTTGTTCGTCTCCGATCTCAGCT
ACTTTGGAACGGAGATTGGACTCAGAAGTACAGACAAGAGCTAACAATGTGAGCTTTGAGCAAACAAAGG
AGAAGATTAGGAAGATGTTGGAGAAAGTGGAGCTTTCTGTTTCGGCCTACGATACTAGTTGGGTAGCAAT
GGTTCCATCACCGAGCTCCCAAAATGCTCCACTTTTCCCACAGTGTGTGAAATGGTTATTGGATAATCAA
CATGAAGATGGATCTTGGGGACTTGATAACCATGACCATCAATCTCTTAAGAAGGATGTGTTATCATCTA
CACTGGCTAGTATCCTCGCGTTAAAGAAGTGGGGAATTGGTGAAAGACAAATAAACAAGGGTCTCCAGTT
TATTGAGCTGAATTCTGCATTAGTCACTGATGAAACCATACAGAAACCAACAGGGTTTGATATTATATTT
CCTGGGATGATTAAATATGCTAGAGATTTGAATCTGACGATTCCATTGGGCTCAGAAGTGGTGGATGACA
TGATACGAAAAAGAGATCTGGATCTTAAATGTGATAGTGAAAAGTTTTCAAAGGGAAGAGAAGCATATCT
GGCCTATGTTTTAGAGGGGACAAGAAACCTAAAAGATTGGGATTTGATAGTCAAATATCAAAGGAAAAAT
GGGTCACTGTTTGATTCTCCAGCCACAACAGCAGCTGCTTTTACTCAGTTTGGGAATGATGGTTGTCTCC
GTTATCTCTGTTCTCTCCTTCAGAAATTCGAGGCTGCAGTTCCTTCAGTTTATCCATTTGATCAATATGC
ACGCCTTAGTATAATTGTCACTCTTGAAAGCTTAGGAATTGATAGAGATTTCAAAACCGAAATCAAAAGC
ATATTGGATGAAACCTATAGATATTGGCTTCGTGGGGATGAAGAAATATGTTTGGACTTGGCCACTTGTG
CTTTGGCTTTTCGATTATTGCTTGCTCATGGCTATGATGTGTCTTACGATCCGCTAAAACCATTTGCAGA
AGAATCTGGTTTCTCTGATACTTTGGAAGGATATGTTAAGAATACGTTTTCTGTGTTAGAATTATTTAAG
GCTGCTCAAAGTTATCCACATGAATCAGCTTTGAAGAAGCAGTGTTGTTGGACTAAACAATATCTGGAGA
TGGAATTGTCCAGCTGGGTTAAGACCTCTGTTCGAGATAAATACCTCAAGAAAGAGGTCGAGGATGCTCT
TGCTTTTCCCTCCTATGCAAGCCTAGAAAGATCAGATCACAGGAGAAAAATACTCAATGGTTCTGCTGTG
GAAAACACCAGAGTTACAAAAACCTCATATCGTTTGCACAATATTTGCACCTCTGATATCCTGAAGTTAG

CTGTGGATGACTTCAATTTCTGCCAGTCCATACACCGTGAAGAAATGGAACGTCTTGATAGGTGGATTGT
GGAGAATAGATTGCAGGAACTGAAATTTGCCAGACAGAAGCTGGCTTACTGTTATTTCTCTGGGGCTGCA
ACTTTATTTTCTCCTGAACTATCTGATGCTCGTATATCGTGGGCCAAAGGTGGAGTACTTACAACGGTTG
TAGACGACTTCTTTGATGTTGGAGGGTCCAAAGAAGAACTGGAAAACCTCATACACTTGGTCGAAAAGTG
GGATTTGAACGGTGTTCCTGAGTACAGCTCAGAACATGTTGAGATCATATTCTCAGTTCTAAGGGACACC
ATTCTCGAAACAGGAGACAAAGCATTCACCTATCAAGGACGCAATGTGACACACCACATTGTGAAAATTT
GGTTGGATCTGCTCAAGTCTATGTTGAGAGAAGCCGAGTGGTCCAGTGACAAGTCAACACCAAGCTTGGA
GGATTACATGGAAAATGCGTACATATCATTTGCATTAGGACCAATTGTCCTCCCAGCTACCTATCTGATC
GGACCTCCACTTCCAGAGAAGACAGTCGATAGCCACCAATATAATCAGCTCTACAAGCTCGTGAGCACTA
TGGGTCGTCTTCTAAATGACATACAAGGTTTTAAGAGAGAAAGCGCGGAAGGGAAGCTGAATGCGGTTTC
ATTGCACATGAAACACGAGAGAGACAATCGCAGCAAAGAAGTGATCATAGAATCGATGAAAGGTTTAGCA
GAGAGAAAGAGGGAAGAATTGCATAAGCTAGTTTTGGAGGAGAAAGGAAGTGTGGTTCCAAGGGAATGCA
AAGAAGCGTTCTTGAAAATGAGCAAAGTGTTGAACTTATTTTACAGGAAGGACGATGGATTCACATCAAA
TGATCTGATGAGTCTTGTTAAATCAGTGATCTACGAGCCTGTTAGCTTACAGGAAGAATCTTTAACTTGA
TCCAAGTTGATCTGGCAGGTAAACTCAGTAAATGAAAATAAGACTTTGGTCTTCTTCTTTGTTGCTTCAG
AACAAGAAGAGGTTTGTTTAATTTAGAAAAACAAAGGCTGTTTCTTTTCATTCATTTATATTGAGTTCTT
TTCAAAATTTCAACAAATCTTCTAGTAACTTAGTAGATATAAGACAATATTAGAGGAAAATTGATTTTAG

>AtKO

CAAGTTTCTATGAAACGTAAAACGATGAGTTTAGTAAAAAAAAACTAGAGATTATAGTCAGAAAAACTAT
AATAATAAGAAGATAACATTAAACGGATAGATTCATGTCTCTTTTTTGAAATCCAAAATTGGGTAAAATC
CAAAAACCAAAATCTTGCCAAATGCCAATAAGAGCAAAAATCCAAACTTTCCTTTCTCTTCACTATTACA
CAATGAAATCAACACTACATTTTCACATCAAAATCACATTCCTTCCATGGCCTTCTTCTCCATGATCTCC
ATTCTCCTTGGCTTTGTTATCTCCTCCTTCATCTTCATCTTCTTCTTCAAGAAACTTCTCTCCTTCTCCA
GAAAGAACATGTCTGAAGTCTCCACTCTCCCCTCTGTTCCAGTGGTACCAGGGTTTCCTGTTATTGGGAA
CTTGCTGCAACTAAAAGAGAAGAAACCTCACAAGACTTTCACTAGATGGTCAGAGATTTATGGTCCTATT
TACTCTATAAAGATGGGTTCTTCTTCTCTTATTGTCCTCAATTCTACTGAGACTGCCAAAGAGGCCATGG
TGACGCGGTTTTCGTCTATCTCAACGAGGAAGTTGTCAAATGCGTTGACAGTCCTTACTTGTGACAAATC
TATGGTTGCTACTAGTGATTATGATGATTTCCACAAGTTGGTGAAACGGTGTCTCTTGAACGGTCTTTTG
GGTGCTAATGCACAGAAACGAAAAAGACATTACAGAGATGCACTCATTGAAAATGTGTCTTCCAAGTTGC
ATGCCCATGCTAGGGACCATCCACAAGAACCTGTAAACTTCAGAGCTATATTTGAGCATGAGCTTTTCGG
TGTAGCATTGAAGCAAGCTTTTGGGAAAGATGTGGAATCCATTTATGTTAAAGAACTCGGTGTGACTTTG
TCGAAAGACGAGATCTTCAAGGTTTTAGTACATGACATGATGGAAGGTGCAATTGATGTTGATTGGAGAG
ACTTCTTCCCATACTTGAAATGGATTCCAAATAAAAGTTTTGAAGCAAGAATCCAGCAAAAGCATAAACG
TAGACTCGCAGTGATGAATGCTCTGATTCAAGATCGACTGAAGCAGAATGGTTCAGAATCGGATGATGAT
TGCTATCTCAACTTCTTGATGTCGGAAGCGAAAACACTAACCAAGGAGCAAATTGCTATCTTGGTTTGGG
AGACGATTATCGAGACAGCTGACACTACTTTGGTTACAACTGAATGGGCCATCTATGAGCTCGCTAAGCA
TCCAAGTGTCCAAGATCGTCTGTGCAAAGAAATCCAAAATGTCTGCGGAGGAGAAAAGTTCAAAGAAGAG
CAATTGTCTCAAGTTCCTTATCTCAATGGAGTCTTTCATGAAACGCTTAGGAAATACAGTCCTGCTCCTC
TAGTTCCCATTCGCTACGCCCACGAGGATACGCAAATCGGAGGCTATCATGTCCCTGCAGGAAGTGAGAT
TGCAATAAACATATATGGATGCAACATGGATAAGAAGCGTTGGGAGAGACCAGAGGACTGGTGGCCGGAG
CGGTTTCTTGATGATGGCAAATATGAAACGTCAGATCTTCACAAGACAATGGCGTTTGGAGCGGGAAAGA
GGGTTTGTGCTGGTGCTCTTCAAGCATCTCTCATGGCAGGCATTGCTATTGGAAGATTAGTGCAAGAATT
CGAGTGGAAGCTTAGAGATGGCGAAGAAGAGAATGTGGATACATATGGCTTGACCTCTCAGAAGCTTTAT
CCTCTTATGGCTATTATCAATCCAAGGCGTTCTTAAGACAGAAATCTCTTCTTTCTTTTGTTAGTTTTTA

ACCTTGTATCAACTTTTATTTGTAACAAATGCTGTTTTGTAGTACTATTTATGTTTTACTGTGTTATATT
TTACTATCCTCTGAGATTGCATCATCTTATTGATTCTTATA

>AtKAO1

AAAAGTATAAAACTTGGGAAAGTAGGATAAAGTTAGAGACTTTTATTTGTTGGTCTTGGTCACATGTCAA
AACAGACACTTTGGTTCTATTGGTTTGGGTTTCGGTCGTCTGAGGCCCGAAAGCTGCTTCTTACTCGAAA
GTCGAAACCCTTGTGAGAGAACTTGGTGATGCCGAAGATGGTAACTCCGACGAGTACTGGAACCAGTTTA
TTGAGCCATTCGCAGAATCTGAGGGTCAAGTGGTCTCTTCTCTCTCATTGCTTTATCTTAATTAAGACTT
TCCAGGTATTGAGTAATGTAATACACGTATCTACATGAAGATGAGGATATAAATATCCATTTATGAGAAA
AGAAAAAAAGGGACAAACAAAAGTTGGCAAGTTTAACTAAGTGAAAAGTCTATGGCGGAGACAACGAGTT
GGATCCCAGTATGGTTTCCTCTGATGGTGTTGGGATGTTTTGGTCTGAATTGGTTGGTCAGGAAGGTGAA
TGTGTGGCTCTATGAGTCCAGCCTTGGGGAGAACAGGCACTATCTGCCACCAGGTGATTTGGGTTGGCCT
TTCATTGGCAACATGTTGTCTTTTCTCAGAGCTTTCAAGACTTCTGACCCTGACTCCTTCACTCGCACTT
TAATCAAAAGGTATGGACCTAAAGGTATATATAAAGCACACATGTTCGGGAACCCAAGTATAATAGTAAC
AACATCAGACACTTGCCGGCGTGTGCTGACAGACGATGATGCGTTCAAGCCAGGTTGGCCAACATCTACA
ATGGAACTCATTGGAAGGAAGTCATTCGTTGGTATCTCTTTCGAGGAACACAAGCGTCTCAGGCGTTTGA
CTGCTGCTCCAGTCAATGGCCATGAAGCCCTCTCTACTTATATACCGTACATTGAAGAAAACGTTATTAC
TGTTCTGGACAAGTGGACCAAAATGGGAGAGTTCGAGTTCTTGACTCATCTGCGTAAGCTCACCTTTAGG
ATCATCATGTACATCTTTCTCAGCTCTGAGAGTGAGAATGTAATGGATGCATTGGAACGAGAATATACAG
CTCTTAACTATGGGGTTCGAGCAATGGCAGTCAATATTCCTGGATTTGCTTATCATAGAGCACTCAAGGC
GAGGAAAACACTTGTAGCTGCCTTTCAGTCCATAGTGACTGAGCGCAGAAATCAAAGGAAGCAGAACATT
TTATCAAATAAGAAAGATATGTTAGACAATCTTCTTAATGTTAAAGATGAAGATGGAAAAACTTTGGATG
ACGAAGAGATTATCGATGTTCTTCTGATGTATCTTAATGCCGGTCATGAATCCTCTGGCCACACCATTAT
GTGGGCTACCGTTTTTCTACAAGAACACCCTGAGGTTCTACAAAGAGCAAAGGCTGAACAAGAAATGATC
CTGAAAAGTAGGCCTGAAGGTCAAAAAGGTCTATCTCTAAAAGAAACCCGGAAAATGGAATTCTTATCGC
AGGTTGTCGATGAGACACTTCGAGTCATAACATTCTCACTCACTGCTTTTCGAGAGGCAAAGACTGACGT
TGAAATGAATGGCTATTTGATCCCAAAAGGTTGGAAGGTTTTGACATGGTTTAGGGATGTCCACATCGAC
CCTGAAGTCTTTCCAGATCCAAGAAAATTTGATCCTGCTAGATGGGATAATGGTTTCGTACCAAAAGCTG
GTGCGTTCCTTCCTTTTGGTGCTGGAAGCCATCTATGCCCGGGAAATGATCTGGCTAAGCTCGAGATTTC
AATTTTTCTTCATCATTTCCTCCTCAAATATCAGGTGAAACGGAGCAACCCCGAATGTCCAGTGATGTAT
CTGCCTCATACCAGACCAACTGATAATTGCTTGGCAAGAATTAGTTATCAGTAAAACATGGTTTTCATCT
TCTCCGTTAAGTTCTAGTGAAATGCCGTCAAGTTTGGTTTATCAGTTATAGGAGTTCGTTAACTCTTAAG
TAAATCAATCATTGTCAAAAAAAAAAAGAAAAGTTAAACGGTCTTGAGACTGTCTTTTTTTTTCTCGTTCT
GAAACTTCATCAGACTTACATACTCTGTTCCATGAAACAGAGCTTTGTTGTGTTCATTTGCTATAATACA
TATCGTTTGCCAAAAAAAAAAAAAGCTTCATAGGTCT

>AtKAO2

AATCTCTGGTTCAATGGTGGTACGGAGATGGCGGAGAAGGCAAATCCGGCAAGTTCTGACACCTATTTAA
CCGGAGGGACTGAAATTTTGCATCTTTTTCTGCTCACGTAGCTCTCGCCAATGGGGGAGACGAAGCAGGG
TGGCTCAAAAAGGGTATGATATAACATCTAGAGGATACAAGAGGTGGTCAGGTGCAAAAGATTGAGTTTT
TTTTATAGGAGAGTGTTTACGACTGAAGCTATGACGGAAACGGGTTTGATTCTCATGTGGTTCCCTTTGA
TAATATTGGGATTGTTTGTTTTGAAATGGGTGCTGAAGAGAGTGAATGTTTGGATTTATGTGTCCAAACT
TGGTGAGAAAAAACACTATCTGCCACCAGGTGATTTGGGATGGCCAGTCATAGGCAACATGTGGTCCTTT
CTTAGAGCTTTCAAAACATCTGATCCTGAATCCTTCATCCAATCCTACATCACCAGGTATGGGCGTACCG

GGATTTATAAAGCACACATGTTTGGGTACCCATGTGTACTAGTAACAACACCAGAGACTTGTAGGCGAGT
TCTAACAGATGATGATGCCTTCCACATAGGTTGGCCAAAATCTACCATGAAACTCATTGGCAGGAAGTCC
TTTGTTGGTATCTCCTTTGAAGAACACAAGAGGCTCAGGCGTTTGACTTCTGCTCCTGTCAATGGCCCTG
AAGCTCTCTCTGTATACATACAGTTCATTGAAGAAACTGTTAATACTGATCTAGAAAAATGGTCCAAAAT
GGGAGAAATCGAGTTCTTATCTCATTTGCGTAAGCTTACGTTTAAGGTCATTATGTACATATTTCTCAGC
AGTGAGAGTGAGCATGTCATGGATTCATTGGAACGGGAGTATACCAACCTTAACTATGGAGTTCGAGCAA
TGGGTATTAATCTTCCTGGGTTTGCTTATCATAGAGCTTTGAAGGCAAGGAAAAAACTTGTAGCTGCCTT
TCAGTCCATAGTGACTAACCGAAGAAATCAAAGGAAGCAGAATATTTCATCCAATAGAAAAGATATGCTG
GATAATCTAATAGATGTTAAAGATGAAAACGGAAGAGTCTTAGATGATGAGGAAATTATTGACCTTTTGT
TGATGTATCTTAATGCGGGTCATGAATCTTCTGGACACCTCACTATGTGGGCTACCATTTTGATGCAAGA
ACACCCTATGATTCTGCAAAAGGCTAAGGAAGAACAAGAGAGGATCGTAAAAAAGAGAGCACCGGGACAG
AAGTTGACGCTTAAAGAGACACGTGAAATGGTGTATCTTTCTCAGGTTATTGATGAGACCCTTCGAGTAA
TTACATTCTCTCTGACGGCCTTCAGGGAAGCAAAGAGTGATGTCCAAATGGATGGCTATATAATTCCAAA
AGGCTGGAAGGTTCTGACTTGGTTTAGGAACGTCCATTTGGACCCTGAAATCTACCCGGATCCAAAGAAA
TTTGATCCTTCAAGATGGGAGGGATACACACCAAAAGCAGGCACATTCCTTCCTTTTGGTTTGGGAAGCC
ACCTATGCCCGGGAAACGATCTTGCCAAGCTCGAGATTTCCATTTTTCTTCATCATTTCCTCCTCAAATA
CCGGGTGGAAAGGAGCAATCCCGGATGTCCGGTGATGTTCTTGCCTCACAATCGACCCAAAGATAATTGC
CTAGCAAGAATCACCAGAACGATGCCATAATCATCATCTTCAAACTAGTAGACCCCTTAGCAAATATATA
AGCTTCGTCTCAGGTTTCCATTGTCTTCATCTTCTCTTAGAACTTGATCCAAACCGGTTCGGTTTGTGAA
TTTGGTTTACTAGGATGTATCAATAGTTTGAAGGATTTGTCAAAGTAATGTTGAAATTTTTCTCTGGTTT
AGCTTCAAATTTGGTTATAAAAAAGTAAAATCGAATTTCTTGAAATAAAACCTAGCTTGATTA

>AtGA20ox1

TTGATAACTCCATTACAGACTATAGTATTGTACTACTAGAAAACAAAAACAACAAAAAAAGAAGTGGACA
ACACTATACGATCGACTTAAATGCTTGCTTATATAAAGACTAAAAGGACCATTGGTTCCCGTATCTCCTC
GCAATACTACTACTCACTTTACTATAATCTCTCAAAATGGCCGTAAGTTTCGTAACAACATCTCCTGAGG
AAGAAGACAAACCGAAGCTAGGCCTTGGAAATATTCAAACTCCGTTAATCTTCAACCCTTCAATGCTTAA
CCTTCAAGCCAATATCCCAAACCAATTCATCTGGCCTGACGACGAAAAACCTTCCATCAACGTTCTCGAG
CTTGATGTTCCTCTCATCGACCTTCAAAACCTTCTCTCTGATCCATCCTCCACTTTAGATGCTTCGAGAC
TGATCTCTGAGGCCTGTAAGAAGCACGGTTTCTTCCTCGTGGTCAATCACGGCATCAGCGAGGAGCTTAT
TTCAGACGCTCATGAATACACGAGCCGCTTCTTTGATATGCCTCTCTCCGAAAAACAGAGGGTTCTTAGA
AAATCCGGTGAGAGTGTTGGCTACGCAAGCAGTTTCACCGGACGCTTCTCCACCAAGCTTCCATGGAAGG
AGACCCTTTCTTTCCGGTTTTGCGACGACATGAGCCGCTCAAAATCCGTTCAAGATTACTTCTGCGATGC
GTTGGGACATGGGTTTCAGCCATTTGGGAAGGTGTATCAAGAGTATTGTGAAGCAATGAGTTCTCTATCA
CTGAAGATCATGGAGCTTCTGGGGCTAAGTTTAGGCGTAAAACGGGACTACTTTAGAGAGTTTTTCGAAG
AAAACGATTCAATAATGAGACTGAATTACTACCCTCCATGTATAAAACCAGATCTCACACTAGGAACAGG
ACCTCATTGTGATCCAACATCTCTTACCATCCTTCACCAAGACCATGTTAATGGCCTTCAAGTCTTTGTG
GAAAATCAATGGCGCTCCATTCGTCCCAACCCCAAGGCCTTTGTGGTCAATATCGGCGATACTTTCATGG
CTCTATCGAACGATAGATACAAGAGCTGCTTGCACCGGGCGGTGGTGAACAGCGAGAGCGAGAGGAAATC
ACTTGCATTCTTCTTGTGTCCGAAAAAAGACAGAGTAGTGACGCCACCGAGAGAGCTTTTGGACAGCATC
ACATCAAGAAGATACCCTGACTTCACATGGTCTATGTTCCTTGAGTTCACTCAGAAACATTATAGAGCAG
ACATGAACACTCTCCAAGCCTTTTCAGATTGGCTCACCAAACCCATCTAAGAAATAAAATATTCATGTCT
TGTCTTGTTAGTTACTAGTATCTTCTTTATATTTCATGTATGTATATGGTAATAGGCAATAACACCTTTT
AGCATCTCATTTATAAAATCTATCCGTTAAACTAAAATACT

>AtGA20ox2

TACACTAACATGACTTGAAGCTTGCTTATATAAAGACTTAAAGGACCCTTTGTTCCCCCATCTCCTCAAC
AACACTCACTCAGAACAAGACAAAACAAAAACCCCAAAACTCTCAAGAAAAAAAAAAGAAAAGAAATGGC
GATACTATGCACAACAACATCTCCGGCAGAGAAAGAACACGAACCAAAACAAGATCTTGAAAAAGACCAA
ACTTCTCCACTAATCTTTAACCCTTCTCTTCTTAACCTCCAATCCCAAATCCCAAACCAATTCATTTGGC
CAGACGAAGAGAAACCTTCCATTGACATTCCAGAGCTCAACGTCCCGTTCATCGATCTCTCAAGCCAAGA
CTCGACTCTTGAAGCTCCTAGAGTCATCGCAGAAGCTTGCACCAAACACGGCTTCTTCCTCGTCGTCAAT
CATGGCGTCAGCGAGTCACTAATAGCGGATGCTCACCGTTTGATGGAAAGTTTCTTCGACATGCCTCTCG
CCGGCAAACAGAAAGCTCAGAGAAAACCCGGTGAGAGTTGTGGCTATGCAAGTAGCTTCACCGGCAGATT
CTCCACTAAGCTCCCATGGAAGGAGACTCTCTCTTTTCAGTTTTCCAACGATAATAGTGGCTCGAGAACC
GTTCAAGATTACTTTTCCGATACATTAGGACAAGAGTTCGAGCAGTTTGGGAAGGTGTATCAAGACTATT
GTGAAGCAATGAGTTCTCTATCACTCAAGATCATGGAGCTTCTGGGCTTAAGTTTAGGCGTAAACCGAGA
CTATTTCCGAGGATTTTTCGAAGAGAACGATTCGATAATGAGGCTCAATCATTATCCTCCATGCCAAACA
CCAGATCTCACGTTAGGTACAGGACCTCATTGTGATCCAAGTTCTTTGACCATCCTTCATCAAGACCATG
TCAATGGCCTTCAAGTCTTTGTCGACAATCAATGGCAATCCATTCGTCCCAATCCCAAGGCTTTCGTTGT
CAATATTGGTGACACTTTCATGGCTCTATCGAACGGGATATTCAAGAGCTGTTTGCATAGAGCGGTTGTG
AATAGAGAGAGCGCGAGAAAATCGATGGCGTTTTTCTTGTGTCCGAAGAAAGACAAAGTGGTGAAACCAC
CAAGTGATATTTTGGAGAAGATGAAAACAAGAAAATACCCTGACTTCACTTGGTCTATGTTCCTTGAGTT
CACTCAAAAACATTACCGAGCAGATGTGAATACTCTCGATTCCTTTTCGAATTGGGTTATTACCAACAAC
AATCCCATCTAAGAAACAAAATTATTTACTATCTCAATCTTTTGTTTTTCTTTGGTTACTTTGTGTCCTT
TGTTCTCATGGTGAAATGCATTAAATTGCATTTCAAAGTTTTAAACGTTTGTATATTGATTGTTCCAAGC
TTTAGACCAATCCCTACCGTATGAGCTCGTTCAATGAATAATTTGAATGAAAAATTCAAAGAAATTTTTC
TTCATCTTTGTT

>AtGA20ox3

CCCAAATGCCGCTTACGTACTATTCCCTGCACCTAAGTTTCCCTCTCGCACCTATATATACCACTCCTTT
CCTCTCCCACTTACCGACCACTGTACTGAATCTTTAAGCCTCTCAACGTGTTTTTATATATATTTTGAA
AATCTTTTACGCCTTAAAAGGATCTACGATAATTAAAAAAAAATGGCAACGGAATGCATTGCAACGGTCC
CTCAAATATTCAGTGAAAACAAAACCAAAGAGGATTCTTCGATCTTCGATGCAAAGCTCCTTAATCAGCA
CTCGCACCACATACCTCAACAGTTCGTATGGCCCGACCACGAGAAACCTTCTACGGATGTTCAACCTCTC
CAAGTCCCACTCATAGACCTAGCCGGTTTCCTCTCCGGCGACTCGTGCTTGGCATCGGAGGCTACTAGAC
TCGTCTCAAAGGCTGCAACGAAACATGGCTTCTTCCTAATCACTAACCATGGTGTCGATGAGAGCCTCTT
GTCTCGTGCCTATCTGCATATGGACTCTTTCTTTAAGGCCCCGGCTTGTGAGAAGCAGAAGGCTCAGAGG
AAGTGGGGTGAGAGCTCCGGTTACGCTAGTAGTTTCGTCGGGAGATTCTCCTCAAAGCTCCCGTGGAAGG
AGACTCTGTCGTTTAAGTTCTCTCCCGAGGAGAAGATCCATTCCCAAACCGTTAAAGACTTTGTTTCTAA
GAAAATGGGCGATGGATACGAAGATTTCGGGAAGGTTTATCAAGAATACGCGGAGGCCATGAACACTCTC
TCACTAAAGATCATGGAGCTTCTTGGAATGAGTCTTGGGGTCGAGAGGAGATATTTTAAAGAGTTTTTCG
AAGACAGCGATTCAATATTCCGGTTGAATTACTACCCGCAGTGCAAGCAACCGGAGCTTGCACTAGGGAC
AGGACCCCACTGCGACCCAACATCTCTAACCATACTTCATCAAGACCAAGTTGGCGGTCTGCAAGTTTTC
GTGGACAACAAATGGCAATCCATTCCTCCTAACCCTCACGCTTTCGTGGTGAACATAGGCGACACCTTCA
TGGCTCTAACGAATGGAAGATACAAGAGTTGTTTGCATCGGGCGGTGGTGAACAGCGAGAGAGAAAGGAA
GACGTTTGCATTCTTCCTATGTCCGAAAGGGGAAAAAGTGGTGAAGCCACCAGAAGAACTAGTAAACGGA
GTGAAGTCTGGTGAAAGAAAGTATCCTGATTTTACGTGGTCTATGTTTCTTGAGTTCACACAGAAGCATT
ATAGGGCAGACATGAACACTCTTGACGAGTTCTCAATTTGGCTTAAGAACAGAAGAAGTTTCTAAAATTG

AGGAGACAATGTTGTGGTCCAGAAGGTCACTTGTAGTTATGTATAGAGCTTTCACTGTTTTTCTTAAGAT
GTTCAAGACTTGTTAAGGATCGGAGACTTTTTTGTTCTTTTATTATCTTGCTCTAGGTTGTCTGGTGTTA
AATAAAAGTAGCAAAAAAATAAAAATATATGTACTCCATTGGGTCTACTTATGATTTGTTTCGAAATGGT
TTTTTTATAGTCTCTAATGTTAAATTTATGTCACTATTACTATTATTAAATGAAGTTTATCAAGTAC

>*AtGA20ox4*
ATGGAATGCATCATAAAGCTCCCTCAAAGATTCAACAAAAACAAATCCAAAAAGAACCCTTTGAGGATCT
TCGATTCAACGGTTCTAAATCATCAACCAGACCATATACCTCAAGAGTTTGTATGGCCTGACCATGAGAA
ACCTTCCAAAAATGTTCCAATCCTCCAAGTCCCTGTCATCGACCTCGCAGGCTTCCTCTCCAACGACCCA
TTATTGGTCTCGGAGGCTGAAAGACTCGTCTCTGAAGCAGCAAAGAAACATGGTTTCTTCCTAGTCACCA
ACCACGGAGTTGACGAGAGGCTTTTGTCTACTGCCCATAAGCTTATGGACACATTCTTTAAGTCACCGAA
TTATGAGAAACTAAAGGCTCAGAGGAAGGTGGGTGAAACCACAGGCTACGCTAGTAGTTTTGTAGGGAGA
TTCAAGGAGAATCTTCCGTGGAAGGAAACGCTATCGTTTTCGTTTTCCCCGACGGAGAAGAGCGAAAACT
ACTCCCAAACCGTTAAAAATTACATTAGTAAGACAATGGGAGACGGATACAAAGATTTCGGGAGTGTTTA
TCAAGAATACGCGGAGACCATGAGCAATCTCTCTCTAAAGATCATGGAACTTCTTGGAATGAGTCTTGGT
ATCAAAAGGGAGCATTTTAGAGAATTTTTTGAAGACAATGAATCGATATTTAGATTAAATTACTATCCAA
AATGCAAGCAACCAGATCTTGTACTAGGTACGGGGCCTCACTGCGACCCAACCTCTCTAACTATACTTCA
ACAAGATCAAGTCAGTGGTCTTCAAGTTTTTGTGGACAACCAGTGGCAATCAATCCCTCCTATACCTCAA
GCATTGGTGGTGAACATTGGCGACACTTTAATGGCTCTAACGAATGGAATATACAAGAGTTGCTTGCATC
GGGCGGTGGTGAACGGCGAGACGACAAGGAAGACACTTGCATTCTTCCTATGTCCGAAAGTGGACAAAGT
GGTGAAGCCACCAAGTGAATTAGAAGGTGAGAGAGCGTATCCCGATTTTACATGGTCTATGTTTCTTGAG
TTCACAATGAAGCACTATAGAGCAGATATGAACACTCTTGAAGAGTTTACAAATTGGCTCAAGAACAAAG
GAAGTTTCTGA

>*AtGA20ox5*
ATGTGCATATATGCATCTAGACAGACTGTCTGCCCCTATTTAACCCCCTTCAAAGTTAAAAGACCCAAGT
CAAGAGAAATGAATTCCTCCGACGTCAACTTCTCCCTACTCCAGAGCCAACCAAATGTTCCGGCTGAATT
CTTCTGGCCGGAGAAAGACGTTGCCCCTTCAGAAGGCGACCTCGATCTCCCAATCATCGACTTGAGTGGG
TTCTTGAATGGCAATGAGGCCGAGACGCAACTTGCAGCTAAGGCAGTGAAAAAAGCGTGCATGGCTCACG
GTACTTTTCTAGTGGTCAATCATGGCTTCAAGTCGGGCTTGGCTGAAAAGGCGCTTGAGATATCGAGTTT
GTTCTTTGGGCTATCCAAGGATGAGAAGCTAAGGGCTTATAGGATCCCGGGGAATATCTCTGGCTATACC
GCAGGTCATAGCCAAAGATTTTCCTCCAATCTTCCGTGGAATGAGACTTTGACTTTGGCCTTCAAAAAGG
GGCCACCCCATGTTGTTGAAGACTTTCTAACCTCAAGGCTAGGCAACCATCGTCAAGAGATCGGTCAAGT
GTTTCAAGAATTTTGTGATGCAATGAATGGACTGGTTATGGATTTGATGGAGCTATTGGGTATAAGTATG
GGTTTAAAAGACAGGACATACTACCGGAGATTCTTCGAAGATGGAAGTGGCATCTTCAGATGCAACTATT
ATCCGCCGTGCAAGCAGCCAGAGAAAGCCCTTGGTGTTGGCCCCCATAATGACCCAACGGCTATAACCGT
TTTGCTTCAAGACGATGTTGTGGGCTTAGAGGTCTTTGCCGCAGGTAGTTGGCAGACCGTTCGCCCTCGT
CCTGGTGCTCTTGTCGTCAACGTTGGAGACACCTTCATGGCATTGTCAAATGGAAACTACAGGAGTTGTT
ACCACAGGGCAGTGGTAAACAAGGAGAAAGTGAGAAGGTCATTAGTATTCTTCTCTTGTCCTAGAGAAGA
TAAAATCATCGTACCTCCTCCTGAACTTGTGGAAGGTGAAGAAGCTTCTAGAAAGTACCCTGACTTTACT
TGGGCTCAGCTTCAGAAATTTACCCAGTCCGGCTATCGAGTCGATAACACCACTCTCCATAACTTCTCTT
CGTGGCTCGTCTCTAACTCCGACAAGAAATCAACTTGACTTCAGCTTTCTCGATTCATATTTCCTGGCGC
CCTTCGATTATCAACTAATAAAACATCTT

>AtGA2ox1

CTTTATCATCTCATCTGCTATATATACACTCCTCCGAGTACATAACCCAATGTCTCAGCCTTCAAAGCTA
TCAAAATCAAAAAAATTCTATCAAACAAGGAAATATATCAATGGCGGTATTGTCTAAACCGGTAGCAATA
CCAAAATCCGGGTTCTCTCTAATCCCGGTTATAGATATGTCTGACCCAGAATCCAAACATGCCCTCGTGA
AAGCATGCGAAGACTTCGGCTTCTTCAAGGTGATCAACCATGGCGTTTCCGCAGAGCTAGTCTCTGTTTT
AGAACACGAGACCGTCGATTTCTTCTCGTTGCCCAAGTCAGAGAAAACCCAAGTCGCAGGTTATCCCTTC
GGATACGGGAACAGTAAGATTGGTCGGAATGGTGACGTGGGTTGGGTTGAGTACTTGTTGATGAACGCTA
ATCATGATTCCGGTTCGGGTCCACTATTTCCAAGTCTTCTCAAAAGCCCGGGAACTTTCAGAAACGCATT
GGAAGAGTACACAACATCAGTGAGAAAAATGACATTCGATGTTTTGGAGAAGATCACAGATGGGCTAGGG
ATCAAACCGAGGAACACACTTAGCAAGCTTGTGTCTGACCAAAACACGGACTCGATATTGAGACTTAATC
ACTATCCACCATGTCCTCTTAGCAATAAGAAAACCAATGGTGGTAAGAATGTGATTGGTTTTGGTGAACA
CACAGATCCTCAAATCATCTCTGTCTTAAGATCTAACAACACTTCTGGTCTCCAAATTAATCTAAATGAT
GGCTCATGGATCTCTGTCCCTCCCGATCACACTTCCTTCTTCTTCAACGTTGGTGACTCTCTCCAGGTGA
TGACAAATGGGAGGTTCAAGAGCGTGAGGCATAGGGTTTTAGCTAACTGTAAAAAATCTAGGGTTTCTAT
GATTTACTTCGCTGGACCTTCATTGACTCAGAGAATCGCTCCGTTGACATGTTTGATAGACAATGAGGAC
GAGAGGTTGTACGAGGAGTTTACTTGGTCTGAATACAAAAACTCTACCTACAACTCTAGATTGTCTGATA
ATAGGCTTCAACAATTCGAAAGGAAGACTATAAAAAATCTCCTAAATTGATTGATATATCTATTTAATCT
ATAAGTGTGTGCTACATACAGACAATGCATCTGTATATTTTGAAGTTAATGTTATTTGTTAATCCAATAA
CTGTAAAAACATGCAAGAGTGTGTTTGTTTGTTTCGTAATATCAACATCGCTCCCATCTTTTATGGATAA
TATCAAGTTGTTTAAACACACTGTTTTGATGTAAGCTACATTTTACTTTAT

>AtGA2ox2

CCATGTGTGCGAGATACAAGAATAGACTCGACATGCAAAATAAAAATAAATATGACCAAATAATCATATA
ATATCTTAGAATAAATAAATTAAAAAATCATAGTAACCATTTTATAAATTTCGTAACCTTTTCTCCGTCA
TCCCCCACATACCACTCCCTATAAATGCCAAGCAAAATAGCTTTCCTCGATAGTTCCTAAATCATAACTA
CAAAAACTTTTCCTTCTTCTTCCTCAACCTTTGCTTCAATCTTCAACAACTTTCTTTTTATAAAGATTTT
GCAAGTTAAGTGTAAACCTACAAAAACCAAACATGGTGGTTTTGCCACAGCCAGTCACTTTAGATAACCA
CATCTCCCTAATCCCCACATACAAACCGGTTCCGGTTCTCACTTCCCATTCAATCCCCGTCGTCAACCTA
GCCGATCCGGAAGCGAAAACCCGAATCGTAAAAGCCTGCGAGGAGTTCGGGTTCTTCAAGGTCGTAAACC
ACGGAGTCCGACCCGAACTCATGACTCGGTTAGAGCAGGAGGCTATTGGCTTCTTCGGCTTGCCTCAGTC
TCTTAAAAACCGGGCCGGTCCACCTGAACCGTACGGTTATGGTAATAAACGGATTGGACCAAACGGTGAC
GTTGGTTGGATTGAGTATCTCCTCCTCAATGCTAATCCTCAGCTCTCCTCTCCTAAAACCTCCGCCGTTT
TCCGTCAAACCCCTCAAATTTTCCGTGAGTCGGTGGAGGAGTACATGAAGGAGATTAAGGAAGTGTCGTA
CAAGGTGTTGGAGATGGTTGCCGAAGAACTAGGGATAGAGCCAAGGGACACTCTGAGTAAAATGCTGAGA
GATGAGAAGAGTGACTCGTGCCTGAGACTAAACCATTATCCGGCGGCGGAGGAAGAGGCGGAGAAGATGG
TGAAGGTGGGGTTTGGGGAACACACAGACCCACAGATAATCTCAGTGCTAAGATCTAATAACACGGCGGG
TCTTCAAATCTGTGTGAAAGATGGAAGTTGGGTCGCTGTCCCTCCTGATCACTCTTCTTTCTTCATTAAT
GTTGGAGATGCTCTTCAGGTTATGACTAACGGGAGGTTCAAGAGTGTTAAACACAGGGTCTTAGCCGATA
CAAGGAGATCGAGGATTTCAATGATATATTTCGGCGGACCGCCATTGAGCCAGAAGATCGCACCATTGCC
ATGCCTTGTCCCTGAGCAAGATGATTGGCTTTACAAAGAATTCACTTGGTCTCAATACAAATCTTCTGCT
TACAAGTCTAAGCTTGGTGATTATAGACTTGGTCTCTTTGAGAAACAACCTCTTCTCAATCATAAAACCC
TTGTATGAGAGTAGTCATGATGATCTTTATCATCCTTTGTACGATAGAAAGTCATAATCACAAAAAGAAG
GAAATGGATAGTGTTTTGGATTAGCTTTTTTATGTTTGGTATGTTTTTCATTAGGTACAAAAGCTTTAGG
CTTTGCTTAGAGTTTGACCCAACCAAACCTAACTTTTGTATCTTCTCGTCTTCTTCTTCTTTTTGCCATA

ATTTGTTTATCTTTCTTGTAACAATTAGCTAGTGTTTATTTTCTCTTTATGTTTCATTTAACAGTTAATT
TTTTTACTTTTTTTTGCCATTTCCCTCATCAATTTCTTTTATCTATTATTCTATTACTATCACTTTGGTT
ATAAAATATAATATATGTAAATATATAGATATGCGTTGTACGTGTTAAAACTAGTGTTACACCAAATTTA
CGTTCTAATAGAAACAAAAGAAAAGTCTTCCACTTTATGTATGCCCTCATTGTGTCATCATG

>AtGA2ox3

AGAACAAGCGACTATAAATTAGTGCTAAGAAAACACCCTTTTCACCAAATTCACTCTCTTTTCAACAATC
TTTATTTTTCAAAGTTCTTGTTTAAAAGACCAAAAGAGTAGACCATGGTAATTGTGTTACAGCCAGCCAG
TTTTGATAGCAACCTCTATGTTAATCCAAAATGCAAACCGCGTCCGGTTTTAATCCCTGTTATAGACTTA
ACCGACTCAGATGCCAAAACCCAAATCGTCAAGGCATGTGAAGAGTTTGGGTTCTTCAAAGTCATCAACC
ATGGGGTCCGACCCGATCTTTTGACTCAGTTGGAGCAAGAAGCCATCAACTTCTTTGCTTTGCATCACTC
TCTCAAAGACAAAGCGGGTCCACCTGACCCGTTTGGTTACGGTACTAAAAGGATTGGACCCAATGGTGAC
CTTGGCTGGCTTGAGTACATTCTCCTTAATGCTAATCTTTGCCTTGAGTCTCACAAAACCACCGCCATTT
TCCGGCACACCCCTGCAATTTTCAGAGAGGCAGTGGAAGAGTACATTAAAGAGATGAAGAGAATGTCGAG
CAAATTTCTGGAAATGGTAGAGGAAGAGCTAAAGATAGAGCCAAAGGAGAAGCTGAGCCGTTTGGTGAAA
GTGAAAGAAAGTGATTCGTGCCTGAGAATGAACCATTACCCGGAGAAGGAAGAGACTCCGGTCAAGGAAG
AGATTGGGTTCGGTGAGCACACTGATCCACAGTTGATATCACTGCTCAGATCAAACGACACAGAGGGTTT
GCAAATCTGTGTCAAAGATGGAACATGGGTTGATGTTACACCTGATCACTCCTCTTTCTTCGTTCTTGTC
GGAGATACTCTTCAGGTGATGACAAACGGAAGATTCAAGAGTGTGAAACATAGAGTGGTGACAAATACAA
AGAGGTCAAGGATATCGATGATCTACTTCGCAGGTCCTCCTTTGAGCGAGAAGATTGCACCATTATCATG
CCTTGTGCCAAAGCAAGATGATTGCCTTTATAATGAGTTTACTTGGTCTCAATACAAGTTATCTGCTTAC
AAAACTAAGCTTGGTGACTATAGGCTTGGTCTCTTTGAGAAACGACCTCCATTTTCTCTATCCAATGTTT
GAGCTATTATTATTTTTTGCTTCTTGTAATCAGGCCCATATAGGACATCTTTTAGGGGGGGTTTCAAAAT
ATATGTTTATGGGTCGAATATTAATTTGGAAGCCTTCAAATTCTGTTATTAAGCCAAGTCCATTACCGAG
GACGAACAAGTCGTAATCAGGAAACTGAGATAAGAAG

>AtGA2ox4

GAAAATTTCTTCCTTATCATCTTCTCTCATAAAATCCCTCTCTCCCTTTCTCTAAAAATGGTGAAAGGGT
CCCAGAAAATCGTGGCCGTAGATCAAGACATACCAATAATAGACATGTCGCAGGAGAGATCACAAGTGTC
GATGCAGATAGTCAAAGCCTGCGAGAGTCTCGGCTTCTTCAAAGTCATCAACCATGGCGTTGACCAAACC
ACCATCTCAAGAATGGAGCAAGAGTCTATAAACTTCTTTGCTAAACCGGCTCACGAGAAGAAATCTGTCC
GACCAGTTAACCAGCCTTTCCGGTATGGTTTTAGAGACATTGGACTCAACGGTGACTCTGGTGAGGTCGA
GTATTTGCTGTTTCACACTAACGACCCTGCCTTTCGCTCTCAGCTCTCCTTCAGCTCGGCAGTGAATTGT
TACATAGAAGCAGTTAAGCAGTTGGCTCGTGAGATCTTAGATCTGACGGCTGAGGGACTTCATGTCCCAC
CTCACAGTTTCAGTAGGTTAATCAGCTCCGTCGATAGTGACTCCGTTCTGAGAGTGAATCATTATCCACC
GTCCGATCAATTCTTTGGTGAAGCCAATCTTTCTGACCAATCTGTGTCACTGACAAGAGTTGGCTTCGGA
GAACACACCGACCCTCAGATTTTAACAGTTCTTAGATCTAACGGTGTAGGAGGGCTCCAAGTGTCCAATT
CAGATGGCATGTGGGTTTCTGTCTCCCCTGACCCTTCAGCTTTCTGCGTCAATGTAGGAGACTTGTTACA
GGTGATGACGAACGGGAGATTTATAAGTGTAAGGCATAGAGCATTGACCTACGGAGAAGAAAGCCGGCTA
TCCACGGCGTACTTTGCCGGACCACCGCTTCAGGCGAAGATTGGGCCTCTTTCGGCGATGGTTATGACGA
TGAATCAGCCACGGTTGTACCAAACATTTACTTGGGGCGAGTACAAGAAACGTGCGTACTCTCTACGACT
TGAGGATAGCCGTTTAGACATGTTTCGTACATGTAAGGACTAGTATTCAGGTTTATGGCAAAGCTTTTTA
GTAATATCTATGAAATGTTTTCGTCTAACTATGTTCCCTTAAAAAAGTTTAAGCTAAACGTAGACATGTT
AGATCTGTGTACCTAGAACTAGGAGTTGAAAGTTTTGTAATGTGTTACCTTAACTTTGTTGAGGTGAAAT

GTGATACCCCACTATATTAAGGAAGTGATATTAGATAAAA

>AtGA2ox6

GAAAAAAGTCTTTGAATTTAAAATATTTATTTTATCCTTCCTTCTCTTCCCAATTCCCATACACTATATA
TATAAGAGCTCGTCACTCTTCTTTCAGATTCAAAACCTCCTGATTTCATTCTTTACTACTAATAATCATT
TTTTGAAAAAAATAAAAAAGGGACATGGTTTTGCCATCTTCAACACCGTTACAAACCACCGGAAAGAAGA
CGATATCCTCGCCGGAATATAACTTCCCGGTGATCGATTTCTCCTTAAACGATCGGTCAAAACTATCGGA
GAAGATCGTAAAAGCCTGTGAGGTTAATGGGTTTTTCAAGGTGATAAACCATGGAGTTAAACCGGAGATA
ATAAAGAGATTCGAGCATGAAGGAGAAGAGTTCTTTAATAAACCGGAATCAGATAAGCTACGAGCCGGTC
CGGCGAGTCCGTTCGGTTACGGATGCAAGAACATCGGGTTCAACGGCGATTTGGGTGAGCTTGAGTATCT
TCTTCTCCACGCAAATCCGACAGCTGTCGCTGATAAATCTGAAACTATATCTCATGATGATCCTTTCAAG
TTCAGCTCGGCGACGAATGATTACATACGCACGGTTAGAGATTTGGCATGTGAGATCATTGATCTGACGA
TCGAGAATTTATGGGGACAGAAGTCTAGCGAAGTGAGTGAGCTGATCAGAGACGTTCGTAGCGATTCGAT
CCTACGGTTGAATCACTATCCACCAGCACCGTACGCGTTAAGTGGCGTTGGTCAAATAGGTTTCGGAGAA
CATTCTGACCCTCAGATCTTGACGGTGTTAAGATCCAACGACGTAGATGGACTTGAGATTTGCTCACGTG
ACGGCTTGTGGATCCCAATCCCATCTGACCCTACATGCTTCTTCGTATTGGTCGGTGACTGCCTTCAGGC
ATTGACGAATGGGAGATTCACTAGCGTGAGGCATAGGGTTTTAGCAAACACAGCAAAGAAGCCACGTATG
TCGGCGATGTACTTTGCAGCTCCACCGTTAGAGGCGAAAATATCACCGTTGCCAAAAATGGTGTCGCCGG
AAAATCCAAGACGTTATAATTCATTCACATGGGGTGACTACAAAAAAGCTACATACTCTCTCCGTTTAGA
TGTCCCTCGTCTCGAGTTTTTCAAGACTTTATAGATTATATTCATACCATTGAAAGCTAGATTTTTTATT
TTTTTTTTGCTTGTTGAAAAAGAAAATATGATTGTTGGATTGTTACAAATATATACCATGTGAAATGTAT
AGATTTAGATGAACAAAAAGAAAAAGAAAATTGAGTTTTGGTCATTTACTTTGTAATTTACTAATTTCAC
ATGTGAATCAGTGGATCATTGCAATTTCTAAAGAAAAGCTCTAAAACATTCCAGTAAGAGAAATTATACC
TTTTTTTTCTTCAA

>AtGA2ox7

GCAAAATATAAGAATCGTATAGAAAACAATCACTTGTCAAATTTTAATTATTTCTTATCATGTACTATAG
CTTTACATGGCTTCTCAACCTCCCTTTAAGACAAATTTCTGCTCGATTTTCGGAAGCTCATTTCCAAATT
CAACTAGTGAGAGCAATACAAACACATCAACTATCCAAACCTCAGGCATAAAGCTTCCTGTGATCGATCT
CAGCCATCTAACTAGTGGTGAGGAGGTCAAACGCAAAAGATGTGTGAAACAAATGGTTGCAGCTGCGAAA
GAGTGGGGATTTTTTCAAATTGTGAACCATGGAATTCCCAAAGACGTCTTTGAGATGATGCTCCTCGAAG
AGAAGAAACTCTTTGACCAACCTTTTTCTGTGAAAGTCAGAGAACGTTTTTCGGACTTATCGAAGAATAG
TTACCGTTGGGGAAACCCTAGCGCCACTTCTCCCGCTCAGTACTCCGTTTCGGAAGCGTTTCACATCATT
CTTTCAGAGGTTTCAAGGATTTCTGATGATCGCAACAACCTCAGAACAATCGTTGAAACGTATGTGCAAG
AGATAGCTCGAGTGGCACAAATGATATGTGAAATACTGGGGAAACAAGTGAACGTGAGTTCGGAGTATTT
CGAAAACATTTTTGAGCTTGAAAACAGTTTTCTAAGGCTCAATAAGTACCATCCTAGTGTTTTTGGTTCT
GAAGTGTTTGGTTTGGTTCCTCATACCGATACAAGTTTTCTCACTATACTCTCTCAAGATCAAATCGGAG
GGTTAGAATTGGAAAATAATGGACAATGGATCAGCGTAAAACCTTGCTTGGAAGCCCTTACAGTCAACAT
TGGGGATATGTTTCAGGCACTGAGTAATGGAGTGTACCAAAGCGTGAGACATAGAGTGATTTCTCCAGCA
AATATCGAGAGGATGTCAATAGCTTTCTTCGTATGTCCTTATCTCGAAACTGAGATCGATTGCTTTGGGT
ATCCAAAGAAGTATAGAAGATTCAGTTTCAGAGAGTACAAAGAGCAGAGTGAACATGATGTTAAAGAAAC
TGGTGATAAGGTAGGCTTGTCCAGGTTTCTCATTTGATCCATCTCTTTGAACTTTGACCTTTAGTGGAGA
GGTCAAAACTAATGTTGTCTTATTTTGGCAATTCCTCAAACTCAATTTAACACCTATGTTTAAGGCAAGG
TCTAATAAAAATCGAT

>AtGA2ox8

TGATAACCTTCACTTCCCATAATTCTCTCTAACTCATCACTCTTTTTCTTCTTCTGCATAGATCTTCTCA
TGGATCCACCATTCAACGAAATATACAATAACCTTTTGTATAATCAGATCACAAAGAAAGATAACGATGT
TTCTGAAATACCCTTTAGCTTCTCAGTCACAGCCGTCGTCGAGGAGGTGGAGCTTCCTGTGATTGACGTC
AGCCGTTTGATTGATGGAGCCGAGGAGGAGAGAGAGAAATGTAAGGAAGCGATTGCGAGAGCTTCGAGGG
AGTGGGGATTTTTCAAGTGATAAACCATGGAATATCAATGGATGTGTTGGAGAAGATGAGACAAGAGCA
AATTAGGGTCTTTAGAGAGCCTTTTGACAAGAAAAGTAAGTCGGAGAAATTTTCCGCCGGGAGTTACCGG
TGGGGAACGCCGTCAGCCACTTCTATCCGGCAGCTTTCTTGGTCAGAAGCTTTTCATGTTCCCATGACAG
ATATTTCTGACAACAAGGACTTTACTACTCTCAGCTCAACAATGGAGAAATTTGCTTCGGAATCAGAGGC
ATTAGCATATATGTTGGCAGAGGTTCTTGCAGAAAAATCTGGACAAAATTCAAGTTTCTTCAAAGAAAAC
TGTGTGAGAAATACATGTTATCTAAGGATGAACCGATATCCACCTTGTCCCAAACCATCGGAGGTGTACG
GATTAATGCCACACACGGACAGTGATTTCCTCACAATCTTGTATCAAGATCAAGTCGGAGGACTCCAACT
TATCAAAGACAATAGATGGATCGCTGTTAAACCTAATCCTAAAGCTCTCATTATCAATATTGGTGACTTA
TTTCAGGCATGGAGCAATGGCATGTACAAAAGTGTTGAACACCGTGTGATGACGAACCCAAAGGTGGAGA
GATTCTCAACGGCTTATTTATGTGTCCATCATACGACGCCGTTATAGAGTGTTCAAGTGATCGTCCTGC
TTATAGAAATTTCAGCTTCAGAGAATTCAGACAACAAGTTCAAGAAGATGTTAAGAAGTTTGGTTTTAAA
GTTGGCCTTCCTAGGTTCCTTAATCACGTCTACTAATTTATACACCCAAATTTTTATTTTTCCTTTATAA
TTTATTTTAGGTTTTATGATTTATTTGAATTGTTTTTGGTTTGATTTTTTCCATATAATTTATTTTTCAT
GTTTTTCCTTAAGAAATATATTGTTCCTCATTATCAAATTTAATTAAAATCGATTAGAAAACCAA

>AtGA2ox9

ATCCTTTGTAACTAAATGCACCTATAGATACACATCAAACACTTCATTTGTTTTAAGTAATTTAATAGACTTTTTAAAGGATAAAAT
CGAAAGAGATCTCAAGAGAAATAGGGAGAGATGAGCATCAGTGAGTCATATCCACCGGCTTTCCGGCGTGTAACCAACGACGA
AAATCCTCCAGCAACACCGGTTATTGTCCAGGGCAAGGATATCAATATCCCGGTTATCGATTTGGAACGTTTGGACAAGGAGATA
CTGAGAGAGGCATGCAAGGAGTGGGGAATATTCCGTCTAGAGAATCACGGAGTACCGTTGGCGTTAACGTCACGGCTTCAAGA
AATCTCGGAGTCATTACTGAGTCTTCCGTTTGAGAAAAAACGGGAATTGTTCGCTGCCGTTAAATCTCCGTTATCGTATTTTTGG
GGAACACCAGCTCTAAATCGTTCAGGAGATGCACTAAAGAGAGGAGCTCAAGCTTCGAACTTAACCATGCTCGAAGGTTTCAA
CGTTCCTCTCTCGTCTCTCTCGTCGCTCTCGAAGCTTCCAACTTCTACTTGTTGTGATGATGATGCTCAAGAAGAGCCTAAACTT
GAGTCTTTCAGAGTGTTGATGGAGGAATATGGAAAGCACATAACTAGAATTGCTGTGTCATTGTTCGAAGCTATAGCACAAACA
CTGAACCTAGAACTATCCGGTAACCGGAGATCAGAATACTTATCGGAATCGACAGGGCTAATAAGGGTTTACCGGTATCCACAA
AGCTCCGAGGAAGCAGCCAGAGAAGCTCTAGGAATGGAAGTTCACACAGACAGTTCGGTTATCTCAATATTAAGAGAAGATGA
AAGTGGTGGGCTTGAGATCATGAAAGGTGAAGAATGGTTCTGTGTAAAGCCTGTTGCTAATACTCTTATCGTCAATCTTGGAGAT
ATGATGCAGGCCATAAGCGATGATGAGTACAAGAGTGTGACACATAGAGTGAAGAAGAGGAATAGGAAGACAGAGAGACACT
CAGTGTGTTATTTTGTGTTTCCAAAGAGAGATTGCGTGATAAAGTCATCAAACTATAAGCTATTCACTTACTCAGATTTTGAAGCT
CAAGTTCAAGCTGATGTTCAGTCTCTTGGAACCAAGATTGGCCTTCCTAGATTCAACCCAAATTCTCCATTGTTTCTCTGACCGT
GTCTGAAATTTAGTATCTATTTGGTTTACCAAACTCTGGATACTTAAGTTAGTCCATCAATAAGACTCAAACAAAGAAAGCTGTG
TTTTTGACTCTGAAATAATTTTGGGCTGGCCCTGATCATCGTAGAGAACTTGACAATAACATTGTTTGTTGAACATTAAAAACAC
TCTTTGTTTGCTCTACAATGACGACGATCCATCTACTTATTTTCTATTGTGATAGTGATATTTCAACAAACAGTATTTGAAAATTGG
TTTGTGGCATTGTAAAGCAATTCTGCTTTAGTGATAATTGCCATTATGAAGCAGTTTTG

>AtGA2ox10

CGGGTGCACTTCAAAGGAGAACATGAGCAACTCGGAGTCTTATCCACCAGAGTTTCGACCTCTAATGAGCGAAAAATCCACTG

AAACGGGTCTAGACCGGAGCAAAGATATCGATATCCCGGTTATCGATATGGAGCATTTGGACATGGAGAAACTGAGAGAAGCAT
GCAAAGACTGGGGGATCTTCCATTTGGAGAATACTGGGATACCCTTAACGTTTATGTCACAGGTGAAAGAGATAACGGAGTCAG
TGTTGAGTCTGCCGTTTGAAGAGAAACGGACGTTGTTCGGCGTTAACTCTCCGTTATCGTATTACTGGGGAACACATACGGTAA
GTCCATCGGGAAAAGCCGTAACGAGAGCCCCACAAGAATCTAGTGGTCACTTGTTCGAAGGCATCAATATTCCTCTTGCATCTC
TCTCTCGGCTTCTTGCACTTTCTTGTACTGATCCCAAGCTTGAGTCTTTCCGAGTGGTGATGGAGGAATATGGGAAGCACGTAAC
TAGGATTATTGTGACCCTATTTGAAGCTATAATAGAAACCTTGTCCCTTGAATTATCCGGTGACCAAAAAATGGGCTACTTATCAG
AGTCAACAGGTGTGATACGTGTTCAACGGTATCCTCAGTGTACCGAATCCCCCGGACTCGAAGCTCATACGGACAGTTCGGTGA
TATCGATCATAAACCAAGACGATGTTGGTGGGCTAGAGTTCATGAAAGATGGGGAATGGTTCAATGTCAAACCTCTTGCCAGTT
CTTTTGTCGTCGGTCTTGGGGATATGATGCAGGTGATAAGTGATGAGGAATACAAGAGTGTGTTGCATAAAGTAGGAAAGAGGA
TGAGGAAGAAAGAGAGATACTCGATAGTTAACTTTGTGTTCCCGGACAAAGACTGTATGTTTAACTCTACTCGCTATAAGCCATT
CAAATTCTCGGAGTTTGAAGCTCAAGTTAAGCTTGATGTTGAGACCCATGGTAGCAAAGTCGGCCTTTCTAGGTTCCTGAGCAA
CCCCTAGTCTTGTATTCTCTTATTTTCTCCGAATATGTATATTTACAATTTGCTTTTTCTTTGTATCCTAAAAAATACTTATATTTGCA
TGGCAAAACTTTTGTATTTGCAAGGTTTATTTGTGTATTAAACTTTTATATA

>AtGA3ox1

GGCGGGACCTCTATAATTTTCTAGAGGTTTGGTTTGTTCGTATTGTATTAATCCAATAAACAAGTAGAAT
AACCAATAATATTGGACCAAAACAAGTGCTTTAACATATGCTTTTGCCTCCTCTTGGTCTCTACTCCACC
ACTATAAATAAAACTCTCTAACTCTTCCAATCTCCCATCACCAAACACCACACTTCTCATAAGAAAAAAA
ACACAAACATCTATCAAATTTACAAAGTTTTAAAACTAATTAAAAAAGAGCAAGATGCCTGCTATGTTAA
CAGATGTGTTTAGAGGCCATCCCATTCACCTCCCACACTCTCACATACCTGACTTCACATCTCTCCGGGA
GCTCCCGGATTCTTACAAGTGGACCCCTAAAGACGATCTCCTCTTCTCCGCTGCTCCTTCTCCTCCGGCC
ACCGGTGAAAACATCCCTCTCATCGACCTCGACCACCCGGACGCGACTAACCAAATCGGTCATGCATGTA
GAACTTGGGGTGCCTTCCAAATCTCAAACCACGGCGTGCCTTTGGGACTTCTCCAAGACATTGAGTTTCT
CACCGGTAGTCTCTTCGGGCTACCTGTCCAACGCAAGCTTAAGTCTGCTCGGTCGGAGACAGGTGTGTCC
GGCTACGGCGTCGCTCGTATCGCATCTTTCTTCAATAAGCAAATGTGGTCCGAAGGTTTCACCATCACTG
GCTCGCCTCTCAACGATTTCCGTAAACTTTGGCCCCAACATCACCTCAACTACTGCGATATCGTTGAAGA
GTACGAGGAACATATGAAAAAGTTGGCATCGAAATTGATGTGGTTAGCACTAAATTCACTTGGGGTCAGC
GAAGAAGACATTGAATGGGCCAGTCTCAGTTCAGATTTAAACTGGGCCCAAGCTGCTCTCCAGCTAAATC
ACTACCCGGTTTGTCCTGAACCGGACCGAGCCATGGGTCTAGCAGCTCATACCGACTCCACCCTCCTAAC
CATTCTGTACCAGAACAATACCGCCGGTCTACAAGTATTTCGCGATGATCTTGGTTGGGTCACCGTGCCA
CCGTTTCCTGGCTCGCTCGTGGTTAACGTTGGTGACCTCTTCCACATCCTATCCAATGGATTGTTTAAAA
GCGTGTTGCACCGCGCTCGGGTTAACCAAACCAGAGCCCGGTTATCTGTAGCATTCCTTTGGGGTCCGCA
ATCTGATATCAAGATATCACCTGTACCGAAGCTGGTTAGTCCCGTTGAATCGCCTCTATACCAATCGGTG
ACATGGAAAGAGTATCTTCGAACAAAAGCAACTCACTTCAACAAAGCTCTTTCAATGATTAGAAATCACA
GAGAAGAATGATTAGATAATAATAGTTGTGATCTACTAGTTAGTTTGATTAATAAATTGTTGTAAATGAT
TTCAGCAATATGATTTGTTTGTCCTCAATCATATCTGAACTGCATTACATATCAAAATTGCAATTTCCCA
AAAAAATCTAGCACTTTCTGGAATCGCCCCATGACATGTTGCCACTAATGAG

>AtGA3ox2

TTGATAACCAATAAGAATCGACAAAAAAAAAAACTTACTCCACATATACTCTTCTCTTCACTCTTCAGTCT
TCACTATTCAGTCTCGAGTATTTCACCGATCTATAAATACACTCCTCTTCTCCACCAAAAGTATCATATC
ATACCAAAAACATAAAGCCAAAATATAAACACATAAGCCTTTTAGCATGAGTTCAACGTTGAGCGATGTG
TTTAGATCGCATCCCATTCACATCCCACTCTCAAACCCACCTGACTTCAAATCTCTCCCGGATTCTTACA
CGTGGACTCCTAAAGATGATCTCCTCTTCTCCGCCTCCGCCTCCGACGAAACCCTGCCGCTCATCGACCT
CTCCGATATCCACGTGGCCACTCTTGTGGGCCATGCTTGTACCACGTGGGGAGCGTTCCAGATCACCAAC
CACGGCGTCCCCTCGCGACTTCTCGACGACATTGAGTTCCTCACCGGAAGTCTTTTCCGGCTTCCCGTAC

AGCGGAAGCTCAAGGCGGCTCGGTCAGAGAATGGCGTCTCCGGCTACGGCGTAGCTCGTATTGCTTCGTT
CTTTAATAAGAAGATGTGGTCCGAAGGTTTCACCGTTATTGGCTCTCCCCTCCACGATTTCCGTAAACTC
TGGCCCAGCCACCACCTCAAATACTGTGAAATTATTGAAGAGTATGAAGAACATATGCAAAAGTTGGCAG
CCAAGTTGATGTGGTTCGCATTAGGTTCACTGGGAGTTGAAGAAAAGGACATACAATGGGCCGGGCCTAA
TTCAGACTTTCAAGGAACCCAAGCAGTTATCCAACTAAACCATTATCCAAAATGTCCAGAACCAGACAGA
GCCATGGGCCTCGCAGCCCATACAGACTCGACCCTCATGACAATTCTGTACCAGAACAACACCGCCGGTC
TCCAAGTTTTCCGGGATGACGTGGGCTGGGTTACCGCGCCACCTGTCCCTGGCTCGCTGGTGGTCAACGT
CGGTGACTTGCTCCACATTTTAACCAACGGAATCTTCCCGAGCGTGCTTCACCGAGCCAGGGTTAACCAC
GTCCGATCTCGGTTCTCAATGGCTTACCTGTGGGGTCCACCATCCGATATAATGATCTCTCCACTTCCCA
AACTGGTTGATCCTCTCCAATCTCCTCTCTACCCATCTCTCACTTGGAAACAATACCTTGCTACCAAAGC
TACTCATTTTAATCAATCTCTTTCCATTATTAGAAATTAACTGTCTTCCGACTGAATTTCTTGATTTTCA
GATTTTACTATTTATTTTCTTAGTAATATGATGATATCTATTACTGTTTTGATTTTAGATGAGTGGTTCT
TCAAATTCACAATTAGTAGCTTAATATTGATTTTGTCATGTTACATAAAAAGGGTCGTATAGTTTTTGTT
TTCCACAAATTTACAAAACTATGAAAACTTGCTAATGTTTTACATGAACTATATATAAG

>*AtGA3ox3*

ATGAGCTCTGTCACACAGCTATTCAAGAACAACCCCGTGAACCGTGACCGGATCATCCCACTAGATTTCA
CCAACACCAAAACCCTACCCGACTCCCACGTCTGGTCCAAACCCGAACCCGAAACCACGAGCGGACCCAT
TCCCGTCATCAGCTTGTCCAACCCGGAGGAACATGGGCTACTACGACAAGCCTGTGAGGAATGGGGTGTG
TTTCATATCACAGACCATGGAGTCTCACACTCGTTACTCCATAACGTTGATTGCCAAATGAAGAGGCTTT
TCTCTTTACCCATGCATCGTAAGATCTTAGCCGTTCGATCTCCCGACGAGTCCACTGGTTACGGTGTGGT
TCGGATCTCCATGTTCTATGATAAGCTCATGTGGTCTGAAGGATTCTCCGTCATGGGTTCCTCTCTTCGA
CGTCACGCTACACTCTTATGGCCCGATGATCATGCCGAGTTCTGCAATGTGATGGAAGAGTATCAGAAGG
CAATGGATGATTTAAGTCATAGACTAATAAGCATGTTGATGGGCTCGTTAGGGCTAACACATGAAGATTT
GGGATGGCTTGTACCAGACAAAACTGGTTCAGGAACTGACTCGATCCAATCCTTTCTGCAGTTGAACTCC
TACCCGGTTTGCCCTGACCCTCATCTAGCTATGGGTTTAGCCCCTCACACCGACTCCTCCCTACTTACCA
TTCTCTACCAAGGCAATATTCCAGGTCTAGAGATTGAAAGTCCACAAGAAGAAGGGTCGAGATGGATTGG
AGTAGAGCCAATAGAAGGTAGTCTCGTTGTTATAATGGGAGATTTATCTCATATCATATCCAATGGACAG
TTCAGAAGTACAATGCACCGTGCGGTGGTGAACAAGACGCACCACCGTGTCTCAGCCGCTTATTTCGCTG
GCCCTCCCAAGAACCTTCAGATCGGACCGTTGACCAGCGACAAGAACCATCCTCCTATTTACAGGCGTTT
GATATGGGAAGAGTACCTTGCAGCCAAAGCAACACACTTTAACAAAGCCTTGACTTTGTTCCGTTGCTGA

>*AtGA3ox4*

ATGCCTTCACTAGCAGAAGAGATATGTATTGGTAACTTAGGCAGTCTCCAAACACTCCCCGAGTCGTTCA
CCTGGAAACTCACAGCCGCCGACTCCCTTCTGCGTCCCTCCTCCGCCGTCTCATTCGACGCAGTGGAAGA
GTCCATTCCTGTGATCGACCTCTCTAATCCTGACGTTACCACCCTCATTGGAGATGCCTCCAAAACATGG
GGAGCGTTTCAGATAGCCAACCACGGGATTTCTCAGAAGCTTCTCGATGATATCGAGTCTCTGTCCAAAA
CCCTATTCGACATGCCGTCAGAGAGGAAGCTTGAAGCGGCTTCCTCCGATAAAGGAGTTAGTGGCTACGG
AGAACCTCGAATCTCCCCCTTTTTCGAGAAGAAAATGTGGTCTGAAGGGTTTACTATTGCCGATGACTCC
TACCGCAACCATTTCAATACTCTTTGGCCTCATGATCACACCAAGTACTGCGGTATAATCCAAGAATACG
TGGACGAAATGGAAAAATTAGCAAGCAGACTTCTGTATTGCATATTAGGCTCACTTGGTGTCACCGTGGA
AGACATTGAATGGGCTCACAAGCTAGAGAAATCTGGATCAAAAGTGGGCAGAGGCGCCATACGACTAAAC
CACTACCCGGTTTGTCCTGAACCAGAACGAGCCATGGGTCTAGCCGCTCATACAGACTCCACTATCCTAA
CCATTCTGCACCAGAGCAACACGGGAGGGCTACAAGTGTTCAGGGAAGAGTCCGGTTGGGTCACGGTTGA
GCCGGCTCCTGGTGTCCTCGTGGTCAACATTGGTGATCTCTTTCACATCTTATCGAACGGGAAAATCCCA
AGCGTGGTTCATCGAGCCAAAGTTAACCATACTCGGTCAAGAATTTCGATTGCGTACTTATGGGGTGGTC
CAGCTGGTGATGTGCAAATCGCACCTATCTCTAAGTTAACCGGTCCGGCTGAACCGTCTCTTTACCGGTC
AATTACATGGAAAGAGTATCTCCAAATAAAGTATGAGGTTTTCGACAAGGCCATGGACGCAATTAGGGTC

GTTAATCCCACCAATTAA

3、 rice sequences

>*OsGA20ox1-like* LOC4334841

TAATTTTAATTGATGCCGGGATAGAGAGAGAGAGAGAGTTAAGAAGGTAGCTAGGGGAGAGAGCGAGGTT
GATGCCGTGATCGATCGATCGATCTGTTGGCGCAGCGTGTATATAAGGGCGGGAAGGGGAGTGAGAGAGA
GCAGCAGCTAGCTAGCCGCGGTCGGTCGATCCAGCTGCTGGGGATGAGTACTTAGTTAGCTCGGAGCTAG
CTACTAATGGATGATATACTTATGCTAGTTAGTTAAATACAGTTATTAGTTAGTTGTAGGTTGCATCTAT
CATATCTCCATCGGTTAATTAATTGATTGATAGCTAGATTATCAACAATTAATGAGCATGGTGGTGCAGC
AGGAGCAGGAGGTGGTGTTCGACGCGGCGGTGCTGAGCGGGCAGACGGAGATCCCGTCGCAGTTCATATG
GCCGGCGGAGGAGAGCCCCGGGTCGGTGGCGGTGGAGGAGCTGGAGGTGGCGCTGATCGACGTGGGGGCG
GGGGCGGAGAGGTCGTCGGTGGTCCGGCAGGTGGGGGAGGCGTGCGAGAGGCACGGCTTCTTCCTGGTGG
TTAACCACGGCATCGAGGCGGCGCTGCTGGAGGAGGCGCACCGGTGCATGGACGCCTTCTTCACGCTGCC
GCTGGGGGAGAAGCAGCGGGCGCAGCGGCGCGGGGGAGAGCTGCGGCTACGCCAGCAGCTTCACGGGG
CGCTTCGCGTCCAAGCTGCCGTGGAAGGAGACGCTGTCGTTCCGGTACTCATCGGCTGGAGATGAAGAGG
GCGAGGAGGGCGTGGGTGAGTACCTGGTGCGGAAGCTCGGGGCGGAGCACGGGCGGCGGCTGGGCGAGGT
GTACTCGCGCTACTGCCACGAGATGAGCCGCCTGTCGCTGGAGCTGATGGAGGTGCTCGGGGAGAGCCTG
GGCATCGTCGGAGACCGGCGCCACTACTTCCGGCGATTCTTCCAGCGCAACGACTCCATCATGCGCCTCA
ACTACTACCCGGCGTGCCAGAGGCCACTCGACACGCTGGGCACCGGTCCGCACTGCGACCCCACCTCGCT
CACCATCCTCCACCAGGACCACGTCGGCGGCCTGGAGGTGTGGGCGGAGGGGCGGTGGCGCGCCATCCGC
CCTCGCCCCGGGGCGCTCGTCGTCAACGTCGGCGACACCTTCATGGCGCTCTCCAACGCCAGGTACCGCA
GCTGCCTGCACCGGGCGGTCGTCAACAGCACGGCGCCTCGCCGCTCGCTGGCCTTCTTCCTCTGCCCGGA
GATGGACACGGTGGTGCGCCCGCCGGAGGAGCTGGTCGACGACCACCACCCGAGGGTGTACCCGGACTTC
ACGTGGCGGGCGCTGCTGGACTTCACGCAGCGCCACTACAGGGCCGACATGCGCACGCTTCAGGCCTTCT
CCGACTGGCTTAATCATCATCGTCACCTGCAACCAACAATATACTCCTAGCTCCTAGTCCTAGCTATATA
CTCCTATTATCCATCCATCCATCCATCTTACACTACTATACCATTAGCATCGATCGATCATCCATTAATT
AATTAATTAATTACTAGTTCCGGCTTAGATATATATCTGGCGATTATTTCAGTTCCTAGCTACTCCTACA
TGCATGCTTTGCTTAATTAGATCTATCTATCTAATCTATCCCGGCCGGCCTGTTTTAATTCCATATATCA
TTTGGTTTGCACGTACCCATCTATGATCTATATATACATGCATGTCGACTATTGTTGGTCGTACGATATT
ATATTATATATAGATGTAATACATATGTTGAAAATATATTGATTTCTTCTTGCTGTAGGAGTAATTAATT
ACTCCAGTTGTTGCTGTCACTCGATCAATCCATGGGATATGGGGATCGATCGGACGATATGCAGGAAGCT
CAAGGACGATATGAATGATGCATGCA

>Os*GA20ox2-like1* LOC4325003

ACCCTCCTGCCCAGACAGCTCGCCCTGCACACACACACACACTCACACTCACACACGCTCTCAACTCACT
CCCGCTCAACACAGCGCTCACTTCTCATCTCCAATCTCATGGTGGCCGAGCACCCCACGCCACCACAGCC
GCACCAACCACCGCCCATGGACTCCACCGCCGGCTCTGGCATTGCCGCCCCGGCGGCGGCGGCGGTGTGC
GACCTGAGGATGGAGCCCAAGATCCCGGAGCCATTCGTGTGGCCGAACGGCGACGCGAGGCCGGCGTCGG
CGGCGGAGCTGGACATGCCCGTGGTCGACGTGGGCGTGCTCCGCGACGGCGACGCCGAGGGGCTGCGCCG
CGCCGCGGCGCAGGTGGCCGCCGCGTGCGCCACGCACGGGTTCTTCCAGGTGTCCGAGCACGGCGTCGAC
GCCGCTCTGGCGCGCGCCGCGCTCGACGGCGCCAGCGACTTCTTCCGCCTCCCGCTCGCCGAGAAGCGCC
GCGCGCGCCGCGTCCCGGGCACCGTGTCCGGCTACACCAGCGCCCACGCCGACCGCTTCGCCTCCAAGCT

CCCATGGAAGGAGACCCTCTCCTTCGGCTTCCACGACCGCGCCGCCGCCCCCGTCGTCGCCGACTACTTC
TCCAGCACCCTCGGCCCCGACTTCGCGCCAATGGGGAGGGTGTACCAGAAGTACTGCGAGGAGATGAAGG
AGCTGTCGCTGACGATCATGGAACTCCTGGAGCTGAGCCTGGGCGTGGAGCGAGGCTACTACAGGGAGTT
CTTCGCGGACAGCAGCTCAATCATGCGGTGCAACTACTACCCGCCATGCCCGGAGCCGGAGCGGACGCTC
GGCACGGGCCCGCACTGCGACCCCACCGCCCTCACCATCCTCCTCCAGGACGACGTCGGCGGCCTCGAGG
TCCTCGTCGACGGCGAATGGCGCCCCGTCAGCCCCGTCCCCGGCGCCATGGTCATCAACATCGGCGACAC
CTTCATGGCGCTGTCGAACGGGAGGTATAAGAGCTGCCTGCACAGGGCGGTGGTGAACCAGCGGCGGGAG
CGGCGGTCGCTGGCGTTCTTCCTGTGCCCGCGGGAGGACAGGGTGGTGCGGCCGCCGCCGAGCGCCGCCA
CGCCGCAGCACTACCCGGACTTCACCTGGGCCGACCTCATGCGCTTCACGCAGCGCCACTACCGCGCCGA
CACCCGCACGCTCGACGCCTTCACGCGCTGGCTCGCGCCGCCGGCCGCCGACGCCGCCGCGACGGCGCAG
GTCGAGGCGGCCAGCTGATCGCCGAACGGAACGAAACGGAACGAACAGAAGCCGATTTTTGGCGGGGCCC
ACGCCCACGTGAGGCCCCACGTGGACAGTGGGCCCGGGCGGAGGTGGCACCCACGTGGACCGCGGGCCCC
GCGCCGCCTTCCAATTTTGGACCCTACCGCTGTACATATTCATATATTGCAAGAAGAAGCAAAACGTACG
TGTGGGTTGGGTTGGGCTTCTCTCTATTACTAAAAAAAATATAATGGAACGACGGATGAATGGATGCTTA
TTTATTTATCTAAATTGAATTCGAATTCGGCTCA

>Os*GA20ox2-like2*   LOC9269090

TATATTGTCGTTGTTGTCCTTCTTGACAGTGAAAACCAGGCATATATACAAGCTTGGCGTGTGCGTCACT
CGCACCCAGCTTGCTCGCTCGCTTGATCGACACATCACACATGCATGCATCTCCTCACCCATTACAAATA
GCACACGACACTCTGCTCCTTTCTCTCACACACACACTCTGCACTGCAGAAACCGGCTCGACCATACGTA
CAGGTACAGCCATGGTGTACATCTCGAACGCGCAGGATGCCAGTAAGCTGATCGTGACGGCGAAGGGAGG
AGGAGGAGAAGCCGACGACGCCGCCGCCTCCTCCGCTGCCGTCGTCCTTGACCTCTGGCGGCAGCCGGCC
AAGATCCCGGCGCCGTTCGTCTGGCCGCGCGCCGACGTCGCGCTGCCGCCGTCGTCGCCGCCGACGGGGG
AGCTGGACGTGCCCGTGGTCGACCTCGCCGCGGCGCTACGCGACGCCGCCGGGATGCGCCGCGCCGTCGC
GCAGGTGGCCGCCGCGTGCGCGAGCCACGGGTTCTTCCAGGTGAGCGGGCACGGCGTGCCCCCGTCGCTG
GCGCGCGCCGCGCTCGACGGCGCCGCGGGCTTCTTCCGCCTCCCGCCCGCCGCGAAGCAGCGCGCCCGCC
GCGCCCCAGGCACGGTCACCGGCTACACCGCCGCCCACGCCGACCGCTTCGTCGACAACCTCCCCTGGAA
GGAGACGCTCTCCTTCGGCCACCGCCACGCCAACGCCGCCGGCAACAACTCCTCCACCGTCGCCGATTAC
TTCTCCACCCTAGGCGACGACTTCAAACACCTAGGGGAGGTGTACCAGGAGTACTGCGAGGCGATGGAGG
AGGTGACGAAGGCGATAATGGCGGTGCTAGGGGAGAGCCTGGGGGTGGGGGGCGGATACTACAGGGAGTT
CTTCGAGGACAGCAGCTCGATCATGCGGTGCAACTACTACCCGCCGTGCCCGGAGCCGGAGAGGACGCTG
GGGACGGGGCCGCACTGCGACCCGTCGGCGCTCACCGTGCTCCTCCAGGACGGCGACGTCGACGGCCTCC
AGGTGCTCGTCGCCGGCGCGTGGCGCCCCGTGCGGCCGCTGCCCGGCGCGTTCGTCGTCAACATCGGCGA
CACCTTCATGGCGCTGACGAACGGCCGGTACAAGAGCTGCCTGCACCGGGCGGTGGTGCACCGGGAGCAG
GAGCGGCGGTCGCTGGCCTTCTTCCTCTGCCCGCGCGAGGACCGCGTGGTGCGGCCGCCCGCCGGCGCCG
GCGCCGGCGAGCGGCGGCTCTACCCGGACTTCACCTGGGCCGACTTCATGCGCTTCACGCAGCGCCACTA
CCGCGCCGACACCCGCACGCTCGACGCCTTCGCCCGCTGGCTCCGCCCGCCGGCCTGCTCCGGCGCCGCG
CCGGTCGTCGGCCCACCGACGACGGCGACCCAGGCGGCCACCGTCTAGGGCGTCCAGCTAGCTAGCTCAC
TGTCCATGTATACTAGTACGTATATAGTATACGTACATTTATGTTGGCCTAATTAATCGAACTAGTATGC
ATGTGTATATGACTATTGCACCTGTAATTTTTATTGTGGGAAGGCAGGAAGCCACGCGTGTGTGCTTTTA
TA

>*OsGA2ox1*   LOC4337874

GCGCCACCCTCCTCTCCTCCATCATCGCAGCCAACTCCAACATCCTGAGCTCCAATCTCCCACTCCATTC

CGGCCGCGCATTCTCCCGCTCTCGATCGATCGATCGATCATGGTGGTGCCTTCCGCGACGACGCCAGCGA
GGCAGGAGACGGTGGTGGCGGCGGCGCCGCCAGCTGCGGCGGCGTCCGGTGTCGTCGGCGGCGGCGGCGG
CGTGACGATAGCGACGGTGGACATGTCGGCGGAGCGCGGCGCGGTGGCGAGGCAGGTGGCGACGGCGTGC
GCGGCGCACGGGTTCTTCCGGTGCGTCGGGCACGGCGTGCCGGCGGCGGCGCCCGTCGCGGCGAGGCTGG
ACGCCGCGACGGCGGCGTTCTTCGCGATGGCGCCGGCGGAGAAGCAGCGCGCCGGGCCGGCGAGCCCGCT
CGGGTACGGCTGCCGGAGCATCGGGTTCAACGGCGACGTCGGCGAGCTGGAGTACCTGCTCCTCCACGCC
AACCCCGCCGCCGTCGCGCACCGGGCCAGGACCATCGACGCCATGGACCCCTCTCGCTTCAGTGCTATTG
TGAATGAGTACATTGAAGCCATGAAGAAGCTCGCATGTGAGATCCTGGACCTGTTAGGAGAGGGGCTAGG
TCTCAAGGACCCCAGATACTTCAGCAAGCTTACCACAAACGCTGACAGTGACTGCCTCCTGAGGATCAAC
CACTACCCTCCATCATGCAACATTCACAAACTTGACCATGATGACCAATGCAATATCAAGAGCCTTGTTA
GCACCAAGGCTAGCAATGGTGGGAATCTGATGGCAGGTGGGCGCATTGGGTTCGGCGAGCACTCTGACCC
GCAGATCCTTAGCTTGCTCCGAGCAAACGATGTGGAAGGGCTACAGGTGTTTGTGCCGGACCACGAGGGC
AAGGAGATGTGGGTTCAGGTGCCATCGGACCCATCGGCCATTTTCGTCAATGTTGGTGATGTCCTCCAGG
CTCTGACAAATGGGAGGCTGATAAGTATCCGGCACAGGGTAATTGCAACCGCCTGCAGGCCAAGGCTGTC
CACAATATACTTCGCATCACCACCCCTGCATGCACGAATCTCGGCACTCCCAGAGACAATCACAGCCAGC
AGCCCACGCCGATACCGATCATTCACCTGGGCTGAGTACAAGACGACAATGTACTCACTCCGCCTGAGCC
ACAGCCGCCTAGAACTCTTCAAAATTGACGATGATGACAGCGACAATGCCAGTGAGGGAAAAGCATAGGA
ATTGCTGGTTAAATTGCAGACGATGCCTATGGACCAGTGGGGATTAGGAAGCTGAAACTGTCCCCAAAAT
TTTGGCTCTCTGGCAGTCTGGCTACTATCGTCAGATATCTCACTATTATGATGGTGTAGTGCCTAAGTTG
ACGGGTGTGTAATATCGTTAGCAGTCTACAGAAGCTATGGTTGTACGGAAGTAATGTACTGTCGCCTTTT
CAGCTAACTATCCATGTTCTCTCTTATATGTAATGAGTTAGTTGACGGATGTGTAATATTGCTAGCATTG
TATATAAGCTATGGTTGTATGGAAGTATGTAATATAGCCTTTTCAGCTAA

>*OsGA2ox2* LOC9266994

GTCAAATGCAGAACCTATGGTCAGCATTTCCACAGGAATCTCCACATGAACACATATGACAACCAATTCC
TTATATATATCGCCTTTACCAACAATGCCACCATCTGCACAACAGCACAAGTGCAGGAGCAGCATCAGAA
GCAGCCACAGCAGCAACATTAACGCCATGGTGGTGCCGGCTGCTGCTGCGCCGGAATGCGGCCGCCGGGA
GGCGGCGGCGGCAGCTGCGGCCGCCGTGTTCTGTCGGCGCGGCCGGGGCGTCGTCGTCCCGACGGTCGAC
ATGTCGGCGCCGGCGGGGCGCGGCGAGCTGTCGCGGCAGGTGGCGCGGGCGTGCGCCGGGAGCGGCTTCT
TCAGGGCCGTCAACCACGGCGTGCCGCCGCGGGTGTCCGCGGCGATGGACGCCGCCGCGGCGGCGTTCTT
CGTGCGGGCGGGGGCCGAGAAGCAGCTCGCCGGGCCGCCCGACCCGCTGGGCTACGGCAGCCGGAGCATC
GGGGCGAACGGCGACGTCGGCGAGCTGGAGTACCTGATCCTGCACGCGAGCCCCGACGCGGTGGCGCGCA
AGGCCAGCGCCATCGACAGGGAAGACCCTCGACGGTTCAGCCAGGTGGTAAATGATTATGTGGAGGCAGT
GAGGCAGCTTGCTTGCCATGTCCTTGACCTGCTAGGAGAGGGCCTAGGCCTCAGGGACCCCACATCCCTG
ACAAGGCTCATCACAGCCACTGACAACGACTCCCTCATCAGGATCAATCACTACCCTCCATCCTGCGCCG
CCGCCGCCGGCGACCACAAGTCCGGCGGCGGCCCGGCGCCGACGGCGGCCATCGGGTTCGGCGAGCACAC
CGACCCTCAGATCCTCAGCGTCCTGCGTGCCAACGACGCCGACGGCCTGCAGCTGCTTCTGCCGGACGCC
GCCGCCGCCGGCGACAGCGTCTGGGTCCCCGTGCCGCCCGACCCGTCCGCGTTCTTCGTCAACGTCGGTG
ATCTCCTTCAGGCTTTGACAAACGGGAGGCTGGTGAGTATCCGGCACAGGGTGGTGGTCGGCACCGGCAA
GCCGAGGCTGTCGACCATCTACTTCGCGGCGCCGCCGCTGCACGCCAGGATCTCGGCTCTCCCGGAGACG
GTGGCCGCCGGCGCGCCGCGCCGGTACAGGGCCTTCACCTGGGCGGAGTACAAGAGGACCATGTACACGC
TCCGCCTCAGCCACAACCGCCTCGACCTCTTCCACGCCGGCGACGGCGACGGCGATGCCGGCGTTGGGGA
CGATGACGACCATGAATAGCTGGACATGAGAGATCTTGGAACTGTGCAATGCACACGAAATTTTGGATCG
CTGGAAGTTTGTTGCAATTTCGTTGGATTGTAATATATTTATGGTGCAGTAATTACTAATTAAGTTTGTG
ACTTTGTAGTAACAGTGATCAGTGTCCTTGTGCATTAGTGCATATTGTATAGGCTACAGCATAATTATAT

GTTCTGTTTCAAATTTTTGTCCTAAAGTCAAATATTCATAACTTTGATTCATTGATTTCTAAAAATTTAT
TTAGTTTAACATGATAAGATTCATGTTTTTAGATTA

>*OsGA2ox3* **LOC4325145**
CACTTCTCCCCCACATGCACTTGCAGTCAGTGGTACAACACCACAGCAACGCACCACCGGCCATCAACCT
CCTCTCCCCTGCTTCTCGCTCGCTCTCCTGCCCTGTTTCTTGGAGAAGTGAGCTTAAGTGAGAGCCTCTG
TCCCTCGCACACGCTATTGGCCATGGTGGTTCTCGCTGGCCCGCCCGCCGTCGATCACATCCCGCTGCTG
AGGTCGCCGGACCCCGGCGACGTCTTCTCCGGCGTGCCGGTCGTCGACCTCGGCAGCCCCGGCGCGGCGA
GGGCCGTGGTGGACGCCTGCGAGCGGTACGGGTTCTTCAAGGTCGTCAACCACGGCGTGGCCACGGACAC
GATGGACAAGGCCGAGTCGGAGGCCGTCAGGTTCTTCTCCCAGACGCAGCCCGACAAGGACCGCTCCGGC
CCGGCCTACCCGTTCGGGTACGGCAGCAAGCGGATCGGGTTCAATGGCGACATGGGGTGGCTCGAGTACC
TCCTCCTCGCCCTCGACGACGCGTCGCTCGCCGACGCCTGCACCGTCCCGTCCTGCGCGGTCTTCCGGGC
CGCTCTGAACGAGTACATCTCGGGGGTGCGGAAGGTGGCGGTGCGGGTGATGGAGGCGATGTCGGAGGGG
CTGGGCATTGCGCAGGCGGACGCGCTGAGCGCGCTGGTGACGGCGGAAGGGAGCGACCAGGTGTTCCGCG
TGAACCACTACCCGCCGTGCCGCGCGCTGCAGGGGCTCGGCTGCAGCGTCACCGGCTTCGGCGAGCACAC
CGACCCGCAGCTCGTCTCCGTGCTCCGCTCAAACGGCACGTCCGGCCTGCAGATCGCGCTCCGCGACGGC
CAGTGGGTGTCCGTGCCCTCCGACCGCGACTCCTTCTTCGTCAACGTCGGCGACTCGTTGCAGGTTCTGA
CCAATGGGAGGTTCAAGAGCGTGAAGCACAGGGTGGTGGCCAACAGCCTAAAGTCTAGGGTTTCCTTCAT
CTACTTTGGAGGGCCACCGTTAGCACAGAGGATTGCACCATTGCCACAGCTGCTGGGGGAGGGAGAGCAG
AGCCTGTACAAGGAGTTCACATGGGATGAGTACAAGAAGGCTGCCTACAAATCAAGGCTTGGAGACAACA
GGCTGGCCCAGTTTGAGAAGAAGTAGCTAGCTAGATGCCTAGCAACAGAACTGGCCGGTCAAGCAACAAC
GACTTTACTGCATGGCAGCTAGCTACCTTAGCTATGTCTCACGCACACGTACGGTACACCATGACCACGA
GAAGATGAAGCTGCTTAAGAGAGAAAAAGAAAAGAAAATAAAAACAGGAAGAAGATAATAACTTCAAGAT
GCACAATGTACAGCCAGGTTGGATGGATAGCTAGATTTTTCTCGTGTGGCTAATCACCGTGTGTGGTGAG
GTGGGCTCCTTTCCTGTTTCGGAATACTGATCGATGGATGAACAGCCTCCAATAGTTAATGTTAACCAAA
TATAGCACAACAAAGGGGTTCTTTTCTTCCTTTGGTTGTTTCTTTTTTCCTCTTGCCTTTGATTTTCTCG
TCATGCTTTCTGAGCATTAATTATGTGTGCGTGTATTTCGGTATACGTAACGTACTTTTACCTATGTGTG
TATAGTACGTGGCTTGTGTTAACAAGTAAATATTATAGTCTCTCTCTTTCGTTA

>*OsGA2ox4* **LOC4339310**
ACCTCGTCTCGATCCCCCCTATATATACACCATGCACTCATCACCTCGACCTCAGCTCAGCTCACTCATC
GGTAGCAGTGTGTACTCTAGCTCGCTCGATCTTGGCCATGGTGGTGCTCGCGAAGCCGGCGGCGCTGGAG
CAGATCTCGCTGGTGAGGTCGCCGTCGGTGGAGGATAACTTCGGCGCCGGGTTGCCGGTGGTGGACCTGG
CCGCGGACGGCGCGGCGGGCGAGGTGGTGCGGGCGTGCGAGAGGTTTGGGTTCTTCAAGGTGGTGAGCCA
TGGCGTCGGGGAGGGCGTGGTGGGTAGGCTGGAGGCGGAGGCCGTCCGGTTCTTCGCGTCGCCGCAGGCG
GCGAAGGACGCGCACGGCCCCGCGTCGCCGTTCGGGTACGGGAGCAAGCGCATCGGCCGCAATGGCGACA
TGGGGTGGCTCGAGTACCTCCTCCTCGCCATCGACGGCGCGTCCCTCTCCCGTTCCTCCCCCGCACCGTC
GTCCTCGCTCCGGGACGCGGCGAACAAGTACGTGGGCGCCATGCGGGGGATGGCGAGGACGGTGCTGGAG
ATGGTGGCGGAGGGCCTCGGCGTGGCGCCGCGCGGCGCGCTCGCCGACATGGTCGTCGGCGACGGCGCGG
CGAGCGACCAGATCCTCCGGCTGAACCACTACCCGCCATGCCCGCCGCTGCTGCAGAACCTGATGCCCAA
CTGCAGCCCGACCGGGTTCGGCGAGCACACCGACCCGCAGCTCATCTCCATCCTCCACTCCAACTCCACC
TCCGGCCTCCAGGTCGCCCTCCACCACGACGCCGACGCCGGCGACCACCAGTGGGTCACCGTCCCTCCCG
ACCCCGCCTCCTTCCTCGTCATCGTCGGCGACTCGCTGCAGGTGATGACGAACGGGAGGATGAGGAGCGT
CCGGCACAGGGTGGTGGCCAACAAGCTCAAGTCGAGGGTGTCAATGATCTACTTCGGAGGGCCGCCATTG

GAGCAGAGGATCGCGCCGCTGCGGCAGCTGCTGGTGGCCGGCGTCGGCAACGGCGAGGAGGAGGAGCAGA
GCCGGTACGAGGAGTTCACGTGGGGGGAGTACAAGAAGGCTGCCTACCTCTCCCGCCTCAGCGACAACCG
CCTGGCCCCCTTCCACCGGCAGCCACCACCTGTCGCTAACCCACTCGCCTAAGCTAAGTTAAGGGTGTAA
GTGGTTAAACCGTGAAACTCACTTATAGATAAAAGTAAGTTTCGAACCCACTTACTTTAACCTATTAGTG
GGTTTGCGGCTTACCCACTTACACCCTAAAGCTGAGCTAGGCTCCTCGCTTACACAGATCGACACTGCAT
TGAGAATGACGACGAATTAAGCTACTGATCAACCTAACACCAATCGATGATTGATCGTCCATCGATTCGA
TCCAGCCATGATCCGTGCAACAAGGGGACTGGGGCCAGCATGGTAGCTAGATAAGGGTAAGGAGGGTAGG
GCATATGTGGGGCCATGGATGCAACTTTTAATTCTACTATAGCTCAAATAGAGACAACATGCTTATTATC
TATGCATATGCAGCTCCAATCAATTGGATAATTTGCTAGATCCTCTTTCCTTTTGGATGATTGATTGCCA
AAATGTACAGTTGTCATCACTACATGATGTACAGTGTAGGGTTTGTCACCAACCAGTGCTAGCTAGCTAG
CTAAGCTAGTAAAGCCAAAAAGCTTGTTGTTGGGTGTGTGAAACTATGCAAGTTTGTGGGTTGTATTTTT
ACTCATGTATTGATTTGACTTTGTGTGTA

>*OsGA2ox5* LOC4342182

AGATACACTGCTGCAGTACAGAGGAGGCCGGAGGAGGAACTCCAAGCTAGAGGATGCGCGGGCCTCAACG
TTCAGTGGCGGCACGCCACACGTTTCATCTCCTAGCTAGCTTGTTGTACTGCATCTGTAATATACTCCTT
ATATTCCTTCATTTTTCAGCCTCGATCAGCTCTCCTGGGGTTGGTTGGTTGGTTGGGTTATATATCTACC
AAAGCATGCATATCCCTTCCTTCTTCCACTGTGTTCCTAATTCCTTCCCTGCAATTATTGGTTGCTGCTT
CACTTGATCTTATTGGCAACCAGCTAGCTAAGCTTATACACACCCACGGCCAGACCTGAACCTTCTACCT
ACTACCGCACAAAAACAACAAGCTACCTAGCTCTCTAACAACAATCTCATTTGCATGAATCGCAGGAGAT
CGATAGTAGCTACTAGTTAGCAAGTTCATATATAGATCGATCTATGATCGATGCTAGCTAGCTAATAGTA
TCATCGATCGGGAAAACAGACACAAATTAGAGCTAGCTAGAGCCCCCGCCGGCCGGGTCGACGTACTGCT
GCTAATCAATAGCCAGGCAGGGCGGAGTTAATAATTAATGGAGGAGCACGACTACGACTCCAACTCGAAC
CCGCCGTTGATGAGCACGTACAAGCACCTGTTCGTGGAGCAGCACCGCCTGGACATGGACATGGGCGCCA
TCGACGTCGACGAGTGCGAGCTCCCTGTGATCGACCTCGCGGGGCTCATGGAGGCGGAGCAGGTGTGCCG
CGCAGACATGGTGCGTGCGGCGTCGGAATGGGGCTTCTTCCAGGTGACCAACCACGGCGTGCCGCAGGCG
CTGCTGCGCGAGCTGCACGACGCACAGGTGGCCGTGTTCCGGCGGCCCTTCCAGGAGAAGGTGACCGAGA
GGCTGCTCGGCTTCTCGCCGGAGAGCTACCGGTGGGGAACGCCGACGGCCAAGTGCCTGGAGCAGCTGTC
GTGGTCGGAGGCCTATCACATCCCAATGACGACGCCCAGGCCCAGCACGAGCATCAGGGCCAGGGCGGTG
ATCGAGGAGGTGTCGAGGGCGATGTACGAGCTGGCGCAGAAGCTGGCAGAGATCCTGATGAGAGGGCTGC
CGGGCGCCGGCGAGGGCGAGACGATGGTGACGACGCGGGAGGAGACGTGCTTCCTGCGGCTGAACCGGTA
CCCACCGTGCGCCATGGCCATGGGGGGCTTCGGGCTGTGCCCGCACACGGACAGCGACTTGCTCACCATC
GTGCACCAGCAGCAGGACACCGTCGGCGGCCTCCAGCTGCTCAAGGGCGGCAGGTGGGTGGCCGTGAAGC
CCAGCCCCAGCACCCTCATCGTCAACGTCGGCGACCTCCTGCAGGCGTGGAGCAACGATGTGTACAAGAG
CGTGGAGCACAGGGTGATGGCCAACGCCACGCTGGAGCGCTTCTCCATGGCCTTCTTCCTCTGCCCCTCC
TACCACACGCTCATCATCCCAAGCAGCAGCCATGTCCACGACGACGATGCCCATTACCGGAGCTTCACCT
TCGGCGAGTACAGGAAGCAGATCATGGAGGACGTCAGGAGCACAGGCCGCAAGATTGGACTGCACCGCTT
TCGAACCCGATAGATAGATCCACCTGACCTACATACCCGCCCTACTCCTACTATATATATCAAATCACCA
CTGCTATAATGCATTATACTCGATCCACTATGCATTTTTCATCGCCACAATATGCATGTACCTAGCCAAT
AATTAATTAAGCTGCACTTTGCTTACTTATTGCTAGCTTGCTACTGCATCGATCTATATGCATGTACACA
CATTTCATTGCCTTAGCAGTGTATGTATATGCATTATTTTTTCCTTATTTGCCCTTAGCTTGCAGATTTC
ATAGCAGCTAATATATATCCGTCGGAATTATTTGAACATTCATTGTGACTTTTCTTTTCAATGTGTACGT
GGCTCCAAAATGATCATAGTTTAAACAAGCACATACATGTATATATTTGAACACTGGAGGTTCTTCTGAA
TCGGAGTATTTGGTGACA

>*OsGA2ox6* LOC4336431
CTAGCTAGTAGTATCCCAGCAGCATTCGTCGACGCGCGCTCTGCGATAGCCAGAAGCTAGCTTAGCATAC
GTGCAACTGCGTGTGCGACGCGTACACGGCTGTACAGACACAGATAAGCCTCTCGCCTACGTAGACGTCT
TCCCGACCATGCCGGCCTTCGCCGACATCGCCATCGACCCGCCTCTGGCCGACAGCTACCGCGCGCTGGC
GCTGCTCCGCCGCGACCGCGACGGTGGCATTGCGCCGCCGGCTGTGCAGATGGTCGGCTCGGGCGGCGCC
GTGCTGGAGCGCGACCTGCCGATGGTGGACCTGGAGCGGCTGACGAGGGGCGGCGCGGGGGAGAGGAAGG
CGTGCGCGGGCGCCATGGCGAGGGCGGCGTCGGAGTGGGGGTTCTTCCAGCTGACCAACCACGGCGTGGG
CCGGGAGCTGATGGAGGAGATGAGGCGGGAGCAGGCAAGGCTGTTCCGTCTGCCGTTCGAAACCAAGGAG
AAGGCCGGCCTGCTCAACGGCTCGTACCGGTGGGGCAACCCCACCGCCACGTCGCTCCGCCACCTCTCGT
GGTCGGAGGCGTTCCACGTCCCGCTCGCCAGCATCTCCGGGGCGGATTGCGACTTTGGAGACCTCACCTC
CTTAAGGGGCGTGATGCAGGAGGTGGCCGAAGCGATGTCGCGGGTGGCGAACACGGTGGCAGCGGCGCTG
GCGGAGGAGCTGACCGGGCGCGGAGGCGGCGGGGCATCGGCGGCGCCGTGGTTCCCTGCGGGGTGCGACG
AGACGACGTGCTTCCTGCGGCTCAACCGGTACCCGGCGTGCCCTTTCGCGGCGGACACGTTCGGGCTGGT
GCCGCACACGGACAGCGACTTCCTCACCGTCCTGTGCCAGGACCAGGTCGGGGGCCTGCACCTGATGAAG
GACTCCCGGTGGGTGGCCGTCAGGCCACGCCCCGACGCCCTCGTCGTCAACATCGGCGATCTGTTTCAGG
CGTGGAGCAACAACAGGTACAAGAGCGTGGAGCATAAAGTGGTGGCCAACGCCAAGACGGACCGGCTATC
GGTGGCCTACTTCCTGTGCCCGTCCTACGACTCGCTTGTCGGGACATGCGGCGAGCCATCGCCATACAGG
GCCTTCACCTTCGGGGAGTACAGGAAGAAGGTGCAGGAAGACGTCAGGACAACCGGGAAAAAGATTGGCC
TCCCAAACTTTTTCAAGCATTCTTCAGTACAATAATGATCGCATCAATGACAGCAACCGCTTGTTCTATA
TATGTTCGTTATAATTTATGGTCGATGTGAACTCGACCGTACCATCAATCCATCTCACTGTACAATTGTG
TGTGCGGTGTTCGGGGTTGGAGGTTCTTTCCTTATCATTTCCGTGGCCTTTTTTGGTTTGTAGATTGTAA
TTGGCAAGGTAGTACTATACAAGTGATTAAACCAGAAGGCCTTATGTGCAATTATCAAGTTATCGCATAA
TGACAACTGCCACCATGTCGGAGGAAATAA

>*OsGA2ox7* LOC4325537
TCGTATGATCGTATCAAACTCAACCATCCTTGCTTTGGCTGCGTAGCGTGTAGCGCGCGCGAACGAACGA
ACACACCAAGCTTTGCTCTGCTTTTCCTCTGTCCAAAACAGCCGGCCATGGTGGTGCTTGCCAAGGGCGA
GCTCGAGCAGATAGCCCTGCCGGCGGCGCACCCGCCGCCAGCCGACGTGCGCGCGATCGACCTGTCCGCC
ACGGGTCCCGCCCGCGCGGCGGAGGCGCGCGCGCTGGTGGCGGCGTGCGAGGAGCAGGGGTTCTTCCGGG
TGACGGGCCACGGCGTGCCGCCGGGGCTGGTGCGCGCCGCGGAGGCCGCCGCGGCGCGGTTCTTCGCGCT
GCCGCAGCCCGACAAGGAGGCCGCCGCAGGGGCGCCGCTCGGGTACGCCAGCAAGCGGATCGGCAGCGCC
GGCGACCTCGGCTGGATCGAGTACCTGCTACTCTGCCTCGCCCCCGCCGCCGCCGCCGCGGCATTGCCGT
GCGCCGCGACGTCGCCCACGCCTCCTTGCCCCTTACGGGAGCTTCTACGCGAGTACAGCGCGGCGGTGCG
GCGGGTGGCGTGCGGCGTGCTGGAGCTGATGGCGGAGGGGCTCGGCGTCGGGCCGGCGGACGCGCTGGCG
CGGCTGGTGGCGCGCGAGGACAGCGACTCCATCCTCAGGGTGAACCACTACCCGCCGCGCCCCGATCAGC
TGGGCGGCGGCGGCGGGCCGAACCTGACGGGGTTCGGCGAGCACACCGACCCGCAGATCATCTCCGTGCT
CCGCTCCAACGGCGCCCCCGGGCTGGAGATCTCCCTCCGTGACGGCGCCTGGGCGTCCGTGCCGCACGAC
GGCGACGGCGACTCCTTTTTCGTCAACGTCGGCGACACCCTCCAGGTGCTAACGAACGGGAGGTTCAGGA
GCGTGAAGCACAGGGTGGTGGTGAACAGCGAGAAGTCGAGGGTGTCCATGGTCTTCTTCGGCGGCCCGCC
GCCCGGCGAGAGGCTGGCGCCGCTGCCGGCGTTATTAGGGGACGGCGGCCGGAGCCGGTACAGGGAGTTC
ACCTGGAAGGAGTACAAGGGCAGCGGCTGCAAGGGCCGGCTCGCCGACGACAGGCTCTGCAGATTTGAGA
ACTAGTAGTAGCAGTCATCTGACTTCTATGTACGTTTGTGTATATTAATTCGCAAGTAGTGATTATCACT
GTGGTTTGCCTCTAGCAATAGCCTTGTACGTAAATTTCAGTATGTGTTAATCTGTTTATATATAAGCTTC
CAGCAAAAGTTGCATGCATGTAAGTGCTCTGTGGACAGTTTAGTTTTATTACAATTAAATGAGCTCTCTT
AAGATGA

>*OsGA2ox8*   LOC4339600

GCTGCCTATATATACGAGGCCATCTGACGAGCAGAACACAAGTGCAAACGTGCAGCAGCAGAGGCAGCAA
CACCTCCCCTGTTCTTGCTCTGTTTCCTCACTCTGTTGGTACACAACAAAAAGAAGAAGCTTAATTAGGT
GGTTTTTGATCTGATCACGCGCCATGGTGGCGATCACGGCGCCGAGCTCCATCGAGCACATCCCGCTGGT
GAGGTGCCCCAAGGGCGCCAATGCCGGGCCGCAAGCTGTCATCCCGTGCATCGACCTGTCGGCACCGGGC
GCGGCGGCGGCGGTGGCCGACGCGTGCCGCACCCTGGGGTTCTTCAAGGCGACCAACCACGGCGTCCCCG
CGGGGCTCGCCGACGCGTTGGAGTCGAGCGCCATGGCGTTCTTCGCGCTCCCGCACCAGGAGAAGCTCGA
CATGTCCGGCCCCGCCCGGCCCCTCGGCTACGGCAGCAAGAGCATCGGGTCGAACGGCGACGTGGGGTGG
CTGGAGTACCTCCTCCTCTCGGCCGGCGCCGCCTCGTCCGGCGGCGCGGCGCTGCCGGCGGCGCTGAGGG
CGGCGGTGGAGGCGTACACGGGGGCGGTGAGGGGGGTGGGGTGCAGGGTGATGGAGCTGATGGCGGAGGG
GCTGGGGCTGGGGGCGTCGGAGGAGGGGAGGTGCGTGCTGCGGCGGATGGTGGTGGGGTGCGAGGGCAGC
GACGAGATGCTGCGGGTGAACCACTACCCGCCGTGCCTCCTCCCGCCGGGCCGCGACCGGGACGAGTGCG
GCGTGACGGGCTTCGGGGAGCACACGGACCCACAGATCATCTCCGTGCTCAGGTCCAACTGCACCGCGGG
CCTCCAGATCCTCCTCCGCGGAGACTACTCCTCCCCTGCCCGCTGGGTCCCCGTGCCCCCCGACCCCGAT
TCCTTCTTCGTCAACGTCGGCGACTCCCTCCAAGTGTTGACGAATGGGAGGTTCAGGAGCGTGAAGCACA
GGGTGTTGGCGCCGGAGGGGGAGGAGTCGAGGCTGTCGGTGATCTACTTCGGCGGGCCAGCGGCGTCGCA
GCGGATCGCGCCGCTGGAGCAGGTGATGCGGGAGGGGGAGCAGAGCCTGTACAGGGAGTTCACCTGGGGG
GAGTACAAGAAGGCCGCCTACAAGACGCGCCTCGGCGACAACCGCCTCGGCCCCTACGAGCTGCAGCACG
CCGCTGCCAACGATGAGGCCGCGACGAAGAAATAAATCGATCAACCCGACGACTGTGAACAACTGTGAAG
TGTTACTGTACTAGTGTAGTTAGCTCCGTGGTACTGGTAGCTGTGAATATGATCGAGATCGATCGATCGA
TCGATCTATCCTTGTGTTGTGTTACTTTTAACCCATTTCTGGTAACTAAAGGCCGCCGTTGCCTTGGCTT
GCTTGGCCAGCCCAAAACTGGCACTGGCGAGCTGCCTCCTCCTGCATGCCTCTGCTGTGCAGTAGTGCAG
CAGTACTCCTCTGTCCTGGGGTTTTCTGCAGCAGAGCAGTGGAGTGCAGTTCAGTGCAGCGTTGTGCATG
TTGTGTAAAATAGCTTGCCGCATGCATATGTGATTATGTGCTGCTCCTGTCGACGCTGCCGCCCCTTTTG
TAGCATGGTTTTTACCGGCTGGGCGGTCGGCAGGTGCAGTAGTAGCTAATGATGCTTACTGCGCCATACG
AGTGTACCTATATGTGTGCTATATGTGTGCTACCTCCGTTCCAAAATAAGTGCAGTCGTGGATAT

>*OsGA2ox9*   LOC9266251

TAAGCCTAGCTTGCCTTGCTCCTCCTCGCATCCACCCCCAAGCTCGCGCTCAACCAAACACACTACAGCT
AGCTTATAGCTAAGCTGTGTGTGCTAGCTCGATCGGTAGTAACAACGTCCCAGGCAGCCATGCCGGCCAT
CGCGGATTGCGCGGCCGACCCGCCGCTGGCGGACAGCTACTACACGCTGCTCCGCCTCGGCGGGGACGAC
GACGACGACGCGTGCACGAAGGTGACCACGACGCCGCAGCCCGTGTCGGAGTGCGAGCTCCCGATGATCG
ACGTCGGTTGCCTGACGGCGCCGACCGGCGCCGCCGCCGCCGCCGCCGTGGGGCAGCAGCATCAAGCCGA
GGAGAGGGCGGCCTGCGCGGCGGCCATCGCGGCGGCGGCCGCGGAGTGGGGGTTCTTCCAGGTGGTGAAC
CACGGCGTCGCGCAGGAGCTCCTGGAGGCGATGCGCCGGGAGCAGGCGCGGCTGTTCCGCCTCCCGTTCG
AGGCCAAGTCCAGCGCCGGCCTCCTCAACGACTCCTACCGCTGGGGCACCCCGACCGCCACCTCGCTCCG
CCAGCTCTCCTGGTCGGAGGCCTTCCACCTCCCGCTCGCCGGCATCTCCGGCAAATCCTGCAACTACGGC
GACCTCACCTCCCTCAGGGACGTGACGCGGGAGGTGGCGGACGCGATGTCGAGGCTGGCCAGGGCGCTGG
CGCGCGTGCTGGCGGAGAGCCTCCTGGGCCACGCCGCCGGCGAGCGATTCCCGGAGGGGTGCGACGACGC
GACGTGCTTCCTCCGGCTGAACCGCTACCCGCCGTGCCCCTTCCCACCGGACGACGCCTTCGGCCTGGTC
CCGCACACCGACAGCGACTTCCTCACCGTGCTCTGCCAGGACCACGTCGGCGGCCTGCAGCTCATGAAGG
GCTCCCGCTGGGTCGCCGTCAAGCCCATCCCCGGCGCCCTCATCGTCAACATCGGAGACCTTTTTCAGGC
GTGGAGCAACAACAGGTACAAGAGCGTGGAGCACAGGGTGATGACGAACGCGACGACGGAGAGATACTCC

GTCGCCTACTTCCTCTGCCCGTCGTACGACTCGCCCATCGGCACGTGCAGGGAGCCTTCCCCTTACAAGG
CGTTCACCTTCGGGGAGTACAGGCGAAGGGTGCAGGAAGACGTCAAGAAGACGGGGAAGAAGACTGGCCT
CAGTAATTTCCTCGTATGAGGCGCACACAAATTCAGTGGCCGGCCAGTTTCAGTTCCGTTCTGTGTATCA
TCAGTAAATGTACAATTATTAATGACTAAGAGTGCAGTACATTAATTAATTAATTAATTAGTTTATTGTA
GCTTAGTTTGACGGTGTACAAGTACTGGTAGGAGATTGCCGGTATATATGTACGTAGGAAATAAGATAAG
GGATCCATGTACAGTTTATGTACGGCCTTCGATCCCTTGTCCTTTGGTTTGAATTATGATGTTTCTTAAA
CGTGCTGTATGTATGTTGATGAATAACTAGTATTTCTTTAGATTTGTAAGAAA

>*OsGA2ox10* LOC4335758

GTTAGTAGATAGCTTTGCTCTCTCTCTTTATTTAATCTCTTCAAAGTAGAAAAATATGCTGACATGAATC
TCTTATAGAGAGCCTATAGATAACCATTGCGGGTGCCCTTATGAATTGGGTTTCTCTTTTCTTCTCAAGA
GTTCCCTTTGGCTTCCCACCCCTCCTTTTCTACTCCCTTCAAGAACATTGTGCTATACATCATTAACTAC
ACTGAAATTCTCAGCATCATTCCCTTTGCTGAATGGAGAAGTCCGTAGAATCTGCTGATCTGCTCTACAT
CATCAATTTTTCAAGCTCGGTTTTCAAACATCTGTACCGCACCGTCCGAAATTTCACTTTTGGCAGCAAT
TGAATGGCCTTTTGGCAGATCCTATCATACTGCAAAACGAGTAGGGCAGAAACGACTACGGGCCGGGCCT
GGCCGCCATCTCACTAATGGGCTGAGATGGAGGCCGTAGGCTTCTGATGGGCTGGGCCTCAGCTGCCGCC
ACGTGCGGCAGGCTTCAACCAGGAGGAGGTGGGGGCGAGTCGTCGTCAACGACGACGACGAGGACCACGC
AGATGGACCCGTCGTGCCCGCCGTTCCCGCAGCCGGAGGCGCCACCGCTGCCGCTGCAGCTGCAGCTGCA
GCCGGGTCTGCCCAGGCTGGAGCTGCCGACGCTGGACCTGGAGCGCGTCGGCGGCGAGGACCGGGCGGCG
CTGGTGGCGGCCTGCCGGGATCTGGGCGCGTTCCGCGTGGTGAACCACGGCGTCCCCGGCGAGCTCCGGC
GGCGGCTGCTGGAGCTGGGGAAGCAGCTGCTGGGTCGCGACACCTTCGAGCTGAAGAAGGCGCGGCCGGG
CTACTTCTGGGGCACGGCTGCGCTGAAGTCGCTCCGCGTCAAGGAGGTCAACTGGCTGGAGGGACTCCAT
GTCGATCTCGTCCCCGGCTCGTCGTCGTCGTCGTCGCAGGTCGGCGATGGCGATGATGATGATGATGATG
GCTGGATGCGGATCAGGGCGCTGATGGCGGAGTACGGCGACCACATGGCGCGCATCGCCCGCAAGCTGTT
CGACGCCCTGGCCGCCGAGCTGGGCCTGGATCATCACCAGGCGGCGTCCTACCTCGCCGAGCGCCAGGGC
TTCCTCCGCCTGTACCGCTACCCGCCCTGCCCTTCCTCCGCCTCCTGCCTCGGGATGGAGCCCCACACCG
ACAGCTCCGTGCTCTCCATCATCCTCGGCCAGGACCACGTCGGCGGCCTGCAGGTGATCCGCGACGGCGC
GTGGCGCGACGTCGCCCCCGCGCCCGGCGAGCTCCTCGTCAACCTGGGCGACATGATGACGGCCATCAGC
GGCGGCTCGTACCAGAGCGTGCGCCACAGGGTGCTCGCGAGCCGCCCCTCCACGGAGAGGGTGTCGTGCT
GCTACTTCGCCTTCCCGCAGGAGGACGCCGTCGTCGAGGCCCCCAGCGGCATCGGCGGCGGCGTCTACAG
GCCCTTCTCCTACAGGGAATTCCGCGAGCAGGTGCAGGCCGACATCAAGGCCGTCGGCACCAAGGTCGGC
CTCAGCCGCTTCTATGCGACGGCTACCAGGTATACCAGGTAGTAACAAGGTAGAAGATGTGCTGCTGCAT
TGTGTAGGATATTGTAGTACAAGGTCCATGGTACCAATTGGTATTTGCAATGATTTTCTGGCTAATTGCT
TGTCTGAATAAAAGGAGTATACATGGCATGATAATAAATTTTCTAGCCAACATTACATGTAAAGATAAAA
GGGAGAAATGATTGAAATCAATACTCAATACTATATTTTAAGATCATCGCATCATTTTACATTAA

4、 Populus

>*PtrGA20ox1-1* LOC7454460

TCTATCACCTAATTTTCTCATTAAGCAACCCCTTGCGCTGCAAATCCCCTCCTCTTCTCAAGCCCTTCCT
TGCAAAAATAAAATCCACACAGCATATAAGAGTGAGGGAAAGATAGCTAGCTACAAGATTCCAACATTCG
GGCTCACCGATTTCCCCAATGAGAGCGGTCTTGGCTTTTCTTTTATCATCATCGGTACACTTCTTTCTTT
TATCTTAACTAGGAGCTAAGTACCAACTCTCCCTCCCTGCCTCTCAAACCTCACACGATCACAATCACAT
ACATACATACATACATACATACATACATATATATATATATACACACAGACCGTGCCCTGCCACAAAATTTGTAA
TGCAATGGCAATAGATTGCATCAAAACCATGCCATCCATAACCACCCCTCATCACCACCCAAAAGATCAG
GATCAATGCAAAGATGATGGCAAGTCTTTTGTTTTTGATGCACAAGTTCTTCGACACCAGACAAACATAC
CCCAGCAGTTCATTTGGCCTGACCATGAAAAGCCTAATATTAATGCACCTGAACTCCAAGTCCCACTTGT

AGACTTGGGTGATTTCCTCTCTGGTAACCCTGTTGCTGCAGTGGAAGCTTCAAGACTTGTTGGTGAAGCA
TGTCAAAAACATGGTTTCTTTCTAGTTGTTAATCATGGAGTTGATAAAACGCTCATTGCCCATGCTCATA
ATTACATGGACACCTTCTTCGAATTGCCACTTTCTGAGAAACAAAAGGCCCAAAGAAAGATTGGCGAGTC
TTGTGGATATGCTAGCAGCTTTACTGGCAGGTTTTCCTCTAAACTTCCATGGAAAGAAACGCTTTCTTTT
CGCTACACAGCTGAGAAGAATTCATCAAAACACATCGAGGAATACTTTCATAACAGAATGGGGGAAGATT
TCGCTGAATTCGGGAGTGTGTATCAGGACTACTGTGAGGCCATGAGCACTTTGTCACTAGGGATCATGGA
GCTATTAGGCATGAGCCTTGGTGTGAGCAGAGAACATTTCAGGGAATTCTTTGATGAGAATGATTCAATA
ATGAGGCTCAACTACTACCCTCCATGCCAAAAACCTGACCTTACTTTAGGCACCGGTCCTCATTGTGATC
CAACTTCTTTGACCATCCTTCATCAAGACCAAGTGGGTGGTCTTCAAGTGTTTGTGGACAACGAATGGCG
TTCGATTAGCCCCAATTTTGACGCTTTTGTTGTTAACATTGGTGACACCTTCATGGCTCTATCGAATGGT
ATATACAAGAGCTGTTTGCACCGAGCAGTGGTGAACAGCCAAACACCAAGAAAATCCCTCGCTTTCTTTT
TGTGTCCAAAGAATGACAAGATGGTAACTCCACCACATGAATTAGTGGACACATGCAATCCGAGAATATA
TCCAGATTTCACATGGCCTATGTTGCTCGAATTCACACAAAAGCATTACCGAGCTGACATGAAGACACTT
GAGGTGTTCACAAACTGGCTTCATCAACAAAGTTTTAGCTGAACAAGTGGGTCAAGTGATGTATCCCACA
AATGCACTTGGGGGCATGTTATTGGTCTTTTTCTTGGAAAAAAAAAATGAAAGCGATGTTGATGGGAGAG
GAGAGGAGACGAAAGGGAAAGAAAGGAAGCTGGAAAGAGATGGGAATTTTGTTAAGACTTGGAGATCGAT
CATCATCCGTGCATGAAGAGATCTCAAAAGTAATTAGAGGCCAATTCTTATTTATGTCCATAGATCTGGT
AATTAATTACTAGCTAACTTATTTATTGGTCATGAACAAAAAGTACAATCATAGACCAATAAAGAGTTGG
ATTAGCTAATTTTTTGTATTTTCCTTGATAATATAGAAAAATTGCCAGTAGTAGCATTACATA

>*PtrGA20ox1-2*   LOC7494026

GTATGTGTGTGTGTCTGTTAATGAGGGTAAAATATGAAGATTAAGTAGGGTAGAGTAGACACACTCTAAA
GAGGAATGACGCAACAGTGAAAACAATGAGATGCCAATATTGTTCACTGACCTTCACAAACGACTGGCCT
TGGCTTTTCCCTCCTCTGCCTTCTAACTTCCTATTAAACTCTACTTACCTCTATCCTCTCTCAATGATCC
ACGATCTCCTCTCAATTTCTCTCATCAACTAAACAAGAGATAAGTCCTTCTAGTTCCGATAATATATATT
TTTCTGAAGTACGTGGAAATTGAAATATACACACATATGTATGTGCGTGTGTGTGTGTGTGTGTGTTTAT
CATGATATGCCGTTGATTGTTTCGCTACAAACATGGTAATGCCTCCTACTCTTCCTCCTCCTCGACAGCC
ATTAGTATTTGATGCTTCAATTCTTCAACACCAAGCCAATATACCCTCTCAGTTCATTTGGCCTGACCAC
GAAAAACCATGCCTTGAATCCCCAAAACTTGCAATCCCCCCTATTGATTTTGGAAGCTTCCTCACTGGAG
ATCCTTTGGCAGTCTCAAAAGCTACTCAGCTAGTAAATGAGGCATGCAAGAAGCATGGATTCTTTCTAGT
TGTTAACCATGGAGTTGATTCAAAGCTTATAGCTAAAGCTCATGAGTATATGAACATGTTTTTTGGCATA
CAACTCTCAGAGAAGCAAAGAGCTCAAAGAAAGATAGGAGAGCAATATGGGTATGCTAGTAGCTTCACTG
GAAGATTCTCCTCCAAACTTCCATGGAAAGAAACACTTTCTTTCCGATATTGTGCTGACAATCAGTCTTC
TGACATCGTCCAAGAATACTTCTTGAATGTAATGGGAGAAAATTTCAAACAATTCGGGAAGGTTTACCAA
GAATATTGTGAAGCCATGAACACTCTTTCTCTTGGAATTATGGAGCTATTAGGAGTAAGTCTAGGAGTTG
GAAGAGAATATTTTAGAGATTTCTTCGAAGGAAACGATTCGATAATGAGACTGAATTACTACCCTCCTTG
CCAAAAGCCGGATTTAACTCTTGGCACTGGGCCTCATTGTGATCCCACATCCTTAACAATCCTCCATCAA
GATCATGTCGGTGGCCTTCAAGTGTTCGTCGATGAAAAATGGCACTCCGTCAGTCCTGATCCGGAAGCTT
TCGTCGTCAACATCGGTGACACATTCACGGCTCTATCAAATGGCATTTTCAAGAGTTGCTTGCATAGAGC
GGTGGTAAATAATGTAACAGTAAGGAAATCCCTTGCTTTCTTTCTCTGTCCAAAACTGGATAAGGTGGTG
AAGCCACCAAATACCTTGATCGACTCTAAGAATCCGAGGGTATATCCGGACTTCACATGGCCAACTTTGT
CAGAATTCACACAGAAACATTATAGGGCCGACATGAAAACCCTAGATGTCTTCACAACCTGGCTTCAACA
AAAAAACAATTAGAGGGTACCAAATTTTATTTTTTCCCCTGTTAATTAGGCCGAGGAAAGTGAGATTATA
GGAGTGGACAATGCTAGGTGCAGTGAAGGAGGGACTTGGTTCTTCATGGTGTGTATTTAGAAGAAAAAGG
CAACAGAAGAACCAAAAAAAGAGGCCAGCCCTGTAATTTGCTGGTCGTGTGTTAGTGTTGGCGAGAATCA

TATCAATGCATGTAAGAAGCAAATTGTAGGAATAAAGATTTGAAATGGGCTCCATGCATTTCTTCGTATT
GTTATGCTATTTTCATTTTGTTAACACGACATAGATTCTGTGTACTTATGTGCTTGGCCACCGCCAAATT
CTGGTAGCCTTGATTATCTTTCTATATATATGAAAGATAACAATATTCAGTAA

>*PtrGA20ox1-3* LOC7488922

ACCTCCTCATTCCCCTCTCATAGTTCCATTTTCTCCTCTCAATTTCTCTCATCAACGTCTAAAGTAGATA
ACAGTCCTTCTAGTTCCTATAATATCTTTTTGATCCAACTAGAAATTGAAATACATATAACATTGTATGC
CCATTGATTGTTTCCCTTCAAACATGACAATTATGCCTCCTCCTCCTCCTCCTCCAGAGCCATTAGTATT
TGATGCTTCAATCCTTCAACACCAAGCGAACTTACCCTCTCAGTTCATTTGGCCTGACCACGAAAAACCA
TGCCTTGAACCCCCAGAACTTGCAATCCCTCCGATTGATTTTGGAAGCTTCCTCACTGGAGATCCTTTGG
CGGTCTCAAAACTTGCCCAGCAAGTAAATGAGGCATGCAAGAAGCATGGTTTCTTTGTAATTGTTAATCA
TGGTGTTGACTCAAAGCTTATAGCTAAAGCTCATGAGGGTATGAACATGTTCTTTGGTAAGCAGCTCTCG
GAGAAGCCAGTTCAAAGGAAGATAGGAGAGCAATATGGGTATGCCAGTAGCTTCACTGGAAGATTCTCCT
CCAAGCTTCCATGGAAAGAAACACTTTCTTTCAGATATTGTGCTGACAATCAGTCCTCTAACATCGTCCA
AGAATACTTCTTGAATGTAATGGGAGAAGAATTCAAACAGTTCGGGAAGGTGTACCAAGAATATTGTGAA
GCCATGAACACTCTTTCCCTTGGAATTATGGAGCTATTAGGAGTAAGTCTAGGAGTTGGAAACGAATATT
TTAGAGATTTCTTCGAAGGAAATGATTCAATAATGAGACTAAATTACTACCCTCCTTGCCAAAAACCGGA
ATTAACTCTTGGCACTGGGCCTCACTGTGATCCTACATCCTTAACAATTCTTCATCAAGATCATGTCAGT
GGCCTTCAAGTGTTCGTCGAAGAAAAATGGCACTCCGTTACTCCTGATCCGGAAGCTTTCGTCGTTAACA
TTGGTGACACATTCATGGCTCTATCAAATGGCATTTTCAAGAGTTGCTTGCATAGAGCAGTGGTAAATAA
CGTAACAGTAAGGAAATCCCTCGCTTTCTTTCTATGTCCAAAAATGGACAAGGTGGTGAAGCCACCAAAT
ACTTTGGTCGACTATAAAAATCCAAGGGTATATCCGGACTTCACATGGCTAGCTTTGTCAGAATTCACAC
AAAAACATTACAGGGCTGACATGAAAACCCTAGATGTCTTCACAACATGGCTTCAACAAAAAAACAACTG
CGGGGGTACCAAAAATGCTTCTTCCTGTTAATTAGGCTGAGGAAAGTGACATTATAACAGTGGACAATGC
TAGGTGCAAAGAAGGAGGGACTTGGTTCTTCATGGTGTGTATTTAGAGGAAAAAGGCAACAGAAGAACCA
AAAAACAGGCCAGCCCTGTAGTAGCTGCTAGTGTGTTAGTGTTGGAGAGAATCACATCAATGCAAGTAAG
AAGCAAATTGTAGGAATAAAGATTTGAATGGGCTCCATATATGCATGTCTTCTTATTGTTTTTTATTATT
ATTTTAATTTTGTTAATGCAAGACATGTATAGATTCTATGTACTTATGTGTTTGGACACCACCAAATTCC
GGTTGCCTCGA

>*PtrGA20ox1-B* LOC7466856

TCTATAAATTGCTTCTCTGAGACACCGGCCCTCATCAATGTCTCTCCTAATGGACTCAACCTCTTCAAGT
CTTCTCCTGTCTCCTCCTCCATTTCACAGCAAAGATGAAACTGATGCTCCTGTTCTTTACTCATCTTTCT
TGCAAAAACAAGCCAACATGCCCACAGAGTTCATTTGGCCCATTGGAGACTTAGTTCACAACCAAGATGA
GCTTAAAGAGCCATTGATAGACTTGGATGGATTCTTGAAAGGTGATGAGCGAGCAACTGCTGATGCGGCT
GAGCTTGTTAGGACTGCCTGCTTGAACCACGGCTTCTTTCAAGTCATTAACCATGGAGTTGATATAGGTC
TCATTCATGCTGCTCACGAAGAGATTGATAAAATTTTTAAGCTGCCTCTTGACAAGAAACTCAGTACTCG
TAGGAAGCCAGGCGATGTGAGTGGATATTCTGGTGCCCATGCACATCGATATTCATCCAAGTTGCCATGG
AAGGAGACATTTTCTTTTGGCTACCATGGGGATGATGATTCTGAGCCACTTGTTGTTGATTACTTCAAAT
CTGTCTTAGGAGAGAACTTTGAACACACAGGGTGGGTTTACCAAAGATATTGCGAAGCAATGAAGAAAGT
ATCTCTAGTTATTTTTGAGCTGCTGGGCATTAGCTTGGGAGTTGATCGCTTACACTATCGGAAATTCTTT
GAAGATGGAAGCTCAATAATGAGGTGCAACAATTATCCACCTTGCAATAATTCTAGCCTCACTCTTGGCA
CTGGTCCTCATTGTGACCCAACTTCCTTAACAATACTTCATCAAGACCAAGTTGGTGGTCTTGAAGTCTT
TGCAAACAACAAATGGCAAGCAATCCGACCTCGTCCAGATGCCCTAGTTGTAAACATTGGTGATACTTTC

ACGGCATTATCCAATGGGAGGTACCAGAGCTGCCTTCACAGAGCAGTGGTGAATAGGGAGAGAGAAAGGA
AATCATTGGTTTTCTTTGTGAGTCCAAAAGAGGAAAAGGTAGTCAGACCCCCACAAGATCTTGTTTGCAG
AGAAGGGCCGAGGAAGTACCCGGATTTCACTTGGTCAGATTTGTTGGAGTTCACGCAAAAACACTACAGA
GCTGATGTCGCTACACTCCAAAGCTTCATTCAATGGCTTTTATCTGCTAAACCATAAAGCATCAACCTTG
TAGTTGCTTTGGATGCTTTTGATGATTGGTTGCCTTTATGTATCCTGTGTACTAGCTAGGCTAGCTATGT
TCTCTCTATATTTTCTCAGGTTGGTACAATTTCCACCAGCTTTTGTGTAGCTTTCTGAATTTTATTGGAA
GAAAGAATAAAAACTAATACCTTGCTAGTGTTCTA

>*PtrGA20ox1-D* **LOC7466751**
AAAAAAACACCTTATAACTGATTTCGTTCAAGAGATCATGTCCATATAAAATTTATCCTTCTCTAACCCT
CCGTATCTCTCGTTTTCTACTGAAATCAATTCTATAAATAGCTTGTCTAAAAAACCAGCTCCTCATCAAT
GTCTCTCCTAATGGACTCAACTTCTTCAAGTCTTCTCTTGTGTCCTCCTCCGTATCTTACAAAAGATGAA
ACTGGGGCTCTTGTTTTTGACTCATCTTTCTTGCAAAAACAAGCCAGCTTGCCCACAGAGTTCATGTGGC
CACATGGAGACTTAGTTCACAACGAAGATGAGCTTAAAGAGCCAATGATAGACTTGGAAGGATTCCTTAA
AGGTGATGAGGTTGCAACTGCTCAAGCTGCTGAGCTTGTTAGGACTGCTTGCTTGAACCATGGCTTCTTT
CAAGTCACCAACCATGGTGTTGATATAAGTCTCATTCATAGTGCTCATGAAGAGATTGGCAAGATTTTCA
AACTGCCTCTTGACAAGAAACTCAGTGTCCGTAGAAAGCCAGGTGATGTGAGTGGATATTCTGGTGCCCA
TGCACATCGATATTCATCCAAGTTGCCATGGAAGGAGACATTTTCTTTTGGCTACCACGGGGATGATGAT
TCTGTGCCACTTGTTGTTGACTACTTCAAGTCCGTCTTAGGAAAAGATTTTGAACACACTGGGTGGGTTT
ACCAAAGTTACTGTGAAGCAATGAAGAAAGTATCCCTAGTGATATTTGAGCTGTTGGCCATCAGCCTAGG
AGTTGATCGTTTACACTATCGCAAATTCTTTGAAGATGGAAGCTCAATAATGAGGTGTAACTATTATCCA
CCTTGCAATAATTCTACCCTCACTCTTGGCACTGGTCCTCACTGTGACCCAACTTCCTTAACAATACTTC
ACCAAGACCAAGTTGGTGGTCTTCAAGTCTTTTCAAACAACAAATGGCTAGCAATCCGACCTCGTCCAGA
TGCCCTAGTTGTAAACATTGGTGATACCTTCATGGCACTATCCAATGGGAGATACAAAAGTTGCCTTCAT
AGAGCAGTGGTGAACAGGGACAGCGAGAGAAGATCACTGGTTTTCTTTGTGAGTCCAAAAGAGGAAAAAG
TAGTGAGACCCCCACAAGATCTTGTTTCCAGAGAAGGGCAAAGGATTTACCCGGATTTCACATGGTCGGA
TTTGTTGGAGTTCACGCAAAAGCACTACAGAGCTGATGTCGCTACACTCCAAAGCTTCATTCAATGGCTT
TCATCTTCTAAACCATCTACCTTTTAGTTGCTTTGGATACTTTTGATGATTGTATGCCTCTGTGTATCAT
CTAATTTAGCTAGGCTAGCAGCTATCTCCTTTGTATATTTTCTCAAATGGGTGCCTGGGTTGCCACCACC
TTCTCTGTAGCTTTCTGAATTCACTTGCAAGAAAAATTGAGCGCCCGTGTTCTGCCTATAGCGCATTCAA
AGAACCAAAAAACTATTCTAGTA

>*PtrGA20ox2-1* **LOC7482127**
TGGAATATCACCTAATTTTCTCATTGAGTAACTCCTTAAACTGCAAATCCTCCCCTCCCCTGCAAAAATA
AAAATCCACACAAGGCATATAAGAGTGAGGGAAAGACAGGTAGCTACAAGATTCCAATATTTGGGCTCGG
CGATTTCCCCAATGAGAGCGGTCTTGGCTTTTCTTTTATCATCATCACACTTCTCTTCCGTTTCTCTTGA
CTGGCTAGCAGCTACTCTCCCTGCCACTTACATATATATCTTGCCTTGCCATTTCTTCGAAGCTAGCTAT
CACACTAGTCACAAATTCGTAATGCAATGGCAATAGATTGCATCAAAACCATGCCTTCAAAAACCACCCA
TCATCACCATCCAAAAGATCAAGATCAATGCAAAGATGATAACAAGTCTTTAGTGTTTGATGCACAAGTT
CTTAGATACCAATCAAGCATACCTCAACAGTTCATTTGGCCTGACCATGAAAAGCCTAGTGCTAATGCAC
CTAAACTCCAAGTTCCACTTATAGACTTGGGTGATTTCCTCTCTGGTAACCCTGATGCTGCAATGGAAGC
TTCAAGACTTGTTGGTGAAGCATGTCAAAAACATGGTTTCTTTCTTGTTGTTAATCATGGAGTTGATAAG
ACACTCATTGCTCATGCTCTTAATTACATGGACAACTTCTTTGAATTGCCTCTTTCTGAGAAACAAAAGG
CTCAAAGAAAAATTGGCGAGTCTTGTGGATATGCTAGTAGCTTTACTGGCAGGTTTTCCTCCAAACTTCC

ATGGAAAGAAACACTTTCTTTTAGCTATACAGCTGAGAAGAATTCATCAAAACATATTGAGCAATATTTT
CATGACAGAATGGGGGAAGACTTCGCCAAATTCGGAAGGGTGTACCAGGATTACTGCGAGGCCATGAGCA
CTTTGTCACTAGGGATCATGGAGCTATTAGGAATGAGCCTTGGTGTGAGCAGAGCACATTTCAGAGAATA
TTTCGAAGAGAATGATTCAATAATGAGGCTTAACTACTACCCTCCATGCCAAAAGCCTGAACTTACTTTA
GGAACTGGTCCTCATTGTGATCCAACTTCTTTAACCATCCTTCATCAAGACCAAGTGGGTGGTCTTCAAG
TGTATGTAGACAACGAATGGTGTTCGATTAGCCCCAATTTCGACGCTTTTGTCGTTAACATTGGCGACAC
CTTCATGGCTCTATCAAATGGTAGATACAAGAGTTGTTTGCACCGAGCGGTGGTGAACAGCCAAACGCCA
AGAAAATCTCTTGCTTTCTTCTTGTGTCCAAGGAATGACAAGAAGGTAACTCCACCAAAGGAATTAGTGG
ACACATGCAATCCAAGAATATATCCAGATTTCACATGGCCTATGTTGCTTGAATTCACACAAAAGCATTA
CAGAGCTGACATGAAGACACTTGAGATGTTCACAAATTGGCTTCAACAAAGAAATGTTAGCTGAACAAGT
GGGTCAAGTTATGTGTCGCATAAATAGACCTGGTGGCATGCTATAGGCCTCTTTATCAAGGACAAAAAAA
GGAAAGTGATACTGATGGGGAGGGGAGTGGAAAGGGAAAGAAATGAAGCTTGAAAGAGATGGGGATTTTG
ATGAGATTACACTACATGAAGAGAGGAAAAAAAATTTGAGACTCTCTCAAAGGACAACTTTCCTTTATGC
CCATAGGTCTGGTAATTACTAGCTAGCTTATTTATATTTTATTGGCCATGAACAAAACGTAAAATCCACA
GACCAGTAAAATGTTGGATTGGCTAATTGCTTTTTGTATTTTCTTTGATAATGTAAAAAGGTTATCACTA
GCATCTA

>*PtrGA20ox2-2* LOC7469184

GCCACCCAATATTCACCTTTGTTTGTCTTTCTCAATGGATTCTCCCCTAAAATTACAAGAGCAAAGCAAG
GGTTTTTTATTTGACTCAGTGTTGCACAAACAAGCTGGTTTTCCCAAAGAATTTCTTTGGCCAGACTTGG
TTAGGGCTCAACAAGAGCTCTCGGAGCCACTGGTGGACCTGGAGGGGTTCTTCAAAGGGGATGAGGAGGC
AACAAAACAGGCTGCTAATATAATCAAGGATGCATGCTCGAGGCATGGTTTCTTTCAAGTGATCAATCAT
GGAGTAGATCCCAATGTTATTAGAGACGCAGAGGATTACATGGACCATTTCTTCAGGCTTCCAGTTTCCG
AGAAACTTAAGGCTCGGAGGATGCCAGGCAGCTTGTGTGGTTATTCTGGTGCTCATGCTGATCGGTATGC
ATCAAAACTTCCATGGAAAGAGACACTCTCTTTTCGTTACCATGAAAATTCTTCGGATCTCGTTGTGCTA
GATTTCTTCAAATCTGCTCTTGGGAATGATTTTGAGCAAACAGGAATGGTATATCAAAAATATTGTGAAG
CTATGATGGATTTATCCTTCGCCATATTGGAACTACTGGCAATCAGCCTGGGAGTTGATCGAAAACTTTA
CAGAAAATTCTTTGAAGATGGTTTCTCGATATTGAGATGTAACTTCTACCCTCCTTGTCAAGAGCCTGGA
AATACTCTTGGCACGGGACCCCATTGCGATTCAAATTCAATAACCATACTTCACCAAGATCAGGTTGGAG
GCTTAGAAATTTTCACAAACAAAGTATGGCAGACAATTCCACCCCTTCAAGGTGCACTCATCATCAATAT
TGGTGATACCTTCACGGCATTATCCAATGGGAAATACAAGAGTTGCTTGCATCGGGCGATGGTTAACCAG
CATGAGCAGAGGAAATCTTTGGCATTCTTTCTCAGTCCAAGAGAAGACAAGGTGGTGAGACCACCACAAG
AGCTTGTGTGCAGTGAAGGGAAAAGGATGTATCCAGACTTCACATGGTTGAATTTGTCTCGATTCGTACA
AAATCACTATAGAGCTGATGATAGCACACTTCAAAACTTCACCAACTGGTCCCAATCAGTAAATCTCTAG
TGGTCTTGCTAGCTCCTTCATGTTGTCAGGGACTCTTTTCTCCCCCGTATCGTATGCCTAATAAGTTCAA
AGTCGGGTTTTATTTTTCTGATTAGGTTATTCTAGTTTCTACATATCCTGACCAATATGGTCACAGATCA
TAAGCATGCTTTGGCATTCTGTATTGTTTTTCTCATGATTGCTATTTCCTAAGTACCTTCTTTGATCC

>*PtrGA20ox2-3* LOC7460983

TTGCGGTATAGAATATTGGTTTACATGAATCTGGTCCATGAAATAATTTTTTCTATAAATTCCATGTCCT
TGCCTATTAATTCTTGCTTGTTACCTGTTTTAAGAATTATTCGAGTGCCTTTGCACTTCTTTTGCCAGCC
CACTTCTAATTCCAAGGTTAGAGATCGAGGGAGGTTTTTATTTATTTCCTTTTGAAAAGACAAACTCGAT
CAGAATGCCAAGAAGATTTGTTTGGCCAAAACAAGACTTAGTTGGTGCTCATCAAGAGCTCACGGAGCCA
GTCGTGGACCTTGAGGGTTTCTTTAGAGGTGACGAGGAGGCAATAAAGCAGGCTTCTGATATCATTAAGG

CTGCTTGCTTGCAACATGGTTTTTTCCAAGTGATCAATCATGGAGTTGATCTCAATCTCATTAGTCTCGC
CCATGATCACATGCATAATTTCTTTAAGCTTCCAACTTGCGATAAACTAAGGGTTCGGAGGATGCCAGGT
AGCATTTGGGGGTATTCTAGTGGACATGCTGATCGGTATTTGTCTAAATTGCCATGGAAAGAGACGTTAT
CTTTTGGCTACAATGAAAACTGTCCGAATCCTATCGGGATAGACTTTTTCAAGTCTACCTTAGGGAAGGA
TTTTGAACAAACAGGCTTGGTCTATCTAAAATATTGTGAGGCTATGAAGGGTTTGTCCCTCTCCATAATG
GAACTATTGGCAATCAGCTTGGGAGTTGATCGAGGGTATTACAAAAGTTTTTTGAAGATGGTTGCTCTA
TAATGAGATGTAACTTCTACCCTCCTTGTCAAGAGCCAGGTCTTATTCTAGGCACTGGACCTCATCGTGA
TCCCACATCGCTAACCATACTTCACCAAGATCAAGTCGGAGGTCTAGAAGTTTTTCAGACAACGTATGG
CAGACGGTTCGTCCCCGTCAAAGTGCCCTTGTTATTAACATTGGCGATACCTTTATGGCCTTATCGAATG
GGACCTACAAGAGCTGCCTGCACAGGGCTGTGGTCAACAAGTACGAGGAGAGGAGATCGTTGGCATTCTT
TCTTTGCCCAAAAGAAGACAAGGTGGTGAGACCACCACTGGATCTTGTGTGCAATCAAGGGAGAAGGATG
TACCCAGACTTTACATGGTCGGATTTGCTCCATTTCACACAGAAGTACTACAGAGCTGATGATGCTACCC
TTCAGAACTTCACCAAATGGATCATTTCTCTAAAATCAGCAAGCCTCTAATGCTCTTATCATGGGATCTT
TACTCACGTCAAGTACATATATACATGTCCGATGTTCGTGCCTAGATGTCAATTAAGGTTTCCATAGGCC
TTGATTGTCAAAATCATGAAGATATATATGTGCTTTTCTTTTCTGTGTGGTTGTGCTCTGTATCGCTGCT
CTTCTTATTTTGTTATGTTTTGCACCCTTTTCTTCTGTAACGTTATCTGTCTTTCCTTCTTTCATTCTCT
TGTGTTTAGGAAAGCACTTAGATCGATTCTTTTTGGTCATATCGTTTGTTCATAAGTAAAGCATTTTTTT
AGTTGTTAACTA

>*PtrGA2ox*   LOC7490835

CTTTCCCTCGGTAGCCTAAATGCCAAGCAGACACACAAAGCCAGATGGCTGCTCATTATAGATAGTAACG
TTTCTGTCCCTCCTCCCCCCCATCTCACACGCACCCCCTCTCTCTCTCTCTGTCATGCATTAGCTTCCTC
GCCCATTCGCCCATTTATAGCCATCAGTCTCACCAGCCTCTCTAGTACCTCACCAAGCCAAATTCTTCTC
TTAATTCTGCAGTCTCTTATCTTGCTTGTCTTTTTAGTGTGGAGAAACTAATAACAAGCCATGGTAGTAC
TTTCCCAACTAGCTCTAGAACCTTTCTCAGTTATCAAAACATGCAAGCCTATAGGCTTGTTTTCCGAGAT
ACCCGTTATAGACTTGACAGACCCTCATGCCAAGACTCTCATAATCAAGGCCTGTGAAGAGTTTGGATTC
TTCAAGCTGGTAAATCATGGAGTTCCAATGGAAGTCATGACCAAATTGGAAGCTCTTGCTACCAACTTCT
TTAACCTCCCCCAGCCTGAGAAAGACAAGGCTGGACCTCCTAACCCTTTTGGCTACGGAAACAAGAAAAT
TGGCCCTAATGGTGATGTTGGTTGGGTTGAATATCTCCTCCTTAACACCAACCCTCAAATCTCCTCCCAA
AAAACGTCCATTTTTCAGGAAAACCCACAGATTTTCCGCTCTGCTGTTGAAGATTACATACTGGCAGTGA
AGAGAATGGCTTTTGAAGTATTGGAATTGATGGCTGATGGGCTGGAGATTGAGTCAAGGAATGTGTTCAG
TAGGCTGTTGAGGGATGATAAGAGTGATTCATGTTTCAGGCTAAACCACTACCCACCATGCTCAGAGCTG
CAAGCATTGAGTGGTGGAAATTTGATTGGGTTTGGCGAGCACACAGACCCACAGATAATATCTGTTCTAA
GATCTAACAACACAAGTGGCCTGCAAATTTGTCTGAAAGAGGGAACTTGGGTTTCAGTCCCCCCTGATCA
GACCTCTTTTTTCATCAATGTTGGTGATGCCTTGCAGGTGATGACAAATGGAAGGTTTAGGAGTGTCAAG
CACAGGGTTTTGGCTGATCCCCTGAAACCAAGGATTTCCATGATTTTCTTCGGTGGTCCACCTTTAAGTG
AAAAGATAGCACCTTTACCCTCCTTGATGGCAGAAAGAGGAGGAAGCTTATACAAGGAGTTCACATGGTT
TGAATACAAAAGGTCCGCATACAAGTCAAGGCTAGCTGATTACAGACTTGGGCTATTTGAGAAAACTGCA
GGCCAATGATGTGGTCAATTCGTATTTTAGTAAAAATTAGGGTTTCGCGAAAACTCAAAATTAATAGGTG
TGAAGACTTTACCCAACTAGCCACATGCAGATGGAAAGCAAATAGGCTCTTCGTTTACTCTTCTTCATGA
CTTCTTACCCCCCCCCCCCCCCCCTCTTTTTTTAACTTATTTCTCGTTTAAATGTATCGACAATCACTCT
TCTATCATTAATGCATTTTATCATCATGTCATGATGTATAAAGTTATGCCAACTTTTCTCAGATTCCACT
TCAGAACATTTCTTTTTATCCAAAACTATAAGATTTTACCAGCTGGGGGCTTCCCCACTTTTCTGGTCAG
ATTGCCCATTGTTATTACTTTCCACCATTATCTTAAAAAATATCCCCATCACATCAAACCATGAATAAGA
AGAGTTTTGGGAA

>*PtrGA2ox1-1* LOC7471887

GTGTATATTATAAGTAGCATTTTTGTATATCAACTCAGCCGAGCTCTCTCTATCTCTCTGTCTTCTTTCC
TCCTGCAGACAACAACAACCATGGTGCTCGTGCCCAAGCCAGCACTTCAACAGTTCTCGTTTATAAGAAA
CATCAAACCCACCACATTCTTTTCTGGAATCCCTCTAATAGACCTCTCAAAACCCGACTCCAAGCACCTC
CTGGTCAAGGCATGTGAGGAATTTGGATTTTTCAAGGTAGTCAACCATGGCGTGCCTTTGGAATTCATTT
CGAAGCTGGAATCGGAAGCTGTCAAGTTCTTCTCATTACCGCTTTCTGAGAAAGAAAAAGCAAGCCCTCC
TAATCCCTTTGGCTATGGTAAGAAAAGCATTGGACAAAACGGTGATGTGGGTTGGGTAGAATACCTTCTC
CTGACCACTAATCAAGAATCAGTTTCTCAGAGACTCTCTTCAGTTTTTGGTGACAACCCAGAAAAGTTTC
GGTGTGCTTTGAATGATTATGTATCAGCTGTGAAGAAAATGGCATGTGAGATTCTTGAAATGATGGCTGA
TGGTCTAAAGATTCAGAATAGAAATGTGTTTAGTAAACTTTTGATGGATGAACAGAGTGACTCTGTTTTT
AGGCTAAATCACTACCCTCCATGCCCAGAGATTCAAGCATTGAAAGACCACAACATGATTGGATTTGGAG
AGCACACAGACCCACAGATCATTTCTGTGTTAAGATCCAACAACACTTCTGGCCTGCAAATCTCTCTTAA
TGATGGAAGCTGGATTTCAGTCCCACCTGACCCGAGCTCATTTTTCATCAATGTTGGCGACTCATTACAG
GTTATGACTAATGGGCGGTTTAAAAGTGTAAGGCACAGAGTCTTGGCCAACAGTATAAAGGCTAGAATTT
CAATGATTTATTTTGGTGGACCACCTTTGAGAGAGAAAATAGCTCCATTGCCCTCGCTCATGGAAGGAGA
AGAAAGCTTGTACAGAGAATTTACATGGTTCGAGTACAAGAGATCTGCTTACAACTCAAGATTGGCCGAT
AATAAGCTTGTCCTCTTCGAGAGGATAACAGCCTCGTAAATAAATACGTACACAAAATCAAGTCTCGCGT
GTTCTTGCCAAATACTTCAAGAATAGGTTTAGAAAGGCTTACTGTATTTGCCATAGATGGAATTAATACT
TCGTATTTTCTACTCTGACGTTCTTCTTCTTTGACTTTCTCCAGTTTTTGTCTCCCATACTTTATCCAAT
TACTTTTCTTGTGAA

>*PtrGA2ox1-2* LOC7459116

AAATCCACTTCTATAAATAGCCAAGAAAACCTAAAAGGCAAATGCCCATCTGTCTCTCAGTACAAAGCTA
AGACTTAACAAATTTCATTCCATCAACTCAACCTAGACCTCTCTCTCTCTCTCTCTCCTGCTTCCACACA
ACAACAACAACAATGGTGCTTATATCCAAGCCAGCTCTTGAACAGTTCTCGTTTATAAGAAACAGAAAAC
CTACCACAGTTTTTTCTGGAATCCCTCTAATAGACCTCTCAAAACCTGACTCCAAGCACCTACTGGTTGA
GGCATGCGAGGAATTTGGATTTTTCAAGGTAATCAACCATGGAGTGCCTATGGAATTCATATCCAAGCTG
GAATCTGAAGCTGTCAATTTCTTCTCATTACCGCTCTCTGAGAAAGAAAAAGTAGGCCCTCCTAGCCCCT
TTGGCTATGGTAACAAAAGCATTGGACAAAATGGTGATGTGGGCTGGGTAGAATACCTTCTCTTAACCAC
TAATCAAGAATCAATTTCTCAGAGGTTCTCTTCAGTTTTTGGTGACAACCCAGAAAAGTTTCGGTGTGCT
TTAAATGATTATGTATCAGCTGTGAAGAAAATGGCATGTGAGATTCTTGAAATGATGGCTGATGGTCTAA
AACTTCAACAAAGAAATGTGTTTAGTAAACTTTTGATGGATGAACAGAGTGACTCTGTTTTTAGGCTAAA
TCACTACCCTCCATGCCCAGAAATTGAAGCATTGACTGACCAAAACATGATTGGATTTGGAGAGCATACA
GACCCACAAATCATTTCTGTGTTAAGATCCAACAACACTTCTGGCCTGCAAATCTCTCTCAGTGATGGCA
GCTGGATTTCAGTTCCACCTGATCAGAACTCATTTTTTGTCAATGTTGGTGACTCATTACAGGTTATGAC
TAATGGGAGGTTTAAGAGTGTCAGGCACAGGGTTTTGACCAACAGCATGAAAGCTAGAGTTTCAATGATT
TATTTTGGTGGACCACCTTTGAGTGAGAAAATAGCTCCATTGCCCTCGCTCATAAAAGGAAAGGAAAGCT
TGTACAAAGAATTTACATGGTTCGAGTACAAAAGATCTGCCTACAGCTCCAGATTGGCTGATAATAGGCT
TGTCCTCTTTGAGAGAATAGCAGCCTCGTAAGCACACAAAATCAAGTCTCGCATGTTTGGTAAATGGTCT
TGTCAAATACGTCAAAAAAAAAAAAAAAAGGTTTAGAAAGGCTTATTAGACGTATAAGGAATCAATACTTT
GTATTTTCTGTTTTTACTTTGTTCTTCCTCGACTCTCTCCAATTTTTGTTTTCCACACTTCATGTAATCA
CTTTTCTTGTTAATAAAACTACTTTAGCTTCA

>*PtrGA2ox2-1* LOC7495200

CCCCGTCACCTCCTCTCTCTCTATCTCTCTATGTTGCAGTATATAATAGGGTAGCACCCCGACCCTTATG
GATCATACCTCCAAAATTCAAAGTTCAAACTAAAATTCTTGATTTTTACCTTTGATAATTCCCTCCCTCC
CTCCCTCCCTCCCTTAATTATAAGCAAGAATGGTAGTTCCTTCTCCAACACCAATACGAACCAAAACGAC
GAAGGCTTTAGGAATTCCTACTGTTGATCTTTCTCTTGACAACTCGAGCGTATCACAGTTGATCGTAAGA
GCATGCGAAGAATATGGATTCTTCAAAGTTATAAATCATGGGGTCAACAAGGAGGTCGTGACAAGACTGG
AGGAGGAAGCAGCTCGTTTTTTCGGAAAACCAGCCGCGGAGAAGCAACAGGCTGGACCTGCTAGTCCTTT
TGGATATGGTTGCAAGAACATTGGCTGCCATGGTGACACGGGCGAGCTCGAGTATCTTCTCCTCCACACC
AATCTTCTCTCCGTCTCTGAAAGATCCAAAACCATCTCGAACGACCCTTCAGGATTCAGTTGTGCGGTGA
GTGATTACATAAGAGCAGTTAGGCAGCTTGCATGTGAGATTCTTGATCTTGCGGCTGAGGGCTTGTGGGT
CCCGGATAAACATGTATTCAGTAGGCTCATCAGAGACGTCCATAGCGATTCAGTTCTTAGACTTAATCAC
TATCCTGCAGTCGAAGAGATCGCAGATTGGGACCCATCACCTATAAGAATTGGTTTTGGTGAGCACTCCG
ACCCTCAGATCTTGACCATCTTGCGATCCAACGATGTTGCGGGCCTCCAAATTTGCTTGCATGATGGTTT
GTGGGTACCCGTCCCTCCTGACTCCACAGGATTCTACGTGATTGTGGGTGATTCCTTTCAGGTTTTAACC
AATGGGAGATTTGAAAGCGTGAGGCATAGGGTATTGACAAACTCGAGCCAGCCAAGAATGTCAATGATGT
ATTTTGGGGCACCACCCCTTACTGCATGGATCGCTCCTCTCTCACACATGGTTTCACAGCAAAATCCAAG
CCTTTATAAACCCTTCACTTGGAGTGAATTCAAGAAAGCTGCCTACTCTCTACGTTTGAGAGATACGCGT
CTTGACCTCTTCAAGATTCACGCCACTGAAAAATCTGCTTCATTGTAATTGGCCTTCAGTATATTATTTA
AGGTCCCAGTCTTGTACTTCTTGGATAGGATAGAAAGAATTAGGGACGGAAAAAGAAAAAGAAAAAGAAA
ACTCTTGCAGATGTAAATAGGTCATAAAGTTAAAGTTGACGGATTATTCTATTGGATCGGCTTCTTTCTC
TCCTTTCTTAATTTCTAGCACTCTGTAACATTTGTTTTTGTTGTAGCATCTCGTGAATGTAGGGAAAATA
GTGTAAGTCAGGTGCACGTCACTCTATTAAAAAAAATATATGGCTGTTTATTTGTATGCTTTTTGTGCA
CAGCCTTGTACTTCACAGTTATAATAATGTTAGATTTTCAATACTA

>*PtrGA2ox2-2* LOC7471090

AGTCAACTTTTCTTACATTAAACAAGATTTTTCTAGTTGTACGTCAACCACCCAAAAAAGCATTAGATTA
TTGAGTGCCCAAGAGATATAAGGAAACAAATTAATATTTGAAAAAATGAAGAAGAAATTAATGATTTTAT
CAATAATCTGCATCCTCTTTGAATGAGACTCACTCTCCTATATATCTATAGCAAGTATAAGCAATACTCT
TCACCCCTCTCTCACATTCTCACCTTCTTCTTCCTAGTCTCTCCATCTCTCAAATATACCGTCATCTCTT
CTTCTCTTCTATATCTTCCAAGTGCTATACTCTTCCTCTTGATTTCCTTTCTCCTGTATCCCCAGAGATA
TATAATTATAGAGATATGGTAGTGGCATCTCCAACTAAACTTCACAGTGAAGAACACCTGGCCATTGAGC
TTCCTACAGTAGACCTCTCAGGAGATAGATCAATGGTTTCCAATCTCATTGTCAAAGCCTGTGAGGAATA
TGGTTTCTTCAAGGTGAAAAACCATGGTGTTCCTCATGACATCATTGCCCAAATGGAGAAAGAAAGTTTC
AACTTCTTTGCCAAACCATTCGATGAAAAGCAAAAGGTTGAGCCGGCCAAACCCTTTGGTTATGGCTGCA
AAAATATTGGCTTCAATGGTGATATGGGAGAGGTGGAATATCTACTTCTTAATATCAACCCTCTCTCCAT
AGCTGAAAGGTCCAAAACCATCTCCAATGACCCTGCAAAATTCAGCTCCGCTGTGAGTGCTTACATAGAA
GCAGTTAGGGAGCTGGCATGTGAGTTATTAGATCTGATGGCGGAGGGGCTGCGGGTCCCGGATAGATCAG
TGTTTAGTAGGCTAATCAGAGATGTTGACAGTGACTCCCTCATCAGGTTAAACCACTACCCCCCTATGCC
TCTGCTCTGCAAGGATGAGGACTCGTCACCTTGTAACCAAAACAAGGTAGGGTTCGGAGAGCATTCTGAC
CCTCAGATCTTGACCATCTTGAGATCCAACGATGTGGGTGGCCTTCAGATTTCCTTAAATGATGGTGCGT
GGGTCCCAGTAACACCTGATCCCGCTACCTTCTGGGTTAATGTGGGTGACTTGTTACAGGCAATGACAAA
TGGGAGATTTGTAAGTGTGAGGCACAAAGCCCTAACCAACTCTTCCAAGTCTAGAATGTCAATGGCCTAT
TTTGCTGGCCCTCCACCCAATGCGAGGATCACTGTTCCACCAGAAATGATTACCCCCACCAAGCCGGCAT
TATACAAGCCCTTTACCTGGGCTGAATTCAAGAAAGCTGCTTATGCTATGCGACTTGGAGATAGACGCCT

TGGTCTATTCAGGATGGAAGGAGACGAGCAAGTTGCTTGATAAGTTTTTGTTTTTTGATAGTCGAGTATT
ATGTAGTAAATATGTAAGTTTAGTGTCTCTTTCTGTACGCCTGCCCTAGAAAGAATTTTGCTGTTGGATA
TCCTTTTCATTGCCCCTGCTTATAACAGGATATCCAGAGACGGCGTTGTAGTACGGGGGATATGAGTAAA
TGGTGCGGTAGTATCACCTTAGTCTAGTCATCAGTGTAAAAAACTTTCTTCTGAATCCTGTCTAGTGGCC
AGTAGGTGGTGGTTAGCTCTATCATGGATTCTTTCCACATGACGAGCAGATCGATAGTGAAAGCCCTACC
AGGATAAGATGTTACATGAAGGTGAAATAAATTTGAATATGGAAAATCAA

>*PtrGA2ox2-3*   LOC7485848

TCTCCTATATATCTATAGCAACTTAGCAAGCATGAGCAGGCACCTCACCGTTTCTCACCTCCTTCTTTCC
AGTCTCTCCATCTCTCAAAATATTCCTCTGTCACCTCTTCTCTTCTATATCTTCCAAGTGCTCCTCTTCC
TTGTTTTAGTTTCCTTTATCTTGTGTCTCCGAAGATATATAATTATAGACATGGTAGTGGCATCCCCAAC
TCAAATTCACGGGGAAAAACTCCTGGCCATTGAGCTTCCTGTAATAGATCTCTCAGGCGAGAGATCAATG
GTGTCCAGTCTCATTGTCAAAGCCTGTGAAGAATATGGTTTTTTCAAGGTAAAAAACCATGGCGTTCCTC
ATGACATCATTGCCAAAATGGAAAACGAAAGTTTCAACTTCTTTGCCAAAACATTCGATGAAAAGCAAAA
GGCTGGGCTTGATAATTCCTTTGGTTATGGCTGCAAAAACATTGGCTTTAATGGTGATACAGGAGAGGTT
GAATATCTGCTTTGTAATACCAATCCTCTCTCCATTGCTGAAAGATCCAAAACCATCTCCAATGACCCTA
CAGAATTCAGCTCTGCAATGAGCGGCTACATAGAAGCAGTTAGGGAGCTGGCATGTGAGTTATTAGATCT
GATGGCAGAGGGATTGTGGGTCCCAGATAGATCAGTGTTCAGTCGACTAATCAGAGATGATGACAGTGAC
TCAATCATCAGGTTAAATCACTACCCTGCCATGCCTATACTCTGCAAGGATAAGGACTCCTCATCACCTT
GTAACCACAACAAGGTAGGATTTGGAGAGCATTCTGACCCTCAGATCTTGACCATCTTAAGATCCAACGA
CGTGGGTGGCCTTCAGATTTCCCTGAATGATGGTGCCTGGGTTCCAGTAACACCTGACCCCACTGCCTTC
TGTGTTAATGTGGGTGACTTGTTACAGGCAATGACAAATGGGAGATTTGTAAGTGTGAGGCACAAAGCCC
TAACCAACTCATACAAGTCTAGAATGTCAATGGCCTATTTTGCTGCACCCCCACTTAATGCAAGGATCGC
TGTTCCACCAGAAATGGTTACACCCATCAAGCCTGCCTTATACAGGCCTTTCTCCTGGGCTGAATTCAAG
AATGCTGCTTTTGCTCTGCGACTTGGAGATAGCCGCCTTGGTCTATTCATGCTGGAAGTAGATGACCAAG
TTGCTTGATGAGTTTTTGATAGTTTAGTATTATGGAATATAAATGTTTAAATATCTAAGTCTAGCGTCTT
TCTGTACCCCTGAAGGGGAATTTTGCCGTTGGATATCCTTGCCATTGCTACTGTTTATCCAGCGAGAGAC
TAGTAGCTTGGGGGGCTAGGGGGGGCGAGAGTAAATGGTATGTGACACTGTAGTTTAGCCGTTAGTGTAA
AATTTTCCCTTTTGAATAATATTTAGTGGCCAGTGGGGTGGTGGTTAGCTCTA

>*PtrGA2ox2-4*   LOC7460479

ATTAACCGAAAGTGTCAACCTAGTCTTAGATTATAATATTAATTACCTCTCCCTCGCTCTCCGCCACTCC
TCTCTTATGTATAAGAGGTAGCGCCCTCGACCATTATCGATCATACCTTCGAGCTTCAAATTCTGGATTG
ATAGTTTTCTTCCTTCCTTCCTTCCTTCCTTCCCTAATTATAAGCAGGAATGGTAGTTCCTTCTCCAACT
CCAGCACGAACCAAAAAAACGAAGGCCTTTGGAATTCCTACTGTTGATCTCTCTCTTGATAGGTCAAACG
TATCGAAACTGATCGTAAGGGCATGTGAAGAATATGGATTCTTCAAAGTTACGAATCATGGGGTCAGTAA
GGAGGTCGTAACCAGAATGGAGGAGGAAGCTGCTCATTTTTTCTCAAAACCAGCCACGGAAAAGCACCGA
GCTGGACCTGCTAGTCCTTTTGGCTATGGTTGCAAAAATATTGGCTGCAATGGTGACATGGGCGAGCTTG
AATATCTTCTCCTCCACGCCAATCCTCTCTCCGTCTTTGAAAGATCCAAAACCATCTCAAATGACCCTTC
AGAATTCAGCTGCGTGGTGAATGATTACATACAAGCGGTTAAGCAACTGGCGTGTGAGATTCTTGATCTA
ACGGCTGAGGGCTTGTGGGTCCCGGATAAACGTGCATTCAGCAGGCTCATCAGAGATGTCCACAGCGATT
CTGTTCTTAGACTTAATCACTACCCTGCATTCGAAGAGATCATGGACTGGGACCCATCACCTAAAACAAT
TGGTTTTGGCGAGCACTCCGACCCTCAAATCTTGACCATCTTGCGATCCAACGACGTTGGGGGCCTCCAA
ATTTATTTGCGTGATGGCTTGTGGGTCCCTGTTCCTCCTGACCCCACCGGATTCTATGTGATCGTGGGGG

ATGCCTTCCAGGTTTTAACGAATGGGAGGTTTGAAAGCGTTAGACACAGAGTGTTGGCGAGCTCGGGGAA
GCCAAGAATGTCAATGATGTACTTTGGGGCGCCACCGCTTAACGCATGGATCTCTCCGCCTCCACAGCTG
GTCTTGCCACAAAATCCAAGCCTCTACAAACCCTTCACTTGGAGTGAATTCAAGAAAGCTGCCTATTCTC
TGCGATTGAGAGATACGCGTCTTGACCTCTTCAAGATTCATGCCACAGAAAAATTTGCTTCTTGATTTAA
TCTACAAGATTTATATTAAATGTAATTGGCCTTCCGTATATAAGGGTTCCTTTCATTATCGTACTTGTTG
GAGGGGATAGAAAGCATTAAGAGACAAAGAAAAGAAAGAAAAAAAGAAAAAAAAGAAGCTTTTGCTGCTG
TAAATAGGTCCGAACGGAAGTCGAAGGATTATTCTACATATCCGGAAAGGCAGAGGGAGAGTTTTCTCTT
TTTTCTTCTTTTTTACTTTCTTTCTCAAAGCACTTTGGTTCCTAGCATTTATTTAGTCGTACCGTATTGT
GAATTTTTATACGTATTCTCCCTCTTGTTTTCTTTTAA

>*PtrGA2ox8-1*   LOC7479098
CGTGATTTCCTCTCAAGCAAATACTCCTTACCCTTATATATTATCTTCTTCCTGCAGTACTGTGCCGATC
ATAATCATTGATGGTCTAGCTTTTCTTCTCTATAAAATATCTCCCCTCCAAGCTTAGTGGACGACACAAA
CGCCCTTTCTCTAGCTCCCTCCCTCCCTCTCTCATTCTCTTTGCGTCTCTGTTTTAGACCAGAAAAGGTT
TAATTCAAGTGCATATCAAGGAAAGCTTTAATGGTGGAATCTAGCAACTTTTTGCAGATTATAAACCAGG
CTCAAAGGATGGACGTTGACCCACCTTTCCAACTGACCTACAAGACCCTCTTGGAAAAAACCACAGAAGG
GGCAACTGATCATAAAGATGTTGTTGATATCATTGAGGAATGCGAGCTTCCATTGATCGATCTTGGCCGT
CTGAATCTTAAGAACTTGGAGAAAGAAAAGTGCAAGTCAGAGATTGCTAGGGCATCACGGGAGTGGGGGT
TCTTTCAAGTTGTAAATCATGGGATTTCGCGTGAGATTTTGGACAAAATGAGAAGTGAACAAGTTAAGGT
TTTTAAACAACCTTTTAATGAAAAGAGCAAAGAAGAAAAGTTTCTGAATTTCTCTAGGGGGACTTATCGA
TGGGGGACACCTACAGCTACTTGCCTTAAGCAGTTGTCATGGTCCGAAGCTTTTCATATTCCTATGAGTG
AGATACAGGTTTCCAATGGTTTCAGTACTGCTCTCAGCTCGACAATGGAACAATTTGCTACAACAGTTGC
CAATCTAGCACAGAAATTAGCTGAGATTTTGGCTGAGAAATTCGGCTGCAAATCTGATTTCATTAAAGAA
AATTGTCTGTCAAGCACTTGTTATCTACGAATGAACCGATATCCACCATGTCCAATCCCTTCAGAGGTAT
TCGGACTCATGCCACACACTGATAGCGACTTTCTCACCATTTTGCACCAAGATGAAGTTGGAGGATTGCA
GTTAGTGAAAGATGGGAAATGGTTTGCTGTCAAGCCAAACCCAGAGGCACTGATAATCAATATCGGAGAC
TTGTTTCAGGCTTGGAGCAACGATGTTTATAAGAGTGTCGAACACCGTGTTGTCACAAATCCACGAGTTG
AAAGATTCTCAACAGCATATTTCTTCTGTCCTTCCTATGATACGGTGATACAAAGTTGCTATGAGCCTTC
AGTTTATAGGAAATTTAGCTTTAAAGAATATAGACAACAAGTGCAAGAGGATGTTCAAAAATTAGGTCAC
AAGATAGGGCTCCCTAGGTTTCTTGTATAATACTACTAAATAAATTTTGTGATTAATTAAGTAGGTTTTC
TTTTCTTTTTTCCAAGAAAGAAAA

>*PtrGA2ox8-2*   LOC7485437
TGATGGTCTAGCTTTTCCTCTCTATAAAATATCTCCCTCCAAGCTTAGTGGTCGACACAATACCCTTTCT
CTAACTCCCTCCTTCCCTCTCTCACTCTCTTTCTTTTTCTCATAACTCATTCACATCACAAACGCTTTAA
TTCAAGTGCATATCAAGGAAAGCCTAAATAGTGGAATCCAACAACTTTTTGCCGATCATAAACGAGGATA
AAAGGATGGCCATGGACCCACCTTTCCTAGAGACCTACAAGACCCTCTTGGACAAAGCCACAGAAGGTGC
TCATGGTCATAAAGAGGTTGTTATTATTGAGGAATGTGAGCTTCCATTGATTGATCTTGGCCGTTTAAAT
CTTGGAAAATTGGAGAAGGAAAAGTGCAAGTCAGATATTGCTAGGGAATCACAAGAGTGGGGGTTCTTTC
AAGTTGTGAATCATGGGATTTCACGAGAGATTTTGGAGAAAATGATGAGCGAACAAGTTAAGGTTTATAG
ACAACCATTTAATAACAAGAGCAAAGAGTTATTTAATTTCTCTAGTGGGACTTATCGATGGGGGACACCT
ACAGCTACTTGCCGTGAGCAGTTGGCATGGTCTGAAGCTTTTCATATTCCTATGAATGACATACCGTTTT
CCAATGGTTTCAGTAGTCTCAGCTCGACAATGGAACAATTTGCTACAACTGTTGCCGATCTAGCACAAAA
ATTAGCTGCGATTTTGGCAGAGAAGTTAGGCTTCAAATCAAATTTCTTTCAAGAAAACTGCCTGTCAAGC

ACTTGTTATCTACGAATGAACCGATACCCGCCTTGTCCAATCCCTTCAGATGTATTTGGCCTTATGCCAC
ACACTGACAGTGACTTTCTCACAATATTGTACCAAGATGAAGTTGGAGGATTGCAATTAGTGAAAGATGG
GAAATGGTTCGCTGTCAAGCCTAATCCTGAGGCTCTAATAGTCAACATTGGAGACTTATTCCAGGCTTGG
AGCAACGATGTTTATAAGAGTGTCCAACACCGTGTTGTCACAAATCCACGAGTTGAAAGGTTCTCAACAG
CATATTTCTTCTGTCCTTCATATGATACTGAGATACAAAGCTGCTATGAGCCTTCAGTTTATAAGAAATT
TAGCTTTAGAATGTATAGACAACAAGTGCAAGATGATGTTAAGAAATTAGGTCGTAAGGTAGGGCTCCCA
AGGTTTCTTGTATAATAAATAATTTCTGTGATTAATTAATGAAGAAAATGTACTTTTGGGAAATGAAAAT
ATATAGTTATTAACAATTTTTAATATAATTAAATTATAGCTTGGTAGCGA

>*PtrGA2ox8-3*   LOC18095907
TCAAAGGGCATCGATTCTAGACTATGAACAGGTCTGGTTGCACAACAGGGTACAGCCAATGTGTGCAAAC
ACCGCAGGTTGCCATAAGCGGTTGCATGCCATTACCAAGTTTGTACTATACTGCACTCCCAAGTTTTCTT
GCTCTATTTATACTGATGGATTACACATCTTATATCTTCAATTTTGACAAGGATCACATTCTATCCTTTA
CCTCATCCGTCCCATCTCTCCCTTCATAAAAATGGATCCTCCATTTCAAGAGAAATACAGATCCCTCTTC
AACGATTATACCATAGTATCAAAAGACAAGGATGATAGTCTAATGAATGCTAATGATGAGTGTGAGCTGC
CTCTCATAGATCTTCATCGTTTGACACTTGAATACTCGGAGAGAGAGCAATGTGTAAAGGAGATAAAGCA
GGCTGCAAGTGAATGGGGATTCCTTCAAGTTGTCAATCATGGGATTCCACAAGAGATGTTGAAGAGCCTT
CAATACGAGCAAAGGAAGGCTTTCCAACATCCGTTCAGAAAAAAGGCTGAAGACAACATTCTGAATTTGT
CTGCAAATAGTTACAGATGGGGAAACCCTAGAGCCACTTGTTTGAGGCAGCTCGCATGGTCAGAAGCCTT
CCACGTACCTCTCACTGATATTTCAAGAATCGGTGATGCATACAAGAGTCTCAGTGCAAGCATTGAAGCT
TTCACAACAACAGCTAACGCATTAGCTAAAGGCGTGGCAGAAATTCTGGCTGAGAATCTTGGAGTTTCAT
CCACTTTCTTTGAAGAGAATTGTCCAGAAGAAACTAGTTATCTTCGAATGAACAGATATCCTCCATGTCC
ATTCTCTTCTGAGGTCTTTGGCTTGATACCTCATACCGATAGTAGTTTCCTCACTGTACTAAATCAAGAT
CAGATTGGAGGATTGCAACTATTGAAAAATGGAAGATGGATTAACGTTAAACCTAATCCAGAAGCTCTAG
TGATTAATATTGGTGACTTATTCCAGGCATTGAGCAATGATGTTTACAAAAGCATCAAGCATCGGGTGCT
TGCCCCCCAACAAGTTGAAAGGTTTTCTTTGGCATTTTTCTATTGCCCCACTTATGAGACTGTGATAGAG
AGTAGCATCAAGCCATCAAAGTATAAGGAGTTCACATTCAGAGAGTTTATGATGCAGATTCAAAGGGATC
TTAAAGCTACTGGTGATAAAGTTGGAGTCTCAAGGTTTCTTCTGTGAACCTGAGCCCCTGTGTGTGCTGG
AAAATTTCAAGATAGAATAACTAGTTAAATCAAAGTTCCCTCTTCGTACTGTTTCGATTATTTGAACTGC
AACTTAATTGGTAATTTCCATTTCTAAATTACATCGATCAAATGTTTTTGCTTTCTTTTGT

>*PtrGA2ox8-4*   LOC7462625
CTCTATGACAATTCCAGAGTGAAATCCTCTATCTGTGACAACTGTCACGATTTAGGAGCTCTCCTCTTGT
TCTCTTCTCTTTGTGTTCCTTGTCCTCGCCAATTCCTCCCCCTTGGACTCTCTATATAAAACGCACTTGC
CACCTTTGAATCCCATCATGCACGATCTTACAAGCCAACCAATAATGATGATTGATTCAAGCCCACCTCT
TCTACACCATTATGGAGAGCTTACACGCCTCCCACCCCAAATTCCTACACTTGAATGCAATGACCTCCCA
AGTGGTGCTGTTGTCATGAAGGAATATTGCCAACTCCCATTGATAGACCTCAGTTGTCTAAATAGCACCA
TTGAAAGGGAACGGCTAGCTTGTGCTGAGGCTATATGCAGAGCTTCGTCGGAGTGGGGATTCTTCCAAGT
AGTGAACCACGGTATAAGCCCCGAGCTAGTTCGGAATATGAGGAGGGAACAAGTGGAGTTGTTTCAAACA
CCTTTTGATAAGAAGGCTACTTGTGGGGTTTTGAACAACTCTTATAGATGGGGAACCCCAACAGCTACTT
GTCCAAGGCAATTCTCTTGGTCTGAAGCTTTCCACATTCCTCTCTCAAGAGTTTCTGAGCAAGCTTGCTA
TGGGGAGTTTAGCTCTTTAAGGGAAGTGATGATGGAATTTGCAGCAGCAATGTCAAAGCTAGCAAGGGTG
CTGGCAGGGGTCCTAGCAGAAAATCTAGGCCACCCAAGAGGAGTGTTTGAAAACATTTGTCAAGAAATTA
ATTGCTTTCTTCGGTTGAATCGCTACCCAGCCTGCCCAATATCCTCAGAGATTTTTGGCTTAGTGCCCCA

CACTGATAGCGATTTTCTAACAATCCTTTCTCAAGATGAAGTTGGAGGGCTGCAACTTATGAAAGATTCC
AAATGGGTGGCTGTAAATCCGAATCAAGATGCTCTTATCGTCAACATTGGAGATCTTTTCCAGGCATGGA
GCAATGATGTCTACAAGAGCGTGGAGCATAAAGTCGTGGCTAATGGAAAAATGGAGAGATATTCAATCGC
CTACTTTCTGTGCCCCTCTTATGACTCATTGATAGGCAGCTGCATGGAGCCTTCTATATACAGAGAGTAT
AGGAGCCAAGTCCAAGAAGACGTCAAAAGAACGGCCGTAAGATAGGCCTTCCAAGATTTCTACTTTAAC
ACATGTCGTCGATAAAAAAGATGTTCAGCATTTAGGTATCAAAAGATTCAAAACACAACATTCGAAGCAG
CT

>*PtrGA2ox8-5*   LOC7454413
GTTATATAAACTGATGAAATACTCATTTTATTTCAAAACTTGAGAAAATCACATTCTTTCCTTCACCCT
CATTCGTCCCTCTCCCCTTCATAACACAATGGATCCTCCATTTGAAGAGAAATACAAATCCCTCTTAACC
AATACTACTCTATTATCAAAAGACAAGGATTATGTTATAATGAGTGATTACGAGGAGCATGAGCTGCCTA
TCATAGATCTTCACCGTTTGACTCTCTCCTTCTCGGAGAGAGAGCAGTGTGTAAAAGAGATAAGGCAGGC
TGCTCGTGAATGGGGCTTCTTTCAAGTTGTGAATCATGGGATTCCACAGGAGATTTTGGAGGGCATTCAA
CTCGAGCAAAGGAAGGCTTTCCATCATCCATTCAGCAAAAAGGCTGAAGAAAACATTTTGAATCTGCCTG
GTTACACATGGGGTAACCCTGCAGCCACTTGTTTGAGGCAGCTCTCATGGTTAGAAGTCTTCCACATACC
TCTCACAGATATTTCAAAAATCAGTGGTGAATACAAGAGTCTCAGAGAGAGCATTGAAGCTTATACAGCA
ACTGCTGAAAAACTGGCTAAAGACCTGTCAGAAATTCTAGCTGAGAATCTGGGTGTTTCCTCCACTTTCT
TTCAAGAGAATTGTCTGCCGGAAACTAGTTATCTTCGAATGAACAGATACCCTCCATGTCCATTCTCTTC
TGAGGTGTTGGGCGCGCTGCCCCATACCGATAGCTGTTTCGTCAACGTACTAAATCAAGATCAGATTGGA
GGATTGCAACTATGGATGAATGGAAAATGGATTAGCGTTAAACCTAATCCAGAAGCTCTAATAATTAATA
TCGGTGACTTATTTCAGGTAGCGAGCAACGATGTTTACAAAAGCATAAGGCATCGGGTACTTGCCTCCAA
ACAAGCTGAAAGGTTTTCTTTGGCATATTTATATTGCCCCAGAAAAGATGCTGTCATAGAGAGTGGAATG
AAGCCATCAATGTACAGGAAGTTCACATTCGGAGAGTTGACGGAGCAAAATGCAAGGGATGTTAAGGAAA
CAGGCAACAAAGGCGGGATCCCAAGGTTTCTCATGTGAATGAGCATATTGTGCTGGAACGTTTCATCAGG
ACTGAATAAAATTTTATTATTAGTATATTAATCTCACATGGAATAAAGTTTGCTTGTTTGTTTTTCGATT
ATCAAATTCAATAAGTATATTTGGTTATTTAAATTGTAATTTAATTGAACATTTCAGCTTCTATAATATA
TGAATAC

>*PtrGA2ox8-6*   LOC7471474
AAAGGCCCCTACAAATTAATTAAATAAACATTTTTCAACAAAGTCAAGAGAGAGAGAGGGAGAGAGGGAC
AAGGTAGGTTTGGCTTCCGTTCATATTGTCATCTCCCACTCCCGCTGTTAAGCTTTCTCATTTCTGGCTG
TTCATCAAACATTGTTTTTACTGTATCTTTGTTAAGACTTGGTCTCTCCCTCTCTCTGTCTTTCACAATT
CCAGAGTGAAAATCCTCCCCTTGTGACAACTGTCACAATTCGGGAGCTCTCCTCTTGTTATTTCCTCTTT
GTGTTCCTTGTCCTCATCAATCCCTCCCCCCTTGAAATCTATATAAAAGCACTTGCCATCTTTGAATCCT
ATCATGCACGATCTTACTAGCCAACCAGTAAGTATGATTGATTCAAGCCCACCTCTTCTTCGCCATTATG
GAGAGATTTCACGCCTCCCACCTCAGATTCCTACACCCGAACGCAGTGACCTCCCAGATGGTATTGTTCT
CATGGAAGAATACTGCCAACTCCCTTTGATCGACCTCAGTTGTCTAAGTAGCACCAACGAAAAGGTGCGG
CTAGCTTGTGCTGACGCTATATGCAGAGCTTCGTCGGAGTGGGGATTCTTCCAAGTAGTGAACCATGGTA
TAAGCCCTGAGCTGGTTCGGAATATGAGGAGGGAGCAAGTGAAGTTGTTTCAAACACCTTTTGATAAAAA
GGTGACCTGTGGGGTTTTGAACAACTCTTATAGATGGGGAACTCCAACAGCTACTTGTCCAAAGCAATTC
TCCTGGTCTGAAGCTTTCCACATTCCTATCTCAAAAGTTTTTGAGCAAGCTTGCTATGGGGAGTTTAGCT
CTTTAAGGGAAGTGATGGTGGAATTTGCAGCAGCAATGTCAAAGCTAGCAAGGTTACTGGCTGGGGTCCT
AGCGGAAAACTTAGGCCACCCAAGAGGAGTGTTTGAGAGCACTTGTCAAGAAAGCAATTGTTTTCTTAGG

TTGAATCGTTACCCAGCCTGTCCAATATCATCAGAGATTTCTGGCTTAGTGCCCCACACTGATAGCGATT
ATCTAACAATCCTCTCTCAAGATGAAGTGGGAGGGCTACAACTCATGAAAGATTCAAAATGGGTGGCTGT
TAAACCCAATCCAGATGCTCTTATAGTCAACATCGGAGATCTTTCCCAGGCATGGAGCAATGATATCTAC
AAGAGCGTGGAGCACAGAGTCACGGCAAATCGAGAGAAGGAGAGATACTCAATCGCCTACTTTCTATGCC
CCTCTTATGACTCGTTGATAGGCAGTTGCAGAGAGACTTCTTCTATATACAGAAAATTCACTTTCGGAGA
GTACAGAAACCAAGTCCAAGAAGACGTCAAGAGAACAGGCCGTAAGATAGGCCTTCCAAGATTTCTACTT
TAACACATACCATCCATTAACAAAATCTTCAGCCTTTAGGTATCAAAACACAGCATTCAAAGCAGCTGAA
TAGATAAGCAGGAAGATGATATATATATATATATAAAATGTATGTATTATTCATGTTGAAGTCAAGTCTG
ATTCCGCCAGTATGTGCATTGATGTAGTTAAGCCCAATGAAAATCTGCCACCCTTCAGTTTATCGGAGAC
ATACAGACTCAAGTCTGCCATTTCAGAAATAGTTCGATAGCCCATTGTTCAAAATCACGTAGCCAAATGG
CCCATCAACGGCCCGGTCTATGAGTAAGTTTGTTGCTTGGATTAAAGAGCCTCCCTAGAGATGTCCTCTC
TCCCGCCTACAGACTGTACCAATTTCTATTAACCTTATGAATCTATTTCTTCACCAAATATACACGCATG
ATCTAGTCGTTGAATTTATACCATGTATGTGAAGAGACTGCTGCATTATGTGGCTGCATGCGAAGAAGAA
TAGATGGATATCATGTGTCCTGTTGCCCTT

>*PtrGA2ox8-7* LOC18103428

TCACATTCTTTCCTTCACCCTCATTCGTCCCTCTCCCCTTCATAACAATGGATCCTCCATTTGAAGAGAA
ATACAAATCCCTCTTAGCCAATGCTACTCTATTATCAAAAGACAAGGATGATGTCATAATGAGTGATTAC
GAGGAGTATGAGCTGCCTATCATAGATCTTCACCGTTTGACTCTCTCCTTCTCGGAGAGAGAGCAATGTA
TAAAAGAGATAAGGCAGGCTGCAAGTGAATGGGGCTTCTTTCAAGTTGTGAATCATGGGATTCCACAGGA
GATTTTGGAGCGCATTCAACTGGAGCAAAGGAAGCTTTTCCATCATCCATTCAGCAAAAAGGCTGAAGAA
AACATTTTGAATTTGTCTGAAAATAATGGTTACAGATGGGGTAACCATACAGCCACTTGTTTGAGGCAGA
TATCATGGTCAGAAGCCTTCCACATACCTCTCACAGATATTTCAAAAATTGGTGGTGAATACAAGAGTCT
CAGAGAGAGCATTGAAGCTTACGCAGCAAGTGCTGAGAAATTGGCTAAAGAGATGACAGAGATTCTAGCT
AAGAATCTGGATATTTCCTCCACTTACTTTCAAGAGAATTTTCTGCCGGAAACTAATTATCTTCGAATGA
ACAGGTATCCTCCATGTCCATTCTATTCTGAGGTGTTTGGCATACTGCCTCATACCGATAGCTGTTTCGT
CAATGTATTAATTCAAGACCAGATTGGAGGATTGCAACTTCGGGTAAATGGAGAATGGATTAGCGTTAAA
CCTCATCCAGAAGCTCTACTAATTAATCTTGGTGACTTATTTCAGGCATTGAGCAATGATGTTTACAAAA
GCATTAGGCATCGGGTAGTACTTGCCTCCAAACAAGTTGAAAGGCTTTCTTTGGCATATTTATATTGCCC
CAGAAATGATGCTGTCATACAGAGTGGAATGAAGCCATCAATTTATAGGAAGTTCACATTCGAAGAGTTG
ATGAAGCAAAATTCAAGGGATATTGAAGAAACTGGCCGCAAACTCGGGATCTCAAGGTTTCTCATGTGAA
TATGAGCATATTGTGCTGGAACATTTGATCAGGACTGAATAAAATTCAAGGGATATTGTGCTTTTTTTTT
CTTCTGATAGGGAAACATTCTTCTGAAATAGTAAATTGTGGAACAATAAGTTTGTTTGTTTTTCAATTGG
AGCAGGAAGTGGAGTTTCTAGACC

>*PtrGA2ox8-8* LOC7496781

CTCTGCTTATCCTTTTGGTTTGAAGGCCAAAGAAAATAAGCATGTCTGAAATACTAAACCTTAAATCCTA
CCCTCCCGTGTTTCGCCAACAATACATCGGAATCCAACAAAACTCCGGCCTTGACGATACCACCGAACAG
ATTCAAGAAGTTGTTAACGATGATGCCATTCCGGTATTAGACTTTCAGTGCCTAGACCTGGGCAAGCTTC
AAGAGGCTTGCGAAGATTGGGGATTGTTTCGTTTGGTCAATCATGGGATTCCTCTAACCCTTATGAGCCA
GCTTAGAGACCATTCCAGAAACCTTTTTTCTCTTACTTTTGAATCAAAACAAGAACTGTTCACTAACCCC
ATGTCGTATTTTTGGGGCACAACTGCCCTGACTCCAACCGGGGCAGCTCTCTCAATAGGACCACAAAACA
TCAACTGGGTTGAAGGTCTCAATATCCCACTGAGTCAACTCTCTCTGTTTCAAAAAGAAAATGAAACGCT
TGGTTCGTTCAGAGTTTTGTTGGAAGAGTATGGAGGGCACCTAGCTAGACTTGCAACAACCATGTTCGGA

GCAATGGCCAAAAACCTCCATCTTGATCCTGAACTTTCAAAAACTTACATTTCAGAATCAACCGGATTCG
TACGTGTTTACCGATACCCTCAATGCTCTATGGAAAATGAGGCCTGGGGCATAAATGTGCACACAGACAG
CTCAGTTCTGTCAATATTGAACCAAGACCAAGTGGGTGGACTTCAGGTTCTCAAAGATGATAACTGGCTA
CAAGTAAAACCCATTCCTGACACACTGGTTTTCAACCTTGGAGATATGATGCAGGCCATAAGCGACGACA
AATACAAGAGTGTGAAACATAGAGTGAAGGTGAACAAGGAAAAGGAGAGATTCTCCATCTGCTACTTTGT
CTTCCCCGCTGAAGGCAGTGTCATACAGAGCTCAAAGTACAGGCCTTTCACATACAGTGATTTCCAAGCA
CAAGTGCAACAAGACGTAAAGACACTAGGATTTAAAGTTGGGCTTGAGAGGTTCAAGGTTGCTGGTTGAT
TATTATTATTTTTTCTCGCAAGTCTCCTCCTCTAATTTATTTAGACAGGTCTGTTTTCCAATTACAAACA
AATGTTGAAGATGACATATCAATTATCGCTATCTGTTCCGCTTAGTGGAAAACTGTGTGAAACTACATCG
AAGGAGAACTAGATTTCGATTGTGATGGCATTGGTACGTACGGCGGGACAAAATAAGCTGGCAAGCTGAG
ACATCAATCTTTTCATGTGGTTTGACACACGGTGGGCCCGTTCTTCTGTATTAAAAATAAATAAATAAAT
AAATATAAGCCCATCTCTTGAAATCATGCATCTGAGATCATTTGGGCCAACCCAATTATTTA
